# Evolution of Recombination Landscapes in Diverging Populations of Bread Wheat

**DOI:** 10.1093/gbe/evab152

**Published:** 2021-06-29

**Authors:** Alice Danguy des Déserts, Sophie Bouchet, Pierre Sourdille, Bertrand Servin

**Affiliations:** 1 INRAE-Université Clermont-Auvergne, UMR1095, Génétique Diversité Ecophysiologie des Céréales, Clermont-Ferrand, France; 2 INRAE, Université de Toulouse, GenPhySE, Castanet-Tolosan, France

**Keywords:** recombination, evolution, bread wheat

## Abstract

Reciprocal exchanges of DNA (crossovers) that occur during meiosis are mandatory to ensure the production of fertile gametes in sexually reproducing species. They also contribute to shuffle parental alleles into new combinations thereby fueling genetic variation and evolution. However, due to biological constraints, the recombination landscape is highly heterogeneous along the genome which limits the range of allelic combinations and the adaptability of populations. An approach to better understand the constraints on the recombination process is to study how it evolved in the past. In this work, we tackled this question by constructing recombination profiles in four diverging bread wheat (*Triticum aestivum* L.) populations established from 371 landraces genotyped at 200,062 SNPs. We used linkage disequilibrium (LD) patterns to estimate in each population the past distribution of recombination along the genome and characterize its fine-scale heterogeneity. At the megabase scale, recombination rates derived from LD patterns were consistent with family-based estimates obtained from a population of 406 recombinant inbred lines. Among the four populations, recombination landscapes were positively correlated between each other and shared a statistically significant proportion of highly recombinant intervals. However, this comparison also highlighted that the similarity in recombination landscapes between populations was significantly decreasing with their genetic differentiation in most regions of the genome. This observation was found to be robust to SNPs ascertainment and demography and suggests a relatively rapid evolution of factors determining the fine-scale localization of recombination in bread wheat.

## Introduction

Significance

Recombination is the fundamental biological process that shuffles chromosomes during meiosis to create new allelic combinations in gametes. It has been shown to be controlled by genetic factors in some species but those remain unknown in many. Understanding the genetic determinism of recombination can help to better describe its underlying biological make up, its constraints and understand its evolvability. One approach to study this question is to evaluate if the recombination process differs between groups of individuals that are genetically distinct (populations). Here we propose methods to implement this approach and investigate the recombination process in bread wheat, one of the most widespread crops. We show that recombination patterns between two populations are increasingly correlated when their genetic differentiation decreases. This suggests that recombination in bread wheat can evolve rapidly possibly associated to an underlying modification of its genetic determinism.

Meiotic recombination (or crossover; CO) is the obligate genetic exchange between homologous chromosomes that occurs during the production of gametes in sexually reproducing species. Besides its role in ensuring proper segregation of chromosomes in gametes, it also impacts evolution by breaking linkage between advantageous and deleterious alleles and by creating novel combinations of alleles (Barton 1995; Charlesworth and Barton 1996; Otto 2009). Recombination rates are highly variable between species and also at different genomic scales. At the chromosomal level, COs are not evenly distributed depending on either the size of the chromosomes, the region of the chromosomes or on interference. Interference was first observed in *Drosophila* (for review, see Berchowitz and Copenhaver 2010) and is defined as the impossibility for a type I CO (i.e., COs that are submitted to interference contrary to type II COs that are not) to occur in the vicinity of another CO from the same type. Type I COs are thus more regularly spaced along chromosomes than expected from random (Zickler and Kleckner 2015). Within chromosomes, some regions are also deprived of COs, such as centromeres of all species studied so far. Moreover, in many species, distribution of COs is skewed toward telomeres (Haenel et al. 2018). In wheat (*Triticum aestivum* L.), for example, more than 80% of the recombination events occur in the terminal regions of the chromosomes representing less than 20% of the genome (Saintenac et al. 2009; Choulet et al. 2014; Darrier et al. 2017; International Wheat Genome Sequencing Consortium IWGSC 2018). The main hypothesis is that the initiation of synapsis responsible for recombination occur in the telomeric regions as shown in barley (Higgins et al. 2012; Dreissig et al. 2019). In species with small chromosomes such as *Arabidopsis thaliana* or rice (*Oryza sativa*), recombination events are more evenly distributed along the chromosomes with the exception of the centromeres (Choi et al. 2013; Drouaud et al. 2013; Marand et al. 2019). In all studied species, the number of COs per chromosome and per meiosis is rarely superior to three (Mercier et al. 2015).

At a local scale, in most species including yeast, birds, snakes, fishes, mammals, and plants, COs mainly occur in small regions of a few kilobases (kb) called hotspots (Myers et al. 2005; Mancera et al. 2008; Choi and Henderson 2015; Singhal et al. 2015; Shanfelter et al. 2019; Schield et al. 2020). In some mammals, these hotspots are determined by PRDM9, an SET-domain protein with a zinc-finger array that binds DNA (Boulton et al. 1997; Oliver et al. 2009; Baudat et al. 2010; Myers et al. 2010). PRDM9 recognizes specific DNA motifs and deposits an epigenetic landmark (histone H3 trimethylated on lysine 4: H3K4me3) that is further recognized by the machinery forming double-strand breaks that initiates COs (Murakami et al. 2020). However, many if not most species (e.g., birds, plants, yeast, snakes, and fishes) do not exhibit a PRDM9 derived mechanism. Recombination hotspots are often found in accessible chromatin regions and mainly driven by chromatin features (Auton et al. 2013; Choi and Henderson 2015; Singhal et al. 2015; Marand et al. 2017, 2019) although intermediate situations exist (Schield et al. 2020).

The determinism of local recombination rate considering the distribution of CO hotspots remains unknown for many organisms. One approach to better understand this determinism is to characterize the evolution of the recombination landscape and evidence its conservation or lack thereof. This can be achieved by contrasting recombination landscapes in closely related species (Stapley et al. 2017) or in differentiated populations of the same species (Kong et al. 2010; Salomé et al. 2012; Petit et al. 2017). For example, in rice, less than 20% of the CO hotspots are common between the two subspecies *Oryza sativa ssp. japonica* and *O. s. ssp*. *indica* (Marand et al. 2019) although they diverged relatively recently [440,000 − 86,000 years ago (YA); Ma and Bennetzen 2004; Vitte et al. 2004; Zhu and Ge 2005; Tang et al. 2006]. Similarly, in the cocoa-tree (*Theobroma cacao*), only little overlap of recombination hotspots was observed across ten diverging populations, with less divergent populations showing higher level of overlap (Schwarzkopf et al. 2020). Note that recombinations tend to cluster in more distal regions in domesticated barley (*H. vulgare*) compared with wild barley (*Hordeum vulgare ssp. spontaneum*) (Dreissig et al. 2019) while domestication began approximately 10,000 YA (Badr et al. 2000). A finer-scale analysis among subpopulations of wild barley revealed that recombination rate varied according to environmental conditions (temperature, aridity, solar radiation, annual precipitations), suggesting that environmental factors might explain part of these differences (Dreissig et al. 2019).

High-density genotyping SNP arrays as well as new generation sequencing (NGS) approaches now allow to analyze large collections of wild/domesticated, ancient/modern populations of both animals and plants. Such a large amount of accurate data permits to better decipher the recombination landscape from patterns of linkage disequilibrium (LD) (Li and Stephens 2003; Auton and McVean 2007; Chan et al. 2012). The advantages of using this approach stem from the large number of meiosis that occurred during the evolution of sampled populations compared with bi-parental or multi-parental experimental populations. First, as LD-based recombination inference is based on recombination happening in many different individuals it should consequently be less sensitive to individual specific variation, which might occur in the presence of structural variation (e.g., Bauer et al. 2013; Rowan et al. 2019). Second, LD-based recombination rate estimates are more resolutive as genetic diversity is higher compared with experimental segregating populations that typically involve few parents. However, the drawback of this approach is that the recombination landscapes obtained have to be interpreted cautiously as they can be affected by evolutionary forces such as selection and demography that can also impact local patterns of LD (Charlesworth and Charlesworth 2010; Auton and McVean 2012; Choi and Henderson 2015).

Despite these limitations, the LD-based approach was successfully applied at the whole-genome level in many species including birds (Singhal et al. 2015; Smeds et al. 2016), yeast (Tsai et al. 2010), Arabidopsis (Choi et al. 2013), rice (Marand et al. 2019), and barley (Dreissig et al. 2019). In bread wheat this approach was used to study recombination pattern on chromosome 3B (Darrier et al. 2017), the only chromosome presenting a sufficiently high-standard reference sequence at that time (Choulet et al. 2014; IWGSC 2014). The analysis of two collections representative of the Asian and European genetic pools revealed a high similarity between their recombination profiles. These LD-based profiles were also shown to be consistent with a meiotic recombination profile derived from a bi-parental population (Chinese Spring × Renan; Choulet et al. 2014). This result suggested that recombination rate estimation through a LD-based approach could be even more informative and resolutive along the whole genome using the last gold-standard reference sequence available (IWGSC 2018), as well as high-density genotyping of large wheat collections.

The complexity and huge size (16 gigabases) of the wheat genome have long hampered the development of high throughput genomic tools as well as the establishment of a whole-genome sequence. Bread wheat is an allo-hexaploid species (AABBDD; 2*n* = 6*x* = 42) derived from two successive interspecific crosses involving three diploid species (for details, see https://www.wheatgenome.org/; IWGSC 2014, 2018): *T. monococcum ssp. urartu* (AA genome), a yet-unknown species related to the *Sitopsis* section (SS genome related to the wheat BB genome) and *Aegilops tauschii* (DD genome). However, international efforts combined with appropriate and original strategies using chromosome sorting, chromosome-specific BAC libraries, paired-end short-read sequencing and relevant assembly approaches, led to the publication of a high-standard, annotated, oriented and anchored sequence of the wheat genome (IWGSC 2018). At the same time and despite the presence of a high proportion of transposable elements (85%; Wicker et al. 2018), high-density SNP arrays have been successfully developed and used for marker-assisted selection (Sun et al. 2020) and for the characterization of collections (Winfield et al. 2016; Balfourier et al. 2019). In the study of Balfourier et al. (2019), the genetic structuration of 4,506 bread wheat landraces and cultivars representative of the worldwide diversity was described using the TaBW280K SNP chip. These LD data offer the opportunity to extend previous work on bread wheat by analyzing recombination along the whole genome and across more populations. We compared the ancestral recombination profiles of four populations with the meiotic recombination observed in a biparental population of recombinant inbred lines (RILs; Chinese Spring × Renan; CsRe). We developed specific statistical models to evaluate and minimize the influence of evolutionary forces on the comparison of recombination landscapes between populations.

## Results

### Bread Wheat Landraces Are Structured in Four Main Populations

**Fig. 1.**
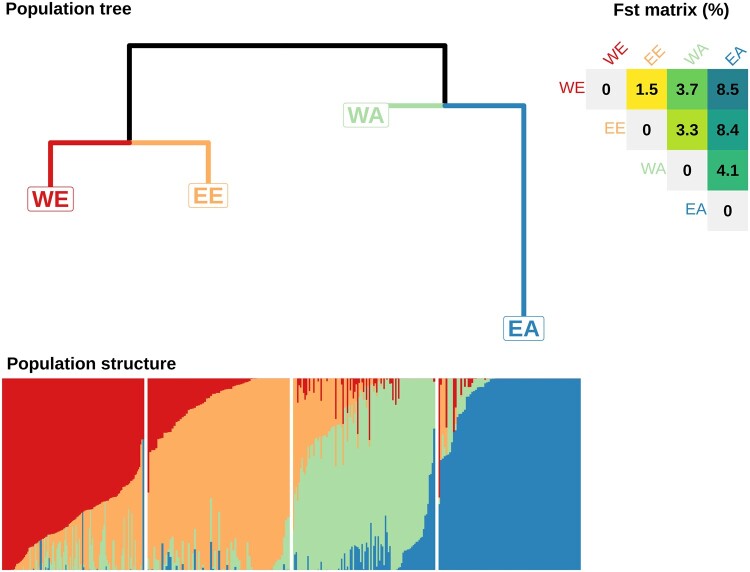
Bread wheat landrace genetic divergence and structuration. Population tree: Neighbor Joining tree built with pairwise Reynold distance matrix computed on SNP alleles and rooted by HAPFLK software (Bonhomme et al. 2010; Fariello et al. 2013). WE, West Europe; EE, East Europe; WA, West Asia; EA, East Asia. Fst matrix (%) Weir and Cockerham pairwise *F*_ST_ computed with simple matching distance of haplotypic alleles. Population structure: Admixture coefficients for *K* = 4 from Balfourier et al. (2019) using STRUCTURE software and haplotypic alleles.

Establishing LD-based recombination maps requires samples of unrelated chromosomes from a homogeneous population. We extracted a subset of 371 landraces representative of the worldwide diversity from Balfourier et al. (2019), forming four distinct and mostly homogeneous genetic populations (see Materials and Methods; fig. 1) that were named according to the geographical origins of their members: The West-European population (WE), composed of 127 accessions originating from France (52 accessions), Spain (10), Germany (8) and from 30 other Western European, Mediterranean countries and Iberian peninsula; the East-European population (EE), composed of 70 accessions originating from France (9), the Russian Federation (7), Ukraine (5) and from 27 other Eastern European countries; the West-Asian population (WA), composed of 97 accessions originating from Afghanistan (8), Pakistan (8), Turkey (8) and from 33 other of Caucasian and Central Asia countries and Indian peninsula; the East-Asian population (EA) composed of 77 accessions originating from China (61), Japan (7), the Republic of Korea (4) and from five other South East Asian countries (supplementary file S1, Supplementary Material online).

The genetic differentiation of the four populations confirmed an increasing genetic divergence along an Eurasian gradient (fig. 1), consistent with isolation by distance, selection, and differentiation that occurred during the initial independent spreads of bread wheat from the Cradle of Agriculture and Wheat in the Fertile Crescent toward Europe on the one hand and Asia on the other hand during the Neolithic period (Balfourier et al. 2019). WE and EE are the most related groups (*F*_ST_ = 0.015), whereas WE and EA are the more divergent ones (*F*_ST_ = 0.085) and also the most geographically distant. The WA population is the closest population to the tree root possibly because it includes accessions that were collected not far from the center of domestication of bread Wheat (Fertile Crescent: Turkey, Iraq, Iran; Caucasus and Caspian Sea: Armenia, Georgia, Kazakhstan, Turkmenistan). The EA population appears as a very differentiated and homogenous population. WE and EE are less differentiated because they separated more recently from each other (Balfourier et al. 2019).

The genetic composition of the four populations appeared quite distinct between populations but homogenous within populations when described by the *K* = 4 admixture analysis of Balfourier et al. (2019) (fig. 1). WE, EE, WA, and EA have almost all their members belonging to the same specific dominant group (respectively, named by Balfourier et al. (2019) as North West European, South East European, Central Asian and African and South East Asian groups) with a high membership coefficient: 0.74 on average for WE (standard deviation = 0.16), 0.81 for EE (±0.16), 0.73 for WA (±0.17), and 0.93 for EA (±0.14). The WE and WA populations appear to be more admixed than EE and EA at *K* = 8 (supplementary fig. S1, Supplementary Material online). In order to analyze groups that are large enough to estimate relevant statistics, we split landraces into four populations, although there is some sub-structuration within populations. This was motivated by the fact that the model we used to estimate LD-based recombination rates was shown to be robust to moderate levels of structuration (Li and Stephens 2003).

### Recombination Patterns Are Broadly Conserved across Populations

#### Robust Meiotic Recombination Map of a Population of RILs

In order to obtain a view of recombination patterns that is not influenced by evolutionary forces, we established a meiotic recombination map from recombination events observed in a population of 406 F6 RILs (termed CsRe in the following). This population is derived from a cross between two bread wheat varieties: Chinese Spring and Renan belonging, respectively, to the EA and WE gene pools. The CsRe population was previously genotyped for the same set of SNPs as the landraces (Rimbert et al. 2018). Recombination rates in CsRe were derived from the observed proportion of recombinants in each of the 79,543 intervals defined by SNPs that were polymorphic in the cross. The distribution of recombinants in these intervals led to extremely contrasted situations. On one hand, 60% of these intervals harbored no recombinant among the 406 offspring. On the other hand, a few recombinants were observed in very small intervals. Using a frequentist statistical approach to estimate recombination rates from these observations produces extreme differences in recombination rates that are highly influenced by the limited sample size available. In order to produce more reliable estimates that better account for sample size and uncertainty, we fitted a Bayesian Poisson Gamma model on the observed recombinant counts (see Materials and Methods). With this model, the estimates of recombination rates in the RIL population ranged from almost 0 to 78 cM/Mb among intervals. Compared with the frequentist estimates that ranged up to 2,806 cM/Mb this approach has the advantage of shrinking extreme values that are unrealistic and solely due to the limited number of RILs available. Consistent with the Bayesian model correcting for the effect of sample size, the correlation between frequentist and Bayesian estimates increases with the number of observed recombinants per intervals (supplementary fig. S2, Supplementary Material online), that is, the two approaches converge to the same inference when the data is informative enough.

#### Validation of LD-Based Recombination Maps on CsRe Meiotic Recombination Map

LD-based recombination maps were inferred from patterns of LD between polymorphic SNPs for each landrace population independently using PHASE (Li and Stephens 2003; Crawford et al. 2004). As LD is strongly related to meiotic recombination but can also result from evolutionary forces, those maps were compared with the meiotic CsRe recombination map described above.

Before estimating LD-based recombination rates, SNPs were filtered out on Minor Allele Frequency with a minimum value of 3% within each population, yielding to 170,509 SNPs for WE, 161,137 for EE, 171,901 for WA, and 131,585 for EA. The average marker density was 11 SNPs/Mb with most of the SNPs located at telomeres (25 SNPs/Mb) whereas centromeres were depleted in SNPs (3 SNPs/Mb, supplementary fig. S3, Supplementary Material online). SNP density was almost three times higher on the A and B genomes compared with the D genome (respectively, 14, 14, and 5 SNPs/Mb). This is consistent with the lower rate of polymorphism of the wheat D genome (IWGSC 2018).

**Fig. 2.**
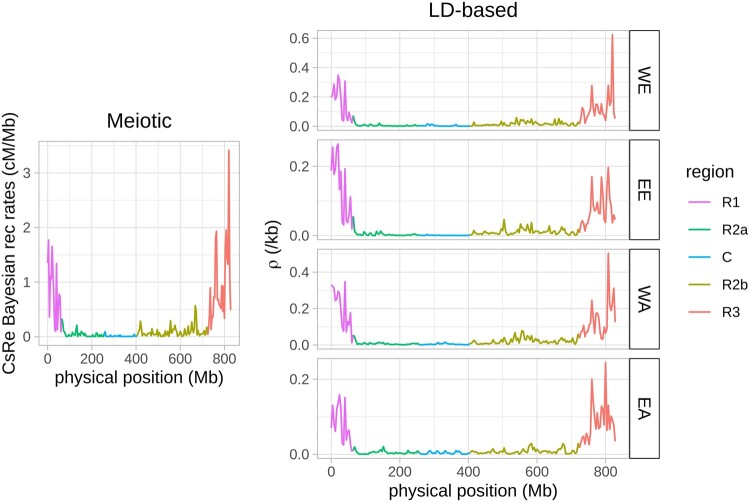
Meiotic and LD-based recombination profiles in 4 Mb windows along chromosome 3B in the CsRe segregating population (left) and in the four West European (WE), East European (EE), West Asian (WA), and East Asian (EA) populations (right). Each color corresponds to genomic regions defined by Choulet et al. (2014): highly recombining telomeres R1 (magenta) and R3 (red); low recombining pericentromeres R2a (dark green) and R2b (light green); and centromere C (blue) where recombination rates are close to 0. LD-based recombination profiles at log_10_ scale are present in supplementary figure S4, Supplementary Material online.

Both LD-based and meiotic recombination profiles showed the same global patterns at the chromosome scale (fig. 2; supplementary file S2, Supplementary Material online). In both approaches, the telomeric regions R1 and R3 of chromosomes showed recombination rates (average LD-based recombination rate in WE = 1e−2/kb; average CsRe Bayesian recombination rate = 0.8 cM/Mb) around ten times higher than the pericentromeric regions R2a and R2b (2e−3/kb; 0.1cM/Mb) and one hundred times higher than the centromeric regions C (2e−4/kb; 0.01 cM/Mb). Recombination rates on the D genome (5e−3/kb; 0.3 cM/Mb) were around 25% higher than recombination rates in the A and B genomes (both 4e−3/kb; 0.2 cM/Mb). The chromosomes from the D-genome are 20% shorter than those from the A or B genomes (IWGSC 2018) while they receive the same number of crossovers (supplementary fig. S5, Supplementary Material online), leading to high global recombination rates. IWGSC (2018) study also showed that the D-genome was twice-less polymorphic than the A or B genomes (18%, 40%, and 41% for the D, A, and B genomes, respectively; IWGSC 2018). It has been demonstrated in maize, sorghum and Arabidopsis that recombination rates are higher in chromosome regions showing higher similarity because a lower genetic diversity facilitates homologous pairing and recombination during meiosis (Rodgers-Melnick et al. 2015; Bouchet et al. 2017; Serra et al. 2018). We can therefore speculate that the high recombination rates we observe on the D-chromosomes are due to their reduced physical size associated with a low diversity favoring recombination.

**Fig. 3.**
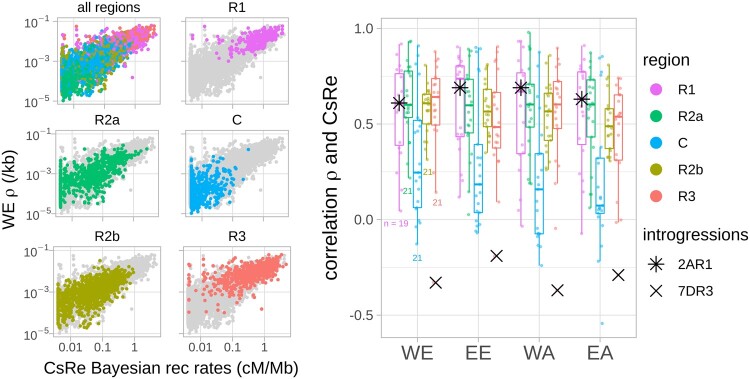
Similarity between LD-based recombination rates and CsRe meiotic recombination rates. Left: Genome-wide relationship between the CsRe biparental population meiotic recombination rates and the LD-based recombination profile of a Western European (WE) bread wheat population. Dots represent the recombination rates averaged within 4 Mb windows. Graphs R1, R2a, C, R2b, and R3 gather recombination rates within the five chromosomic regions defined by Choulet et al. (2014) (R1 and R3 are telomeric regions, R2a and R2b are pericentromeric regions, and C are centromeric regions) of all of the 21 chromosomes (1A, 1B, 1D … 7A, 7B, 7D) of bread wheat. *Right*: Correlation of LD-based and CsRe recombination rates for each landrace population within each genomic region (1AR1…7DR3). Dots represent correlation coefficients of recombination profiles (once averaged within 4 Mb windows) per genomic region and population. Small colored numbers indicate the number of correlation coefficients per boxplot. In principle, each boxplot should contain 21 dots (as many as chromosomes). However, two R1 genomic regions smaller than 20 Mb are not included (4DR1 and 7BR1), because of low robustness of their correlation coefficients (computed on less than five data points). Stars (* and x) represent genomic regions including well documented introgressions in CsRe population.

The genome-wide correlation of LD-based recombination profiles and CsRe Bayesian meiotic recombination profile was quite high for the four populations (≥0.7, table 1) but slightly higher for European populations [pairwise significant differences according Zou’s test (Zou 2007), R cocor package]. These high correlations between CsRe meiotic recombination profile and LD-based recombination profiles are explained by the strong partitioning of the recombination profile along chromosomes present in all bread wheat populations, that is, low recombination rates in centromeres and high recombination rates in telomeres. As computing correlation coefficient using whole-genome recombination profile artificially inflates the value of correlation, we rather performed correlation within each genomic region. The within-region correlation coefficients were lower, but still significantly positive (1AR1–7DR3, fig. 3; supplementary file S3, Supplementary Material online). In telomeres R1 and R3 and pericentromeres R2a and R2b, the average correlation ranged between 0.50 in EA and 0.58 in WE (table 1), with an average of 0.56 across all populations.

**Table 1:** Correlation of the LD-Based Recombination Profiles of the Four Populations of Landraces with CsRe Bayesian Meiotic Recombination Profile

	WE	EE	WA	EA
Genome-wide corr. with CsRe	0.76	0.75	0.74	0.70
Average on 84 genomic regions (R1, R2a, R2b, R3 of chr 1A−7D)	0.58 ± 0.22	0.55 ± 0.28	0.55 ± 0.27	0.50 ± 0.29
Average on 21 C regions (chr 1A−7D)	0.32 ± 0.33	0.30 ± 0.34	0.20 ± 0.34	0.19 ± 0.36

Note.—Recombination rates were averaged in 4-Mb windows.

The recombination rates in centromeric regions showed much lower consistency: The correlation of centromeric LD-based recombination rates and CsRe recombination rates ranged from 0.19 in EA to 0.32 in WE. Considering the low correlation but also the low SNP density and the fact that centromere sequence assemblies are challenging because of the presence of numerous repeated sequences such as transposons and retro-transposons (IWGSC 2018; Wicker et al. 2018), centromeres were no longer included in the analyses.

Among the genomic regions considered, 7DR3 exhibited a strikingly low and negative correlation between LD-based and meiotic recombination rates in all populations (≤−0.19, fig. 3). This result is due to a low recombination rate in part of this region in the CsRe biparental genetic map that is not observed in LD-based maps (supplementary fig. S6, Supplementary Material online). This low recombination rate can be explained by the fact that Renan (one parent of the CsRe biparental population) carries an inter-specific introgression of 28 Mb on chromosome 7D around the eyespot resistance gene *Pch1* coming from *Aegilops ventricosa* (tetraploid species; DDNN) (Maia 1967). This introgression does not recombine in the CsRe cross as this was previously evidenced in another background (Worland et al. 1988). Interestingly the Renan line carries another 20 Mb introgression from *Aegilops ventricosa* in 2AR1 region around the *Lr37/Sr38/Yr17* resistance gene cluster. However, in this region, contrary to 7DR3, the LD patterns are also consistent with a locally low recombining segment in landraces at position of introgression. Because the introgression in region 2AR1 suppresses recombination in an already low recombining segment, this explains why the correlation coefficient with LD-based profiles does not stand out particularly (supplementary fig. S6, Supplementary Material online).

Both CsRe and LD-based maps show a high heterogeneity in the distribution of recombination rates along chromosomes: On average 36% (± 1%) of physical distance represents 80% of genetic distance in all our populations. To further study the distribution of chromosome sites cumulating historical crossovers, we defined highly recombining intervals (HRIs) in the four landrace populations as intervals with an LD-based recombination rate exceeding four-times the background recombination rate (λ ≥ 4, see Materials and Methods). Combining all four populations, this resulted in 8,713 HRIs (among how many intervals?), with a median deviation to background recombination rate λ = 6.5 (range λ = 4 to λ = 511). Note that we avoid here the term LD-based recombination *hotspot* as functional hotspots typically span much smaller genomic regions (size < 5 kb; Marand et al. 2019) than our defined HRIs (median size = 20 kb). Therefore, we cannot be sure that an HRI harbors a single recombination hotspot. The repartition of HRIs along the genome was heterogeneous. Most HRIs (73%) were located in telomeric R1 or R3 regions, and the other HRIs (27%) in pericentromeric R2a or R2b regions. As HRIs corresponded to, respectively, 2% and 1% of intervals in those regions, telomeres were significantly enriched in HRIs compared with pericentromeres (significant chi-square test, *P*-value < 2.2e−16). These HRIs represented 15% of LD-based genetic distance (from 12% in EA to 18% in WA) and around 9% of the physical distance (from 6% in EA to 10% in WE). On average, in all genomic regions, the 8,713 HRIs tend to highly co-localize with open-chromatin features compared with non-HRIs intervals. For example, the proportion of HRIs overlapping genes was 80%, but this proportion dramatically decreased to 53% when considering non-HRIs intervals (supplementary fig. S7, Supplementary Material online). The density of HRIs is also positively associated with the CsRe meiotic recombination rate averaged in 4 Mb windows in each genomic region R1, R2a, R2b, and R3 (*P*-value < 2.2e−16). The proportion of CsRe crossovers overlapping HRIs ranged from 20% in EA to 37% in WE. Most HRIs (82%) overlapped at least one CsRe crossover.

Despite high similarities between LD-based and meiotic recombination profiles within genomic region, there is still the possibility that LD-based recombination rates might be locally influenced by evolutionary forces, such as positive selection, as shown by Petit et al. (2017) in sheep for example. To evaluate the potential effects of positive selection on the LD-based maps, we studied whether a set of genes known to be involved in domestication [e.g., brittle rachis (*Brt*), tenacious glume (*Tg*), homoeologous pairing (*Ph*), or nonfree-threshing character (*Q*)] or recent crop improvement (Pont et al. 2019) were found in regions outliers for the ρ/CsRe ratio. The results showed no evidence of reduced recombination around these genes (supplementary fig. S8, Supplementary Material online). Although this does not rule out potential effects on other genes or through other selection pressures (e.g., background selection), it indicates that strong selective sweeps do not seem to affect recombination inference and justify converting LD-based maps on the meiotic recombination scale (cM/Mb). Considering that LD-based recombination rates are proportional to meiotic ones, they can be rescaled by computing the scaling factor from the CsRe Bayesian average recombination rate in each genomic region (supplementary protocol S1, Supplementary Material online). This produced scaled LD-based maps specific to each landrace population (supplementary file S4, Supplementary Material online).

### Significant Differences between LD-Based Population-Specific Recombination Maps

Our results reveal that the average LD-based recombination rates vary in a 2-fold range between populations: WE has the highest rate and EA the lowest (WE: ρ = 0.004/kb; WA: ρ = 0.004/kb; EE ρ = 0.003/kb; EA: ρ = 0.002/kb; excluding centromeres). This ranking between populations could be explained by genetic diversity levels (fig. 1) as well as by different average meiotic recombination rates. The fact that WE and WA are more admixed populations than EE and EA favored a more important contribution of diversity levels compared with a real difference on average recombination intensity. To eliminate the systematic effect of diversity and demography on recombination rate estimates, we chose to compare the population recombination profiles in terms of the deviation from their local background recombination rates. Specifically, the Li and Stephens’s model (2003) estimates an interval specific recombination parameter (λ) that measures the relative rate of recombination of an interval compared with its neighbors in a 2 cM window (see Materials and Methods). We therefore expect population-specific effects (other than local variation in recombination) to affect the background recombination rate but not the relative intensities of intervals measured by the parameter λ.

The similarity of λ profiles along the genome was evaluated by fitting a linear mixed model on the variations of$\log_{10}\left(\lambda \right)$ within each genomic region, specifying a variance−covariance matrix with different or common correlation coefficients for each pair of populations. In almost all genomic regions (79 out of 84), a lowest BIC was obtained for the model with correlation coefficients that are different between pairs of population(see Materials and Methods). This indicates that local variations of recombination rates are significantly different between populations.

**Fig. 4.**
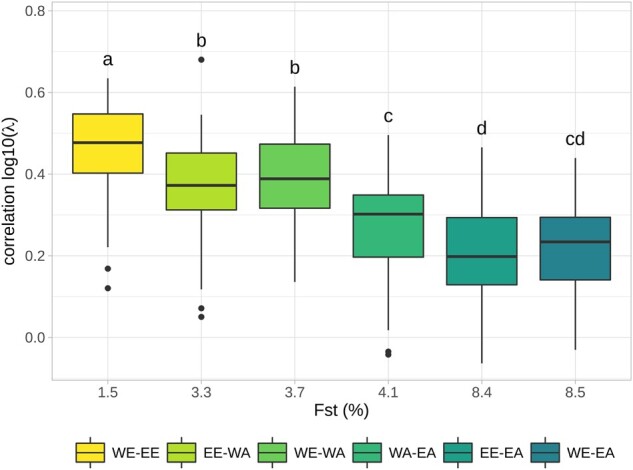
Relationship between pairwise correlation of LD-based recombination intensity λ and *F*_ST_. Each boxplot contains 84 correlation coefficients corresponding to the 84 genomic regions (1AR1…7DR3, excluding centromeres). Letters indicate whether two pairs have significant different average correlations (Bonferroni corrected *P*-value < 0.05).

The average correlation of local variations of recombination rates across genomic regions was twice higher for the highest correlated pair WE–EE (0.47 ± 0.11) than for the lowest one EE–EA (0.20 ± 0.11), with an average value of 0.32 (fig. 4). The unevenness of the distribution of genetic distance along chromosomes between two different populations was measured using a Gini coefficient (Gini 1936). We compared the distribution of recombination in one population with the genetic map of the other. A Gini coefficient of 0 corresponds to a uniform distribution and a coefficient of 1 corresponds to the case where the distribution is a single point mass. In our case, a Gini coefficient of 0 corresponds to identical recombination profiles and the more divergent the distribution in recombination profiles is, the higher Gini coefficient is. The pairwise Gini coefficients increased along the Eurasian gradient, with lower values for closely related population (around 0.43 for WE–EE) and higher values in distant populations (0.77 for WE–EA), meaning that similarity in distribution of LD-based genetic distance along chromosomes decreases along the Eurasian gradient (supplementary fig. S9, Supplementary Material online).

**Fig. 5.**
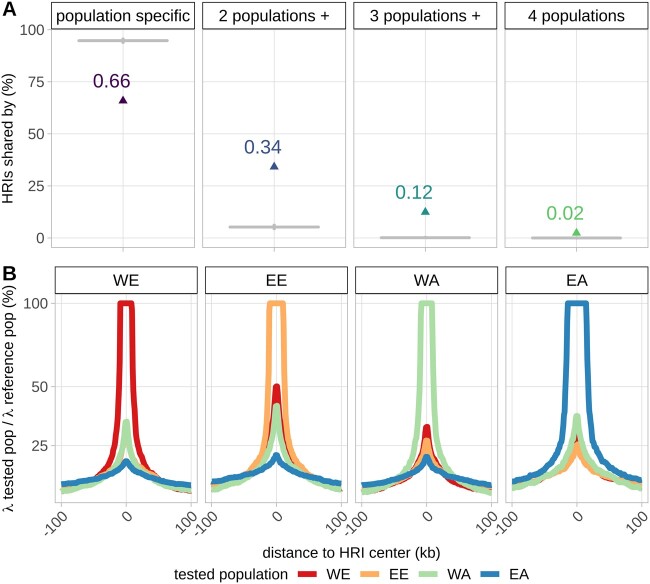
Conservation of highly recombining intervals (HRIs) across landrace populations. (*A*) Proportion of colocalizing HR (colored points) and simulated colocalizing values under random assignment of HRIs (gray boxplots). (*B*) LD-based recombination intensity in each of the four populations WE, EE, WA, and EA around HRIs specific to one population.

In light of these significant differences in the local repartition of recombination events, we investigated whether this could be explained by difference in the localization of crossover hotspots by comparing that of the HRIs (see above). We first defined “hot windows” as genomic regions that harbor an HRI in at least one population. Figure 5*A* represents the proportion of the 5,881 resulting hot windows including HRIs that are population specific (HRI in one population only) or shared by two, three or all four populations. Around 66% of these windows are population-specific and 34% are shared by two populations or more. The proportion of hot windows shared by three or four population drops to 12% and 2%, respectively. Location of shared HRIs along the genome followed the density of HRIs per genomic region. Most (76%) shared windows were located in telomeric regions R1 and R3 and the rest (24%) in pericentromeric regions R2a and R2b (chi-square test *P*-value = 0.06). To check if such an overlap across populations can be explained by chance alone, we compared the observed repartition of hot windows with a simulated distribution obtained by a random assignment of HRIs corresponding to the null hypothesis of the absence of HRI population sharing (see Materials and Methods). The proportion of common hot windows under this random assignment is represented by gray boxplots in figure 5*A*. The observed proportion (colored points) was always significantly different to the expected proportion under random assignment of HRIs. On average, 95% of hot windows are population-specific if assigned randomly, much more than the 66% we observed. In addition, four-population overlaps were rare in the simulations (8.1% of our simulations) and when they happened, they concerned only one or two windows whereas we found 139 windows where HRIs are shared between the four landrace populations. HRIs shared by more populations tend to be more intense. For example, 55% of WE HRIs (λ ≥ 4) colocalize with HRIs of other populations (λ ≥ 4), but this proportion rises to 78% when subsampling WE HRIs with a higher threshold of λ ≥  20. The intensity of recombination in a hot window increases when it is shared by more populations: The median of λ is 10.7, 8.1, and 6.9 when shared by 4, 3, and 2 populations, respectively, and is only 5.9 for population-specific hot windows. This approach to compare HRIs between populations depends on the threshold to claim HRIs and our ability to detect them, which can vary between populations. To make up for these effects, we looked at the recombination intensity (λ) observed in one population around HRIs detected in another population (supplementary file S5, Supplementary Material online). Figure 5*B* presents this average recombination intensity for HRIs detected in each of the four populations. It shows that the local intensity at an HRI position in the other populations is almost twice the background intensity defined as the intensity measured at 100 kb from the HRI center (average λ at HRI positions: 29%; average background λ: 13%). This further shows that HRIs tend to be shared across populations. We evaluated whether this sharing could be explained by assembly errors that would lead to inflated recombination rates in all populations. Indeed, we found that 13% of hot windows shared by the four populations were associated with scaffold boundaries, which is a higher probability than expected by chance (odds ratio = 8.1, *P*-value < 2e−16). In addition, the probability for a hot window to be associated with scaffold boundaries decreased with the level of sharing (odds ratio ranges from 1.3 for population-specific hot windows, 2.2 for hot windows shared by only two populations, 3.7 when shared only by 3 populations and 8.1 for hot windows shared by 4 populations, *P*-values < 0.03). However, these enrichments are not sufficient to explain the patterns of sharing described above. Hence, a significant amount of sharing of HRIs could be due to an underlying partial conservation of recombination hotspots.

**Fig. 6.**
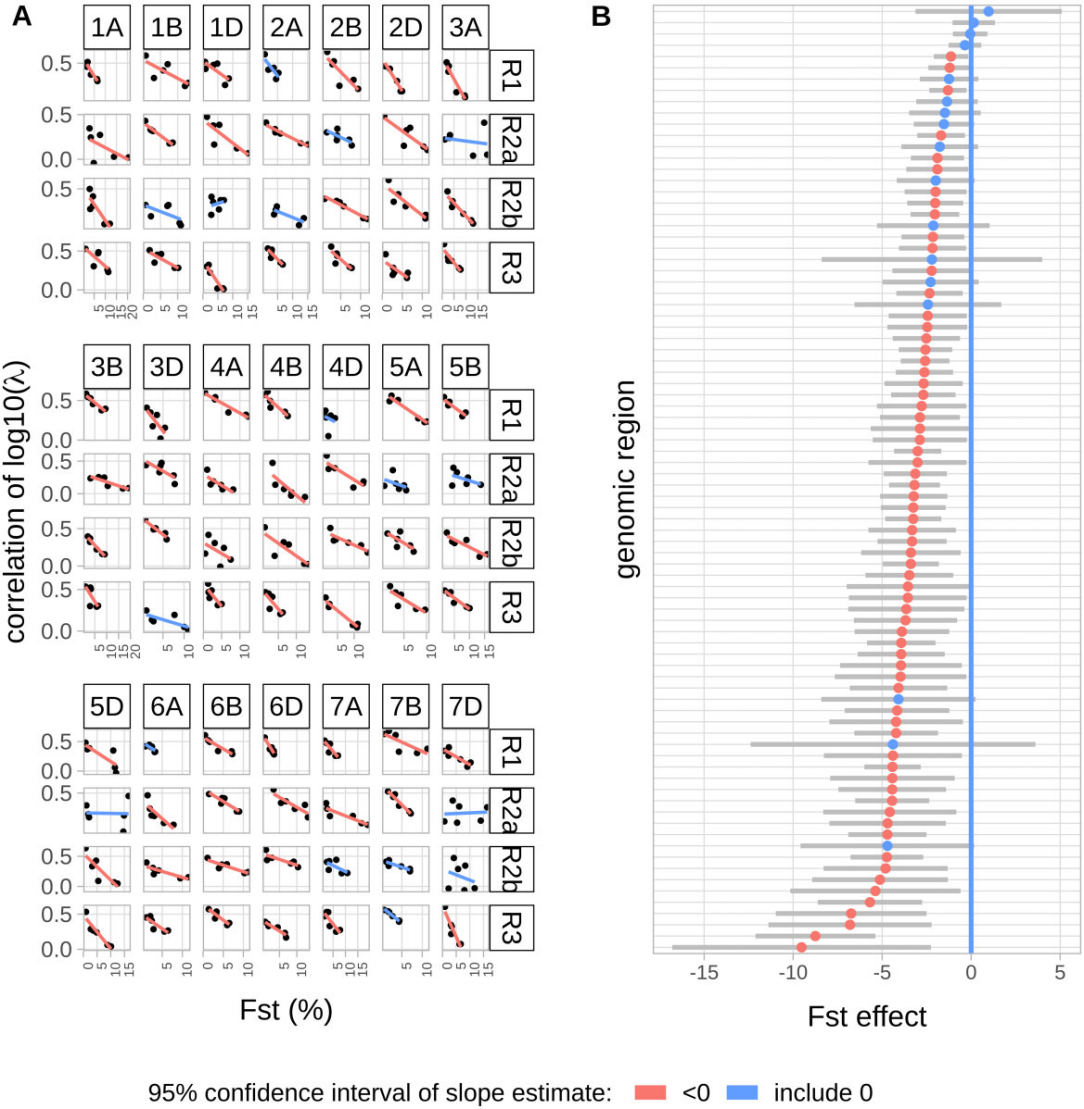
Relationships between correlation of local recombination intensity and *F*_ST_ per genomic region. (*A*) Relationship per genomic region. The slopes values are estimated by linear regression and gives the *F*_ST_ effects on the correlation of recombination profiles. (*B*) Ranked slope estimates (colored points) and their 95% confidence interval (gray bar). Blue color represents slopes with a confidence interval overlapping 0 and red color confidence interval not overlapping 0.

Further examination of the increase in recombination intensity in figure 5*B* reveals that HRI intensities tend to be more similar when populations are more related. For example, around WE HRIs, the recombination intensity increases in all populations, but slightly less in EA which is the most genetically distant population to WE. To study this further, we studied quantitatively the relationships between the similarity in recombination profiles and the genetic divergence of populations. To do so, we fitted a linear regression to estimate the effect of the local differentiation index (*F*_ST_) on the similarity of recombination profiles (measured by their correlation) for all genomic regions (R1, R2a, R2b, and R3) on all chromosomes (1A to 7D) (fig. 6). We found that most *F*_ST_ effects (slopes) were negative, revealing a striking pattern where the similarity in recombination intensity decreases proportionally with genetic divergence: Almost all genomic regions (67 among 84) had a negative slope estimate significantly different from 0 and others genomic regions (15 among 84) had negative but nonsignificant slope estimates different from 0. Note that the similarity in recombination profiles is based on the relative local recombination intensity (parameter λ) that should not be affected by the evolutionary history of populations. *F*_ST_ were calculated from haplotypes rather than single SNPs to avoid an ascertainment effect. But results based on *F*_ST_ calculated from SNPs showed the same pattern (supplementary fig. S10, Supplementary Material online). To further evaluate if the decreasing similarity of recombination patterns could be explained by the varying proportion of shared polymorphisms between population pairs, that is, SNPs ascertainment, we carried out all our analyses on a subset of 100,381 SNPs that are polymorphic in all four populations. We found that the decreasing similarity of recombination intensities with genetic divergence still hold using this common SNP data set (supplementary fig. S11, Supplementary Material online), even if the absolute values of slope estimates were smaller (supplementary fig. S12, Supplementary Material online). We also found no effect of prior distribution parameters in PHASE and sample size on inferences of recombination profile intensity (supplementary protocol S2 and figs. S13−S15, Supplementary Material online). Finally, these results demonstrate that the similarity in recombination profiles of bread wheat populations is strongly negatively associated with their genetic divergence and highlight that recombination landscapes in bread wheat have been evolving during the establishment of the current genetic structure of wheat populations.

To test whether meiotic genes could be associated to the divergence in the recombination profiles of populations, we assessed if pairwise genetic differentiation between populations at these genes (measured by *F*_ST_) was particularly associated to the pairwise correlation of recombination profiles between populations. We computed *F*_ST_ around 54 genes known to be involved in the meiosis process (supplementary protocol S3 and file S6, Supplementary Material online) and fitted a specific regression of the *F*_ST_ around the gene on the genome-wide correlation of recombination profiles (i.e., medians in fig. 4 boxplots, one measure per pair of population, identical for every gene and every genomic region). As the basal level of differentiation depends on the genomic region (fig. 6), we tested whether meiotic genes showed an increased level of differentiation compared with their own genomic region (i.e., a significant negative slope). To control for region-specific effects, meiotic genes were contrasted to “control genes” not involved in meiosis and in the same genomic region. The number of control genes per genomic region ranged from 9 in 7AC to 733 in 5AR3 regions (median number: 223). Overall, meiotic genes did not show a significantly different slope compared with control genes (*P*-value = 0.97). Only *asy4*, located in the 4AR3 genomic region, showed a significantly more negative slope than control genes in its genomic region (False Discovery Rate < 0.01%) (supplementary fig. S16, Supplementary Material online).

## Discussion

### Fine Scale Genome-Wide Recombination Landscape of Bread Wheat

In our study, we estimated LD-based recombination rates for the first time at the whole-genome scale in bread wheat. Previous studies were done at local scale only (Darrier et al. 2017) but suggested that this approach could be applied genome-wide. We used four diverging populations of landraces representative of the four main worldwide genetic groups (Balfourier et al. 2019). For all maps, 80% of the genetic distance was found in 36% (±1%) of the physical distance. This is less concentrated than what was previously observed on single chromosome 3B (80% in less than 20%; Saintenac et al. 2009; Darrier et al. 2017). This discrepancy is likely due, on one hand to the higher SNP density in previous studies on chromosome 3B that allowed to precisely delimit recombination hotspots on this particular chromosome, and on the other hand likely because classical frequentist estimates of recombination rates in biparental maps let most of the genome depleted of recombination. However, and as expected, historical crossovers tend to accumulate in distal sub-telomeric regions of the chromosomes (namely R1 and R3 regions). In most organisms, pairing initiation between homologues occurs in many places along the chromosomes but tends to be favored by a meiosis-specific organization called “bouquet” where telomeres are gathered on the internal nuclear envelope at the Leptotene stage, just before synapsis (Zickler and Kleckner 2015). The bouquet would then facilitate alignment between homologues and pairing would be simultaneously favored through the repair of double-strand breaks including crossovers (Zickler and Kleckner 1998; reviewed in Scherthan 2001 or Harper et al. 2004). In bread wheat, distal crossovers would then be predominant because of the bouquet and be limited in R2a and R2b regions because of interference (Saintenac et al. 2009).

At a fine scale, LD-based maps revealed that 1–2% of intervals of telomeric and pericentromeric regions (depending on the population) exhibited especially high recombination rate (HRIs), suggesting that these intervals overlapped recombination hotspots. The accumulation of crossovers in recombination hotspots was already observed in bread wheat (Saintenac et al. 2011; Darrier et al. 2017) and seems to be a common phenomenon across many species (for a review see Stapley et al. 2017). Recombination hotspots are usually found to be associated with open-chromatin signatures (for a review, see Dluzewska et al. 2018). In previous study in bread wheat, recombination hotspots were found to locate nearby gene promotors and terminators. Our results are consistent with this finding, as most (80%) of our HRIs are located nearby gene features.

### LD-based Recombination Maps Correlate Well with the Biparental Genetic Map

In principle, LD-based recombination maps should be suited to study the similarity of recombination profiles of diverging populations. In our study, they allowed to compare recombination rates of four populations with about twice more SNPs than the densest genetic maps currently available (131 − 170k SNPs in EA and WA, respectively, versus 80k SNPs in Rimbert et al. 2018; 55k markers in Liu et al. 2020; 50k SNPs in Jordan et al. 2018). Moreover, LD-based maps are representative of a whole population and less susceptible to individual specific variation, for example, introgressions which are known to prevent local formation of COs between the introgressed chromatid and the native chromatid. Introgressions from wild relative species are frequent in bread wheat species, representing from 4% to 32% of bread wheat genome (Zhou et al. 2020).

The limitation of LD-based maps relies on the fact that they can be affected by evolutionary patterns, which in turn can hinder their usefulness to study the evolution of recombination rate. Indeed, to the extent that evolutionary forces and past demographic events (bottlenecks, population expansions, hidden structuration) affect LD patterns they can also affect recombination rate estimates (Chan et al. 2012; Dapper and Payseur, 2018). To measure to which extent LD-based recombination rates differ from meiotic ones, we compared LD-based maps with the CsRe meiotic map. This revealed that, genome-wide, the correlation between the two approaches was very high (≥0.7; table 1). Although part of this correlation is explained by the large differences in recombination rate between chromosomal regions (R1, R2a, R2b, R3, and C), our results also indicate a substantial high correlation within each of these regions. The correlation between LD-based and the CsRe genetic map ranged from 0.50 on average in EA, 0.55 in WA and EE and 0.58 in WE at 4 Mb per genomic region considering all populations but only telomeres and pericentromeres (table 1). This value is consistent with correlation values obtained in the literature for other plant species. For example, the correlation between LD-based and meiotic recombination map was found to be 0.3 in rice (Marand et al. 2019), 0.81 in barley (Dreissig et al. 2019), and 0.44–0.55 in Arabidopsis (Choi et al. 2013). Besides, the correlation values we report are likely to be underestimates of the true values. To compute these correlations, we used estimates of recombination rates. Like any statistical estimates they come with measurement errors of the true parameters. Hence the correlation between estimates, providing these errors are independent, are necessarily smaller than the true correlation (Fisher 1915). Apart from this statistical effect, we could also explain some of the differences between LD-based maps and the meiotic map by genomic rearrangements (introgressions on chromosome 7D and 2A in Renan) that are specific to the CsRe population: In these regions, the CsRe recombination profile is not representative of the landraces recombination profiles.

The overall similarity between the meiotic map and LD-based maps shows that LD-based recombination patterns offer a robust representation of the distribution of recombination along the bread wheat genome.

### Robustness of LD-Based Recombination Maps

Despite good concordance with the meiotic map, LD-based recombination maps can still be locally affected by demographic effects, and thus result in bias when interpreting differences or similarities between populations. For example, Kim and Nielsen (2004) and Chan et al. (2012) showed that selective hard-sweeps can produce LD patterns that mimic those of recombination hotspots. Dapper and Payseur (2018) showed that demographic events can decrease the power to detect hotspots leading to an under estimation of the colocalization of LD-based recombination hotspots when using LDhat (Auton and McVean 2007). Here, we used PHASE (Li and Stephens 2003; Crawford et al. 2004), a software to infer recombination rates from LD patterns that implements a quite different methodological approach than LDhat but it is possible that its inference is also affected by such effects. In particular, there were twice many HRIs detected in WE (2,739) and WA (2,743) than in EE (1,968) and EA (1,253), representing a significant variation from 1% of intervals in EA (122,490 SNPs once centromeres removed) to 2% of intervals in WE (161,953 SNPs once centromeres removed) (significant chi-square test, *P*-value < 2.2e−16). Although this varying number of HRIs per population could result from a variation in recombination patterns, it is likely also due to differences in the power to detect HRIs in each population which would be consistent with results from Dapper and Payseur (2018). Indeed, as the proportion of HRIs per population follows the levels of admixture and SNPs density (both higher for WE and WA than for EE and EA), this favors a possible contribution of a different detection power to the variation of HRIs per population. However, we did not observe any atypical LD-based estimate for intervals located nearby genes known to be involved in domestication (e.g., brittle rachis, tenacious glume, homoeologous pairing or nonfree-threshing character) or in recent crop improvement. To further reduce the potential influence of demographic forces on our inference, we performed the comparison between population maps, not on LD-based recombination rates themselves (ρ) but on the relative rate (λ) of recombination in an interval compared with its neighbors in windows of 2 centi-Morgans. Using relative rates should clean our inference from any local effect of demographic forces, especially selection that could tend to be more shared between closely related populations than distant ones.

Results were not much affected by SNPs ascertainment or the method used to calculate the *F*_ST_ index. The decreasing similarity of recombination rates with genetic differentiation still hold when estimating LD-based recombination rates on a population specific SNPs data set or a common SNPs data set. The co-localization of HRIs was also not influenced by the SNPs data set (supplementary fig. S17, Supplementary Material online). The estimation of *F*_ST_ index, using either haplotypic or SNPs alleles, provided also consistent results. Overall, these results strongly support the idea that the decrease of similarity in LD-based recombination profiles is not an artifact of demographic forces or biases due to SNPs ascertainment but that the underlying recombination profile is linked to the divergence of populations.

### Evolution of the Recombination Landscape in Bread Wheat

Our results are consistent with previous reports. Gardiner et al. (2019) showed that closely related bread wheat parental lines lead to RIL populations with more similar crossover profiles. Darrier et al. (2017) compared LD-based recombination profiles of a European and an Asian population, the two main ancestral bread wheat genetic pools, on two scaffolds of 1.2 and 2.5 Mb on chromosome 3B. They found that LD-based recombination profiles are broadly conserved, but highlighted that hot intervals in LD-based recombination profiles were not necessarily shared between these two European and Asian populations. Similar results were observed in other plant species such as rice (*Oryza sativa*; Marand et al. 2019) and cocoa-tree (*Theobroma cacao*; Schwarzkopf et al. 2020). Other plant studies hint at a possible decreasing similarity of fine-scale recombination profiles over evolutionary time measured by *F*_ST_, such as maize (*Zea mais*, Rodgers-Melnick et al. 2015), poplar (*Populus* species, Wang et al. 2014, 2016), cotton (*Gosypium hirsutum*, Shen et al. 2019), and barley (*Hordeum vulgare*, Dreissig et al. 2019).

Several hypotheses can be formulated to explain the differences in recombination profiles between populations. First, this can be due to environmental effects. This is the case in barley, where recombination rates vary along the genome and are affected by environmental conditions as well as by domestication (Dreissig et al. 2019). For example, high temperatures are known to affect meiosis and above 35 °C, this may lead to complete failure and severe sterility (Loidl 1989; Higgins et al. 2012). Interestingly, within a range of 22 − 30 °C, highest temperatures may modify the recombination profile. In barley, it was shown that at 30 °C, distal recombination events are reduced whereas interstitial events became more frequent revealing thus a slight shift and a modification of the global recombination profile (Higgins et al. 2012). However, in our case, this hypothesis is not the most likely as we were using populations from the same hemisphere and latitudes, with landraces from different countries. Environment is thus certainly very different between all the origins of our landraces and temperature should vary a lot in each location and is not stable enough to affect durably and maintain a different recombination profile between the four populations. Moreover, it was recently shown that increased temperature up to 28 °C for 3 weeks during wheat meiosis has only a limited impact on recombination distribution (Coulton et al. 2020).

Secondly, differences in recombination profiles can be explained by differences in the chromatin accessibility landscape during meiosis between populations. Many studies showed that chromatin status is the main feature that drives recombination in plants. DNA is partitioned in blocks of heterochromatin and euchromatin which are dispersed along the chromosomes. In bread wheat, heterochromatin preferentially locates in pericentromeric regions whereas euchromatin-rich DNA is more frequent in distal subtelomeric regions of the chromosomes (IWGSC 2018). In *Arabidopsis*, it was shown that crossovers are enriched in euchromatin and mainly occur close to gene promoters and terminators (Choi et al. 2013; Drouaud et al. 2013). Meiotic recombination profile in this species is also shaped by H2A.Z nucleosome occupancy, DNA methylation or epigenetic marks such as Histone 3 Lysine 9 di-methylation (H3K9me2; Choi et al. 2013; Underwood et al. 2018). This led to our second hypothesis that chromatin status has evolved between our four populations, rather than an evolution of the recombination determinism itself. Divergence in chromatin status could be explained by genetic drift on one hand or by selection pressure around different genomic regions depending on geographical area on the other hand. This selection pressure could therefore contribute to the deposition of histone landmarks to regulate gene activity such as H3K4me3, H3K9ac, and H3K27ac that are associated with transcriptional activation (Roth et al. 2001; Howe et al. 2017) or on the contrary H3K27me3 and H3K9me3 associated with transcriptional suppression (Saksouk et al. 2015). Interestingly, in some mammals, recombination is directed by the zinc-finger protein PRDM9 that possesses a set domain that catalyzes the trimethylation of lysine 4 of H3 to produce H3K4me3 (for review see Grey et al. 2017). Similar mechanisms involving histone 3 modifications such as methylation or acetylation that could affect recombination profile afterward are thus likely in plants. We tested differentiation of 54 meiotic genes along evolution of recombination profile. In average, these 54 meiotic genes did not show a higher or lower differentiation level than control genes of their own genomic region. Only ASY4 located in 4AR3 genomic region, showed a significant higher level of differentiation than control genes. In *Arabidopsis*, the *asy4* protein is involved in the formation of the axis between the two sister chromatids (Chambon et al. 2018). Mutation of *Atasy4* significantly reduces the number of crossovers and induces a shift toward the distal parts of the chromosomes. This could explain why we found this gene associated with a difference in recombination rates between populations.

Another factor that may explain the difference of recombination patterns between the populations could be the natural introgression of alien DNA fragments from wheat relatives during the evolution process. Introgressions from wild-species have been widely used and more than 50 alien germplasms have been used to improve wheat varieties (Wulff and Moscou 2014). For example, Renan possesses two introgressed fragments from *Aegilops ventricosa* conferring resistance to leaf, yellow, and stem rusts (*Lr37*/*Yr17*/*Sr38*) on chromosome 2A (2A/2N translocation) and to eye-spot (*Pch1*) on chromosome 7D (7D/7Dv translocation; Maia 1967; Helguera et al. 2003). These introgressions repress recombination (Worland et al. 1988) and this resulted in a poor correlation between CsRe genetic map and our LD-based maps for genomic region 7DR3 in our analysis. It was recently shown that natural or artificial introgressions of wheat wild-relatives DNA contributed to up to 710 and 1580 Mb in wheat landraces and varieties, respectively (Cheng et al. 2019), and represent from 4% to 32% of bread wheat varieties genome (Zhou et al. 2020). A similar analysis used exome capture to evaluate introgression in 890 hexaploid and tetraploid wheats (He et al. 2019). The results also suggest that introgressions of DNA fragments from wheat relatives contributed significantly to improve the diversity of current wheat cultivars. Because natural introgressions are frequent in wheat landraces and because they contribute to modify the recombination profile, we could hypothesize that these introgressions are different in our four collections, which would result in different recombination profiles as well. Only an extensive sequencing of our accessions would allow to bring the answer.

## Conclusion

This study demonstrates the evolution of the recombination profile at a genome-wide scale in closely related wheat populations with increasing genetic divergence. Based on recombination landscapes robust to demographic events, the comparison of the four landrace populations revealed a clear signal of a decreasing similarity between fine-scale recombination landscapes with increasing genetic divergence. Specifically, we found 1) that HRIs were more shared between closely related populations, 2) recombination intensities at HRIs detected in one population decreased in the other populations with their genetic divergence, and 3) the correlation of recombination landscapes between pairs of population decreases with their local genetic differentiation as measured by *F*_ST_. Our results, interpreted in the light of previous findings in bread wheat and other species, clearly shows that recombination landscapes in wheat change with genetic divergence between populations. Being based on closely related populations that recently diverged (no more than 10,000 YA), this study further shows that this divergence can be quite fast. Reasons for this divergence remain to be found but our results can hint at some possibilities. Further analyses are needed to settle this question, which should greatly help developing original approaches useful for wheat improvement and breeding.

## Materials and Methods

### Plant Material

A collection of 632 bread wheat landraces (Balfourier et al. 2019) was genotyped on the TaBW410k SNPs including 280k SNPs from the Axiom Affymetrix TaBW280k SNPs array (Rimbert et al. 2018). Besides, a population of 406 F6 RILs derived from the cross between the Asian variety Chinese Spring and the European variety Renan (CsRe), were also genotyped on the TaBW280k SNPs array (Rimbert et al. 2018). After quality filtering including control of missing data rate (10% maximum), heterozygosity rate (5% maximum), excluding off-target variants, 578 landraces genotyped with 200,062 SNPs were kept for the population-based analysis and 79,564 polymorphic SNPs were successfully mapped on the CsRe population.

The physical positions of SNPs on the 21 bread wheat chromosomes were determined using Basic Local Alignment Search Tool (Blast; Altschul et al. 1990) of context sequences on the International Wheat Genome Sequencing Consortium RefSeq v1.0 genome assembly (IWGSC 2018). Position of high confidence genes, exon, 5′-UTR and 3′-UTR were extracted from RefSeq V1.0 annotation.

### Robust Estimation of the Meiotic Recombination Profile

Due to the relatively low number of meiosis sampled in the CsRe data, a Bayesian model inspired from Petit et al. (2017) was used to obtain robust estimates of recombination rates. We modelled the probability distribution of the recombination rates observed in RILs ($C_{i}$) given the number of observed recombination events ($y_{i}$) as:

$$P(C_{i}\;|\;y_{i})=\frac{P(y_{i}\;|\;C_{i})\;P(C_{i})}{P(y_{i})}$$

.

The likelihood $P(y_{i}\;|\;C_{i})$ is modelled as a Poisson distribution, its parameter being the expected number of recombination events in an interval and computed as: $E(y_{i})=C_{i}\times L_{i}\times M$, where $L_{i}$ is the physical size (in megabases, Mb) of the interval and M the total number of RILs. Thus, the likelihood of the recombination rate $C_{i}$ is:

$$P(y_{i}|C_{i})∼\mathrm{Poisson}(C_{i}\times L_{i}\times M)$$

.

To specify a prior distribution of $P(C_{i})$, we considered that the wheat recombination landscape varies widely along a chromosome. According to the nomenclature of Choulet et al. 2014, each of the wheat chromosomes can be segmented into five chromosomic regions associated with different global recombination rates and genomic content: Two highly recombining telomeric regions (R1 and R3), two low-recombining pericentromeric regions (R2a and R2b) and one centromeric region (C) where recombination is almost completely suppressed. The small arm of each chromosome is composed of R1 and R2a whereas the long arm is composed of R2b and R3. The physical size of these regions ranges between 10 Mb for the smallest telomere and 321 Mb for the largest pericentromere (supplementary file S7, Supplementary Material online). To account for the specific range of recombination rate variation in each region in our model, the prior distribution of the recombination rates in each of these regions was a specific Gamma distribution:

$$P\left(C_{i(r)}\right)∼Г(\alpha_{r},\beta_{r}),$$

where r denotes the region, α_r_/β_r_ gives the mean of the Gamma distribution and $\alpha_{r}/\beta_{r}^{2}$ gives the variance. The Gamma distribution being a conjugate prior to the Poisson distribution, the posterior distribution of *C*_i_ is also a Gamma distribution:

$$P(C_{i}|y_{i})∼Г(y_{i}+\alpha_{r};M\;L_{i}+\beta_{r})$$

.

The posterior mean of *C*_i_ (in M/Mb) is then:

$$C_{i(r)}^{\mathrm{bay}}=\frac{y_{i}+\alpha_{r}}{M\;L_{i}+\beta_{r}}$$

.

The parameters $\alpha_{r}$ and $\beta_{r}$ of the prior Gamma distribution were set using an empirical Bayes approach (i.e., estimating prior distribution directly from data), independently for each of the five r regions (supplementary fig. S18, Supplementary Material online). A Gamma distribution was fitted (R MASS package, Venables and Ripley 2002) over the distribution of frequentist recombination rates observed in RILs. This latter was computed as

$$C_{i}^{\mathrm{freq}}=\frac{y_{i}}{M\;L_{i}}$$

.

Note that null recombination rates were replaced by the lowest non-null estimate of recombination rates of the region to allow fitting the Gamma distribution. We derived the meiotic recombination rates from the RILs recombination rates using the Haldane and Waddington formula (Haldane and Waddington 1931) and the Morgan mapping function (cM = frequency of recombinants × 100). Indeed, the size of intervals (median = 5 kb) were small enough to consider that interference is very strong within and thus one recombination in one individual result from only one crossover (and not from coincidence of several crossovers). We thus obtained the Bayesian meiotic recombination rate $c_{\mathrm{CsRe}\;i}^{\mathrm{bay}}$ (cM/Mb).

#### Considering Uncertainty in Crossover Locations

For estimation of recombination rates, it was necessary to count the number of recombinants in CsRe intervals (*y*_i_). Missing data on genomic segments with no parental allele switch at segment extremities were imputed. A crossover was counted at each parental allele switch, yielding 26,239 crossovers. Due to the presence of missing data in RILs genotypes, a number of switches did not occur between pairs of immediately adjacent markers. In such cases, the crossover cannot be assigned with certainty to a single interval of two successive SNPs. For example, an RIL genotype AA/–/BB identifies a switch between the first and third marker but cannot discriminate a recombination in the first versus the second interval. In such cases, we accounted for the uncertainty in crossover location following the sampling procedure of Petit et al. (2017). Briefly, each crossover is overlapped by a set of one or more intervals. A sampling procedure assigned each crossover to a particular interval with a probability computed as the size of the interval divided by the size of the crossover area (physical distance between the two closest SNPs showing different parental alleles). Repeating 1,000 times the sampling procedure yields 1,000 estimates of $y_{i}$ per interval, which can then be converted into recombination rates and averaged.

### LD-Based Recombination Profiles of Four Diverging Populations of Landraces from Patterns of LD

#### Identification of Four Diverging Populations of Landraces Representative of Bread Wheat Worldwide Diversity

We defined four populations from a data set of 632 landraces representative of worldwide genetic diversity of bread wheat and previously described in Balfourier et al. (2019). The constitution of populations followed a three steps procedure that we briefly described (more details in supplementary protocol S4 and figs. S1 and S19, Supplementary Material online):

- From Balfourier et al. (2019)*K* = 4 admixture analysis of the 632 landraces, we kept only 534 low admixed landraces to maximize differentiation between future four populations.
- The 534 landraces were gathered into 4 groups using a hierarchical clustering on the pairwise distance matrix estimated in Balfourier et al. (2019). The four populations were named as West Europe (WE), East Europe (EE), West Asia (WA), and East Asia (EA) from the geographical origin of their members. The pairwise matrix distance gave the proportion of mismatched haplotypic alleles along the genome, computed using 8,741 haplotypic blocks containing up to 20 alleles per block (figure 1 of Balfourier et al. [2019]).
- We discarded closely related individuals within each population to avoid over representing family specific recombination events. Pairs of individuals exhibiting a very low genetic difference were discarded, keeping a total of 371 landraces.

#### Evolutionary Distance between Populations Measured by F_ST_

Pairwise differentiation indexes (*F*_ST_) of the four populations were computed within each genomic region (chromosomal region within a chromosome, e.g., 1AR1) using alleles of 8,741 haplotypic blocks (Weir and Cockerham distance, R hierfstat package, function pairwise. WCfst, Goudet and Jombart 2015) or SNPs (Reynolds distance, HAPFLK software, Bonhomme et al. 2010; Fariello et al. 2013) (supplementary file S8, Supplementary Material online).

#### Inferences of LD-Based Recombination Rates from LD Patterns

LD-based recombination rates were estimated using PHASE software V2.1.1 (Li and Stephens 2003; Crawford et al. 2004; Stephens and Scheet 2005). PHASE inputs were successive windows of SNPs along the genome, constituted of one central part and two flanking parts overlapping the previous and the next windows to avoid border effect in PHASE inferences. Central and flanking parts spanned on average 1 cM and 0.5 cM, respectively, based on the CsRe genetic map (supplementary protocol S5, Supplementary Material online). PHASE was run for each window with default options, except for two parameters of the Markov Chain Monte Carlo (MCMC), following recommendations of the documentation on estimating recombination rates. The number of sampling iterations was increased to obtain larger posterior samples (option -X10) and the algorithm was run ten times independently (option –x10) to better explore combinations of parameters and keep the run with the best goodness of fit. The sampling stage of the MCMC yielded 1,000 samples of the posterior distribution of:

- The background recombination rate of the window w: $\rho_{w}$
- The ratio $\lambda_{i}$ between the background recombination rate of the window $\rho_{w}$ and the LD - based recombination rate in each interval i of two successive SNPs $\rho_{i}$ so that $\rho_{i}=\lambda_{i}*\rho_{w(i)}$ where *w*(*i*) identifies the window which interval *i* belongs to. The parameter $\lambda_{i}$ can be seen as a measure of local recombination intensity compared with genomic background (inflation or deflation).

PHASE samples jointly $\rho_{w}$ and $\lambda_{i}$ in their posterior distribution at each iteration, so their product yields 1,000 samples of the posterior distribution of LD-based recombination rate $\rho_{i}$ (/kb) (supplementary file S9, Supplementary Material online). We assessed the sensibility of PHASE results to prior distribution parameters and population sample size and we found that our inference was robust to modifications of the prior distribution or the down-sampling of the WE population (supplementary protocol S2, Supplementary Material online).

#### Correlation of LD-Based Recombination Profiles

To compare LD-based recombination profiles, it was necessary to obtain a common set of intervals across the four populations (WE, EE, WA, EA), as polymorphic SNPs sets were different. We defined smaller intervals formed of successive markers that were polymorphic in at least one population (supplementary fig. S20, Supplementary Material online). For each population, the recombination estimates in smaller intervals were considered to be the same as the estimates belonging to population specific intervals overlapping them, assuming that recombination rates are constant within intervals. We removed intervals not overlapped by all populations on chromosome extremities. This process yielded a complete factorial data set of 194,409 intervals with no missing data and a set of 1,000 values sampled from the posterior distribution for each parameter $\rho_{pi}$ and $\lambda_{pi}$ per interval *i* and per population *p*. The similarity between LD-based recombination profiles was measured by correlating the$\log_{10}$ of median of $\lambda_{pi}$ (noted log10λpi) of all intervals between different populations. The median of posterior distribution of $\lambda_{pi}$ was chosen as it is robust to outliers in the posterior distribution, as recommended (Li and Stephens 2003) and using the log scale is natural when comparing intensities across groups. To obtain correlation coefficients, a linear model including a full unstructured variance−covariance matrix was fitted on log10λpi, so that each population had its own range of variation of local recombination intensity and each pair of population has a specific covariance parameter:

Ypi=log10λpi

$$Y_{pi}=\mu +E_{pi}$$

$\vec{E}$ ∼MVN(0,$I_{n}⊗\Sigma_{4*4})$, where $\Sigma_{4*4}$ is a variance−covariance matrix from which we extract correlation coefficients.

The model was applied independently to each genomic region (from 1AR1 to 7DR3, except centromeric regions, results in supplementary file S10, Supplementary Material online). The total number of intervals n per genomic region ranged from 154 to 8,131. The differences of recombination intensity profiles across the four populations of landraces were assessed by model comparison. We compared the Bayesian Information Criterion (BIC) of a model with a full variance−covariance matrix with a simpler model with a variance−covariance matrix including only one correlation parameter for all pairs of populations. The complex model was deemed to be a better model if its BIC was inferior to the BIC of the simpler model. The models were fitted with ASReml-R V3 (Butler et al. 2009).

### Colocalization of HRIs between Populations

Intervals with a LD-based recombination rate exceeding four-times or more the background recombination rate (λ ≥ 4) figuring as outliers in λ distribution (supplementary fig. S21 and file S11, Supplementary Material online), were defined as HRIs and adjacent HRIs within a population were merged. Due to strong heterogeneity of HRI’s size, we discarded too small or too wide HRIs (supplementary protocol S6, Supplementary Material online). For each HRI in each population, the overlapping HRIs in other populations were recorded. A set of HRIs intervals was considered as co-localizing in two, three or four populations if all HRIs overlapped each other (i.e., they formed a clique in network terminology). Note that this implies that a wide HRI can potentially be involved in more than one clique. For each group of colocalizing HRIs (each clique), we defined a hot window as the smallest common overlapped area (supplementary file S12, Supplementary Material online). Population specific HRIs, that is, HRIs which did not overlap any other HRIs, also formed hot windows whose frontiers were defined by the upper and lower limit of HRIs. Each hot window thus included HRIs of one, two, three or four populations. The proportion of HRIs shared by two populations or more (e.g., WE and EE) was computed as the number of hot windows including HRIs of each population (hot windows including both WE’s HR and EE’s HR) divided by total number of hot windows (including either WE, EE, WA or EA’s HRIs) (supplementary fig. S22, Supplementary Material online). Dividing by the total number of hot windows is more convenient to compare the proportion of HRIs population-specific, or shared by two, three or four populations.

To test for the hypothesis that the observed proportion of HRIs shared by populations is due to chance, an empirical range of plausible values of co-localization due to chance was estimated by simulation. In 1,000 simulations, each HRI of each population was assigned to a random interval within the genomic region it belongs (1AR1 to 7DR3) and the proportion of shared hot windows was computed (supplementary file S13, Supplementary Material online).

### Comparison of the LD-Based Recombination Rates and the CsRe Meiotic Recombination Rates

The comparison between meiotic (CsRe) and LD-based recombination rates were done on windows of 4 Mb (∼1 cM on average, wide enough to accurately estimate intrinsic recombination rate) along the genome. Meiotic recombination rates were estimated using the Bayesian model described above, the attribution of crossover to windows being done using the (Petit et al. 2017) approach (see above). To compute the LD-based recombination rate in 4 Mb windows, the total LD-based genetic distance per window of 4 Mb was divided by the total physical distance and averaged over the 1,000 samples of the posterior distribution:

$$\rho_{pw_{4Mb}}=\frac{1}{1,000}\sum_{j=1}^{1,000}\frac{\sum_{i\epsilon w_{4Mb}}^{}\left(\rho_{\mathrm{pij}}*L_{i}\right)}{\sum_{i\epsilon w_{4Mb}}^{}L_{i}}$$

with *i* the interval and *j* one posterior distribution value among 1,000 (supplementary file S14, Supplementary Material online).

## Supplementary Material

Supplementary data are available at *Genome Biology and Evolution* online.

## Supplementary Material

evab152_Supplementary_Data

## Data Availability

Genotyping data set of landraces on TaBW410k (Kitt et al. 2021) after quality control is available as a Zeonodo repository (https://doi.org/10.5281/zenodo.4518374). Genotyping data set of RILs (Kitt et al. 2018) is available as a Zenodo repository (https://doi.org/10.5281/zenodo.4486612). Supplementary figures and protocols can be found in the main supplementary file. Supplementary data including PHASE outputs and population-specific meiotic recombination maps, are available in a Zenodo repository (https://doi.org/10.5281/zenodo.4486586). Computer code and scripts needed to reproduce all results are available on Github (https://github.com/aldanguy/Bread_wheat_recombination).
