# Supplementary material for: Evolution of Recombination Landscapes in Diverging Populations of Bread Wheat
Source: Genome Biol Evol. 2021 Jun 29;13(8):evab152. doi: 10.1093/gbe/evab152 (PMC8350361; doi:10.1093/gbe/evab152)
Supplement: evab152_Supplementary_Data [file evab152_supplementary_data.zip › Danguy_et_al_supplementary.pdf]

Supplementary: Evolution of recombination landscapes in diverging populations of bread wheat.

Alice Danguy des Déserts [1], Sophie Bouchet [1], Pierre Sourdille [1*], Bertrand Servin [2*]

[1] INRAE-Université Clermont-Auvergne, UMR1095, Génétique Diversité Ecophysiologie des Céréales, 5 chemin de Beaulieu, 63000, Clermont-Ferrand, France

[2] INRAE, UMR 1388, Génétique, Physiologie et Systèmes d'Elevages, F-31326, Castanet-Tolosan, France

* Corresponding authors

- Bertrand Servin, PhD, email: bertrand.servin@inrae.fr
- Pierre Sourdille, PhD, email: pierre.sourdille@inrae.fr

Supplementary protocols

Protocol S1: Population-specific meiotic recombination profiles from LD-based recombination profiles. Assuming the LD-based recombination rate ρ is proportional to the meiotic recombination rate c (*e.g.* in a Wright Fisher model, $\rho = 4N_ec$, where N_e is the effective diploid size of the population), LD-based recombination profiles can be scaled based on the CsRe Bayesian meiotic recombination map: the ratio between the average CsRe Bayesian meiotic recombination rate and average LD-based recombination rate of a genomic region yields an estimate of the coefficient of proportionality. This scaling implies two hypotheses: 1) the average recombination rate in a genomic region is the same across the five populations CsRe, WE, EE, WA and EA. For example, CsRe has an average Bayesian meiotic recombination rate of 0.8 cM/Mb in 3BR1, so this would be the average recombination rate of this genomic region in the four populations of landraces. 2) There is no variation in the proportionality coefficient within a genomic region. Landraces and CsRe meiotic genetic maps can be found in supplementary file S4.

Protocol S2: Sensibility of LD-based recombination rate intensity profile (λ) to population sample size and prior parameter distribution.

S2A. Sensibility to population sample size: To downsample WE population (127 landraces) and reach EE sample size (70 landraces), we build a hierarchical clustering of the 127 WE landraces using a simple-matching distance matrix (Balfourier & al. 2019) and Ward distance. We cut the hierarchical clustering into 70 groups and randomly sampled one landrace per group (supplementary Figure S13). This yielded a new population, named WEds, made of 70 landraces. This procedure allows WEds population to mimic at best, WE genetic composition. We then estimated WEds LD-based recombination profile with PHASE software using the WE SNPs dataset and the same PHASE settings as described in the article. Thus, for each interval of two successive SNPs, we had one posterior distribution of λ for WE and one posterior distribution of λ for WEds. For each interval and each population, we took the median of these distributions as estimates of local recombination intensity (supplementary Figure S14, left). To compare such correlation with the variability of PHASE inferences due to random start of the Markov Chain Monte Carlo (MCMC, "run effect"), we re-estimated WE LD-based recombination profile (same landraces set, same SNPs set) (supplementary Figure S14, right).

S2B. Sensibility of LD-based recombination rate intensity (λ) to PHASE software prior distribution parameters. According documentation for PHASE v2.1 (Stephens et al. 2004), several prior distribution parameters can be modified by user: "*The form for the prior on the background recombination parameter, $\underline{\rho}$, is that log(ρ) is normal with mean log(μ) and standard deviation σ (truncated so that $\underline{\rho}$ is forced to lie between 10^{-8} and 10^{3}). The value of σ is 0.5*log(f), where f is chosen so that you would typically (95% of the time) expect $\underline{\rho}$ to be within a factor f of μ.*"

We estimated LD-based recombination rates of WE population using either the default prior for the background recombination rate ρ_w (μ = 0.0004 and f = 1e6), a prior centered on high values for ρ_w (μ = 1 and f = 1e6) and a prior centered on low values for ρ_w (μ = 1e-7 and f = 1e6). For each prior distribution and each interval of two successive SNPs, we extracted the median of the posterior distribution of λ_i and of $\rho_i = \lambda_i * \rho_{w(i)}$.

Protocol S3: Differentiation at meiosis genes. From a list of 296 genes known to be involved in the meiosis process in bread wheat (Pierre Sourdille, personal communication), we kept 54 genes overlapped by at least 5 SNPs of the common SNPs dataset (including a 10 kilobases extra margin on each side of the gene). We also extracted a subset of 9,826 genes from RefSeq V1.0 annotation belonging to the same genomic region than meiosis genes and

respecting similar SNPs coverage rules than meiosis genes. These genes are supposed to be neutral toward the meiosis process and will be considered as control genes to measure the background differentiation level across populations of landraces. F_{ST} values for each gene were computed with HAPFLK software using their overlapping SNPs. The differentiation level of each gene is given by the linear relationship (slope) between the F_{ST} and correlation of genome-wide recombination profile.

In a first place, to test whether meiosis genes were in average more or less differentiated than control genes of their genomic region, we used the linear model:

$$Y_{pg(rc)} = \mu + \alpha_r + \beta_c + (\omega + \delta_r + \gamma_c) * X_p + E_{pg(rc)} \text{ with } E_{pg(rc)} \sim N(0, \sigma^2) \qquad (1)$$

where p is the pair of populations (6 levels, WE-EE; WE-WA; WE-EA; EE-WA; EE-EA; WA-EA), g is the gene (9,880 levels), r is the genomic region (32 levels) and c is the gene category (2 levels, control or meiosis). $Y_{pg(rc)}$ is the pairwise F_{ST} value per pair of population p, for gene g belonging to genomic region r and gene category c. The term $(\mu + \alpha_r + \beta_c)$ gives the average pairwise F_{ST} per genomic region and gene category. The co-variables X_p are the median of correlation of log10(λ) across all genomic regions except centromeres (one measure per pair of population p, identical for every gene and every genomic region). The term ω is the linear relationship between F_{ST} and correlation, *i.e.* what we call the differentiation level. The term δ_r indicates that differentiation levels might vary across genomic regions. The term γ_c and its significance level (evaluated by t-test) indicates whether meiosis genes were in average more or less differentiated than control genes of their genomic region.

For each gene, we also estimated the deviation from the background differentiation level by adjusting a linear model for each genomic region independently:

$$Y_{pg} = \mu + \alpha_g + (\omega + \gamma_g) * X_{pg} + E_{pg} \text{ with } E_{pg)} \sim N(0, \sigma^2) \qquad (2)$$

The indices p gives the pair of populations (6 levels), the term g gives the gene name (from 9 levels in 7AC to 740 levels in 5AR3). The variables Y_{pg} and X_{pg} still represent pairwise F_{ST} and correlations respectively. The term $\mu + \alpha_g + \beta_c$ gives the average pairwise F_{ST} in the genomic region for the gene g; the term ω is the average relationship between F_{ST} and correlation of recombination profile and the term γ_g is the gene-specific deviation to average relationship between F_{ST} and similarity of recombination profile. Note that we set sum-to-0 constraints when estimating the γ_g terms.

The estimates of deviations and their standard errors were used to compute False Discoveries Rates using the ashr R package (Stephens et al. 2020). Genes showing a FDR lower than 0.01% were considered as significantly more differentiated than the genomic background.

Protocol S4: Identification of four diverging populations of landraces. Balfourier et al. (2019) analysed the genetic structure of the 632 landraces dataset and could pinpoint four main groups corresponding to the geographic origins of lines. Despite this structuration, the general pattern of differentiation in these data is somewhat continuous, a lot of individuals exhibiting admixed origins. Here, we subsampled the dataset in order to constitute populations of individuals that were both homogeneous within groups and clearly differentiated between groups. This was achieved in three steps. i) From the Balfourier et al. (2019) admixture analysis with K=4 groups, landraces exhibiting an admixture coefficient smaller than 50% of their dominant group were removed, yielding 534 low admixed landraces (supplementary Figure S1). ii) These 534 landraces were grouped into four populations by hierarchical clustering on the pairwise distance matrix estimated in Balfourier & al (2019) and using the Ward's grouping criterion. The four populations were named as West Europe (WE), East Europe (EE), West Asia (WA) and East Asia (EA) from the geographical origin of their members. The genetic

difference (distance) between two landraces was the proportion of mismatched haplotypic alleles along the genome, computed using 8,741 haplotypic blocks containing up to 20 alleles per block (Figure 1 of Balfourier et al. 2019). iii) The last step aimed at discarding closely related individuals within each population to avoid over representing family specific recombination events. To spot closely related landraces, outliers were called from the distribution of genetic distance as follows. Within each population, we fitted a Normal distribution to the observed distribution of distances using robust estimators for the mean and variance (R MASS package, function rlm, (Venables and Ripley 2002). Based on this distribution we tested whether the distance of a particular pair of individuals was consistent with this normal distribution (note that the test is one sided as we only tested for outlying low values). We corrected for multiple testing by applying a False Discovery Rate correction based on the normal p-values (R qvalue package, function qvalue, Storey et al. 2015). Pairs of landraces showing a q-value lower than 0.001 were considered as related. An iterative algorithm was designed to suppress related landraces: at each step, the algorithm first computes the number of relatives per landrace, and then removes the landrace exhibiting the highest number of relationships. The algorithm stops when no related pair remains in the set of individuals. Relationships of individuals within populations are represented in supplementary Figure S19.

Protocol S5: Definition of PHASE windows. PHASE windows were defined of successive SNPs spanning 2 cM (centre 1 cM and borders 0.5 cM) according to the CsRe Bayesian genetic map. The genetic positions of SNPs that were not polymorphic in CsRe were estimated based on their physical position and the physical and genetic positions of mapped flanking markers. A linear interpolation was fitted on the physical and the genetic positions of the 79,564 SNPs of the CsRe Bayesian map and used to predict genetic position of the other SNPs (R base package, function approxfun). Estimates of first SNPs in R1 and last SNPs in R3 in many chromosomes were often not possible, because no CsRe marker was mapped that far in chromosome extremities. The genetic positions of those extreme SNPs were estimated using the average recombination rate of CsRe intervals situated in R1 region (respectively situated in the R3 region) and their physical distance from the closest mapped marker. The final interpolated genetic map was set to start at 0 cM for each chromosome (example in supplementary Figure S23).

The total number of SNPs within PHASE windows had to be controlled: a minimum of 50 SNPs to ensure reliability of inferences, no more than 160 SNPs to reduce computational time. This required adjusting the number of SNPs in central and flanking parts for some windows (in supplementary Figure S24).

Protocol S6: Remove of 20% HRIs based on their physical length. We discarded the 10% smallest and 10% largest HRIs, assuming that very small intervals exhibiting high recombination rates likely reflect problems in genome assembly or that very wide HRIs do not allow to properly study colocalization of HRIs. After filtering, the size of the widest HRIs was around 100-times higher than the size of the smallest one (around 100k-times higher if filtering on HRIs size is not performed, supplementary Figures S25, S26).

Supplementary Figures

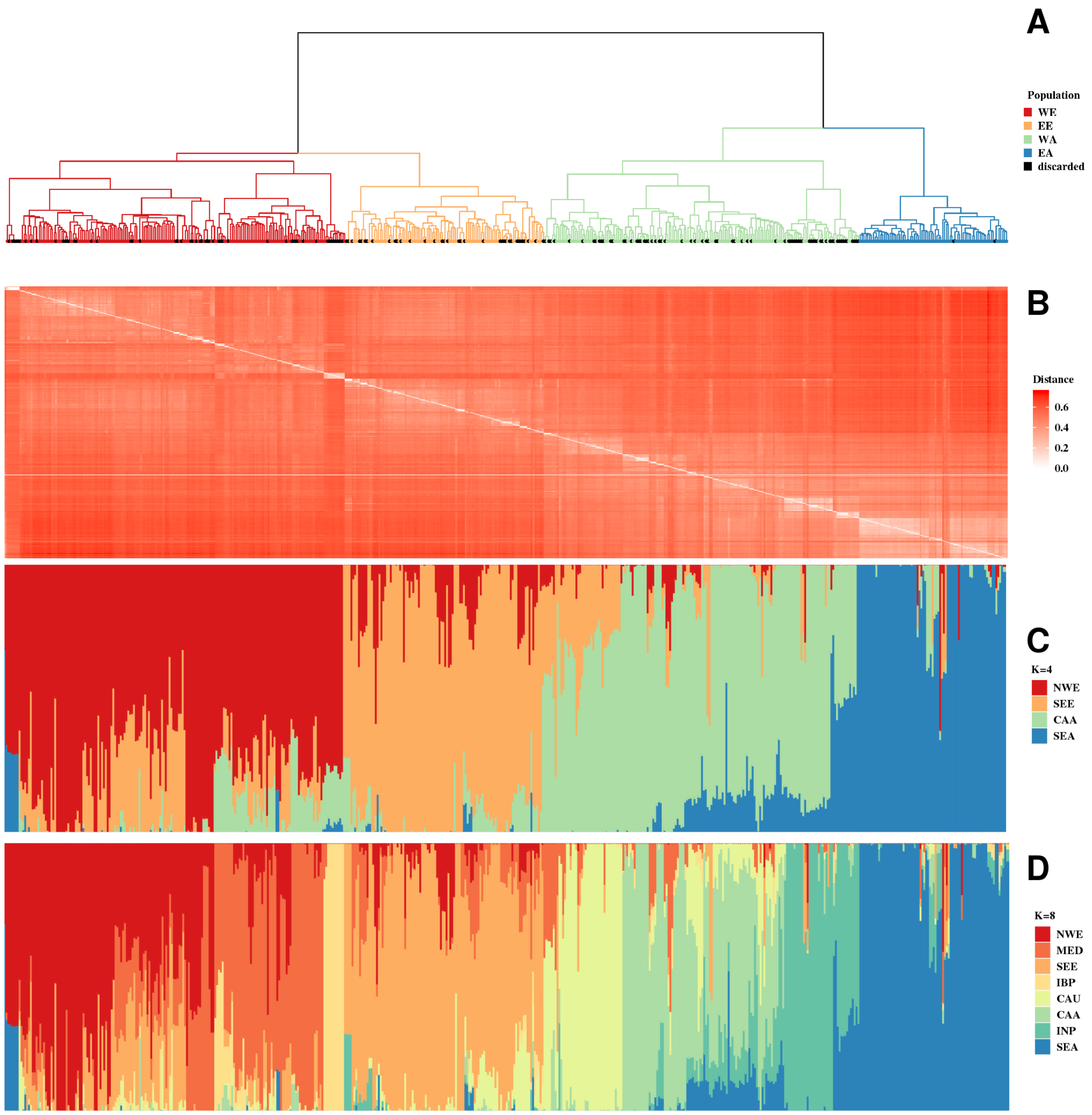

Figure S1: Bread wheat landraces genetic diversity and structuration at K=4 and K=8. A Hierarchical clustering to identify four bread wheat landrace populations. WA = West Asian population; EA = East Asian; WE = West European; EE = East European; discarded = too closely related landraces removed from the analysis. **B** Pairwise simple matching distance matrix from Balfourier et al. 2019 **C and D** STRUCTURE results for K=4 and K=8 groups, from Balfourier et al. (2019). NWE = North West European, MED = Mediterranean, SEE= South

East European, IBP = Iberian Peninsula, CAU = Caucasian, CAA = Central Asian and African group; INP = Indian Peninsula, SEA = South Est Asian.

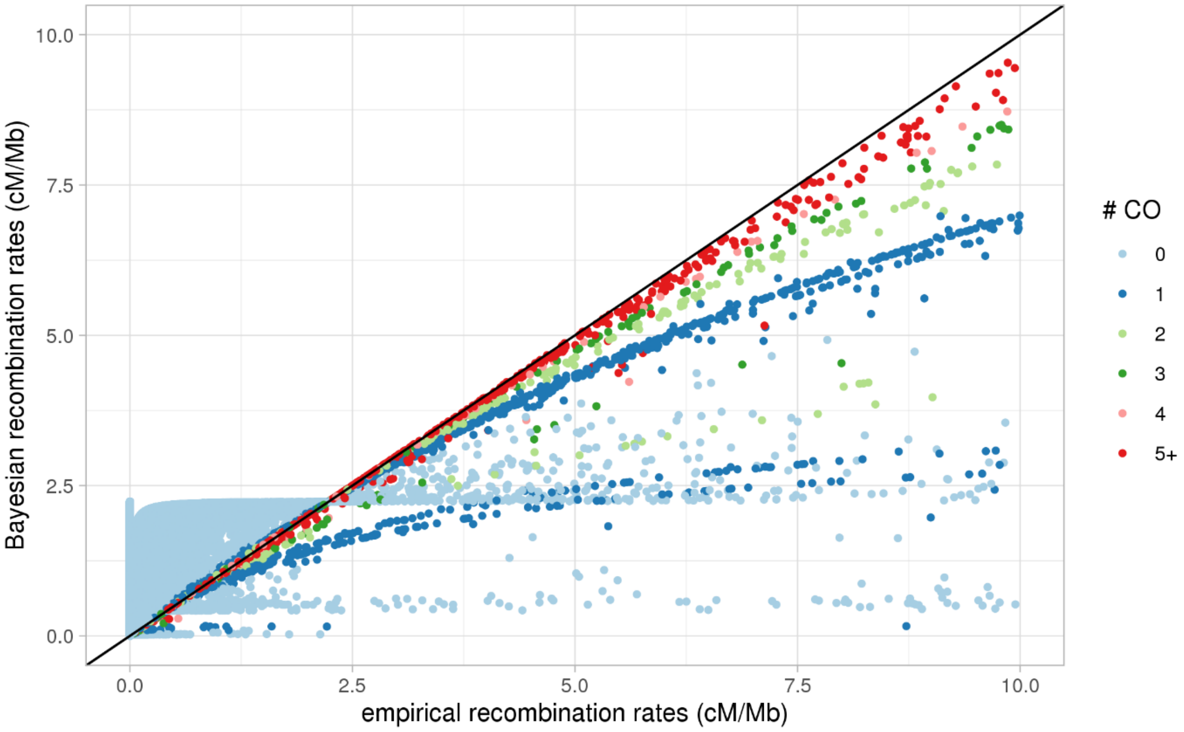

Figure S2: Comparison between Bayesian and empirical (frequentist) estimates of CsRe meiotic recombination rates. Estimates of per-meiosis recombination rate are function of RILs recombination rate estimates (resulting from more than 1 meiosis). In the Bayesian model, RILs recombination rates is $C_{i(r)}^{bay} = \frac{y_i + \alpha_r}{M\ L_i + \beta_r}$ while empirical estimates is function of $C_i^{emp} = \frac{y_i}{M\ L_i}$, where i is the interval, y_i is the number of recombination events in the interval, M is the number of RILs, L_i is the physical size of the interval and α_r and β_r the parameters of the prior Gamma (supplementary Figure S18). Both estimates are also function of uncertainty in crossover locations. The number of crossovers per interval (#CO) is the average number of crossovers assigned to each interval over 1,000 iterations. Note that Bayesian model attributes similar estimates to intervals of similar length receiving the same number of crossovers in a region. This results in vertical lines in Figure 3 of the article.

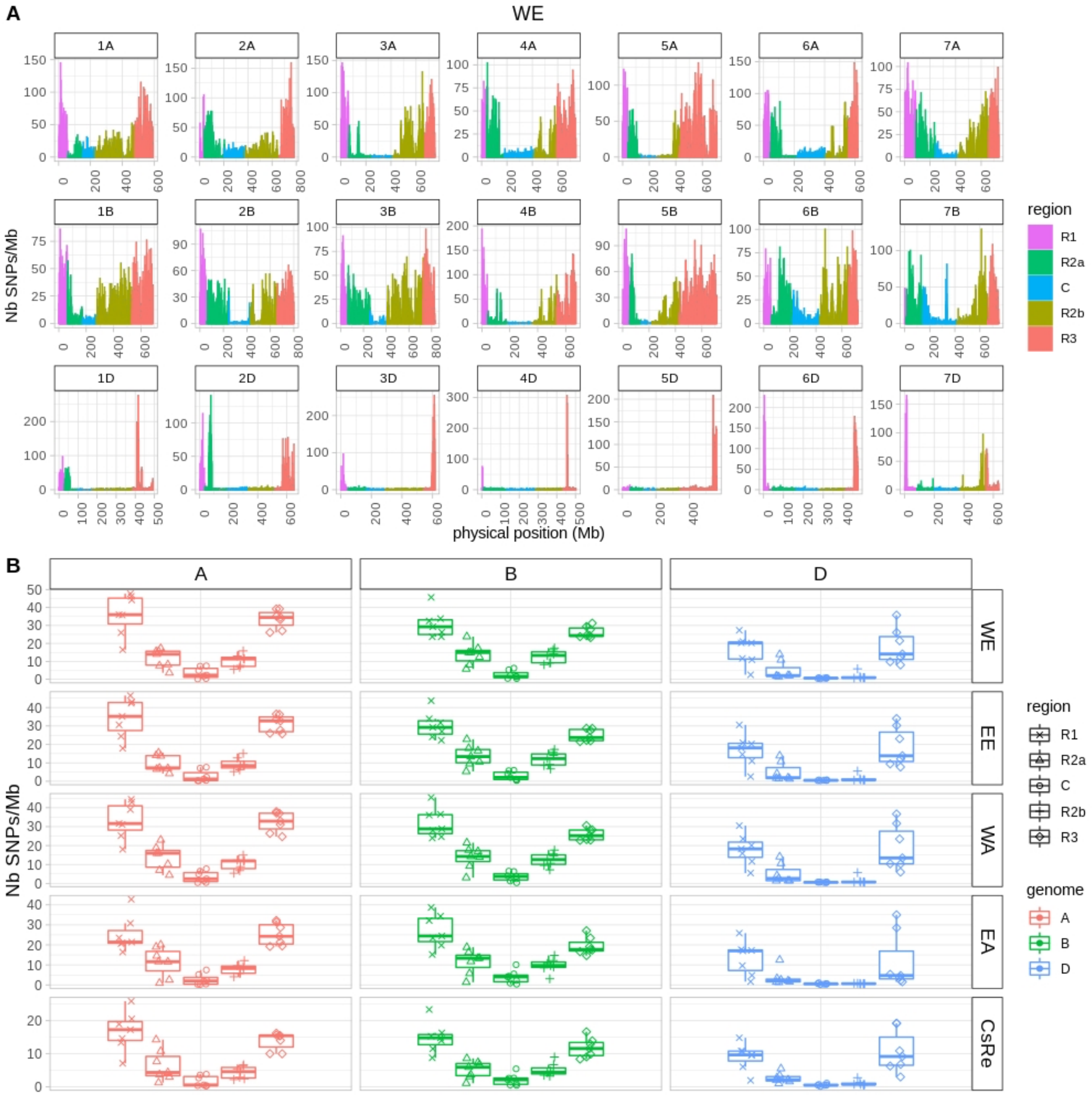

Figure S3: A. SNPs density along each chromosome for a Western European (WE) population of landraces. B. SNPs density per population, genomic region and biological origin of chromosomes. Regions R1 and R3 are telomeric regions, R2a and R2b pericentromeric regions and C are centromeric regions. Chromosomes of genome A (1A to 7A) are derived from *T. monococcum ssp. urartu* genome; chromosomes of genome B (1B to 7B) are derived from the genome of a yet-unknown species related to the *Sitopsis* section ; chromosomes of genome D (1D to 7D) are derived from *Aegilops tauschii* genome (D genome). Populations WE, EE, WA and EA are populations of unrelated landraces. The CsRe population is a biparental population of RILs.

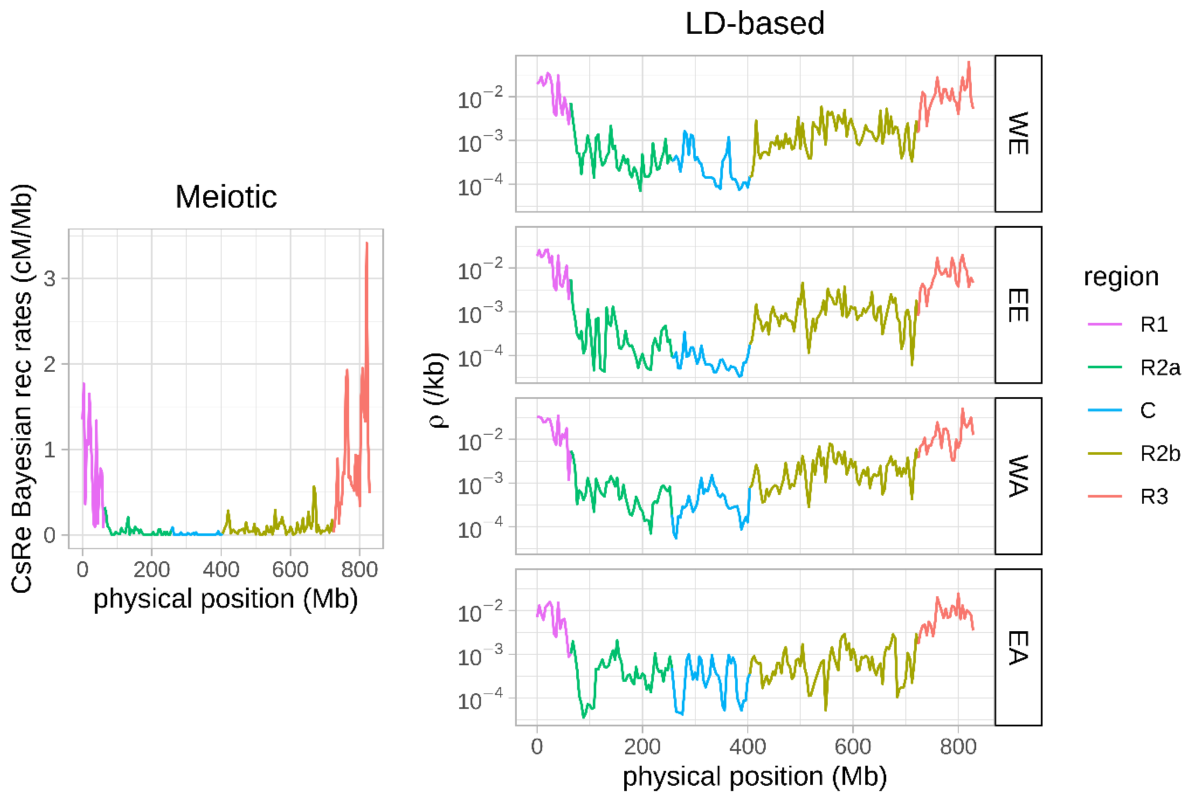

Figure S4: Meiotic and LD-based recombination profiles ($\log_{10}$ scale) in 4 Mb windows along chromosome 3B in the CsRe segregating population (left) and in the four West European (WE), East European (EE), West Asia (WA) and East Asia (EA) collections (right). Each colour corresponds to genomic regions defined by Choulet et al. (2014): highly recombining telomeres R1 (magenta) & R3 (red); low recombining pericentromeres R2a (dark green) & R2b (light green); and centromere C (blue) where recombination rates are close to 0.

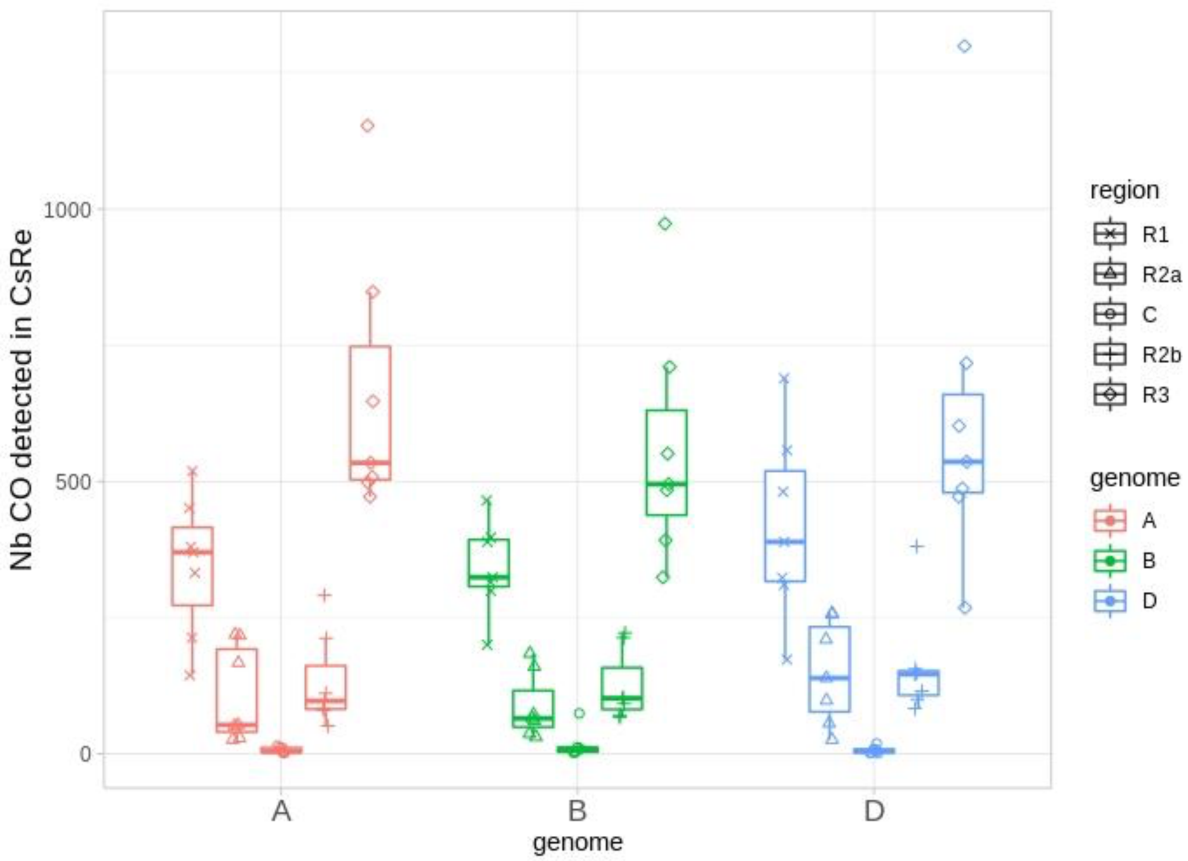

Figure S5: Number of crossovers (CO) detected per genomic region and biological origin of chromosomes in the CsRe population. Regions R1 and R3 are telomeric regions, R2a and R2b pericentromeric regions and C are centromeric regions. Chromosomes of genome A (1A to 7A) are derived from *T. monococcum ssp. urartu* genome; chromosomes of genome B (1B to 7B) are derived from the genome of a yet-unknown species related to the *Sitopsis* section ; chromosomes of genome D (1D to 7D) are derived from *Aegilops tauschii* genome (D genome). The CsRe population is a biparental population of RILs, while populations WE, EE, WA and EA are populations of unrelated landraces. One CO was counted at each parental allele switch along chromosomes in CsRe progeny.

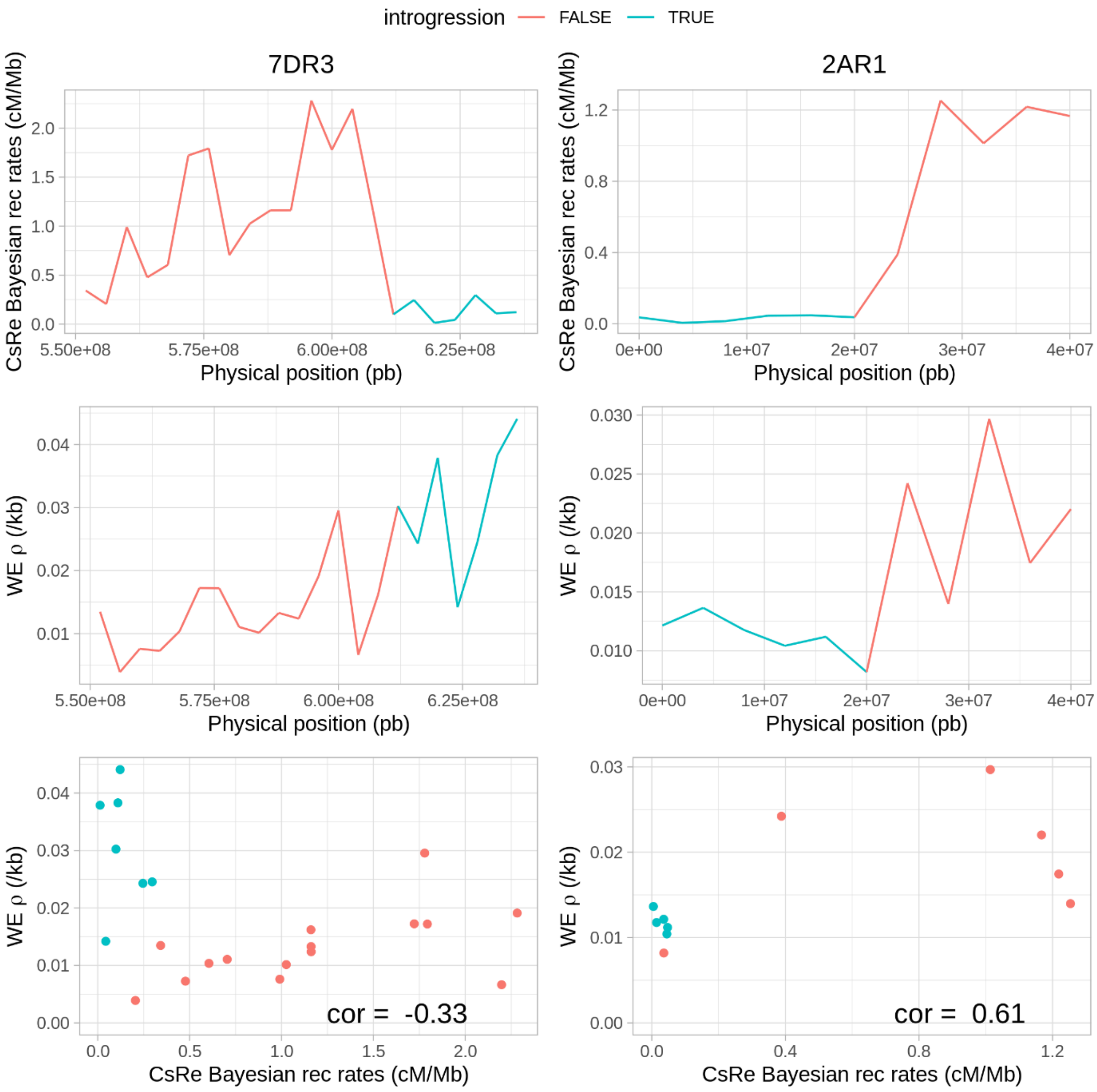

Figure S6: Recombination rates in the region R1 of chromosome 2A (left) and the region R3 of the chromosome 7D (right) where two genomic introgressions segregates in the CsRe biparental population (top panels) but not in WE landraces population (middle panels). In the 2AR1 genomic region, Renan line (one parent of the CsRe biparental population) carries an 20 Mb introgression from *Aegilops ventricosa* (*Lr37/Sr38/Yr17* resistance gene cluster). In the 7DR3 genomic region, the Renan line carries a 28 Mb introgression from *Aegilops ventricosa* (eyespot resistance gene *Pch1*). Recombination profile overlapping locations of introgressed segments in the Renan line is colored in blue while the rest of the recombination profile is colored in red. Recombination profiles are averaged within 4 Mb windows. The bottom panels give the relationship between CsRe and WE recombination profiles within each 4 Mb interval.

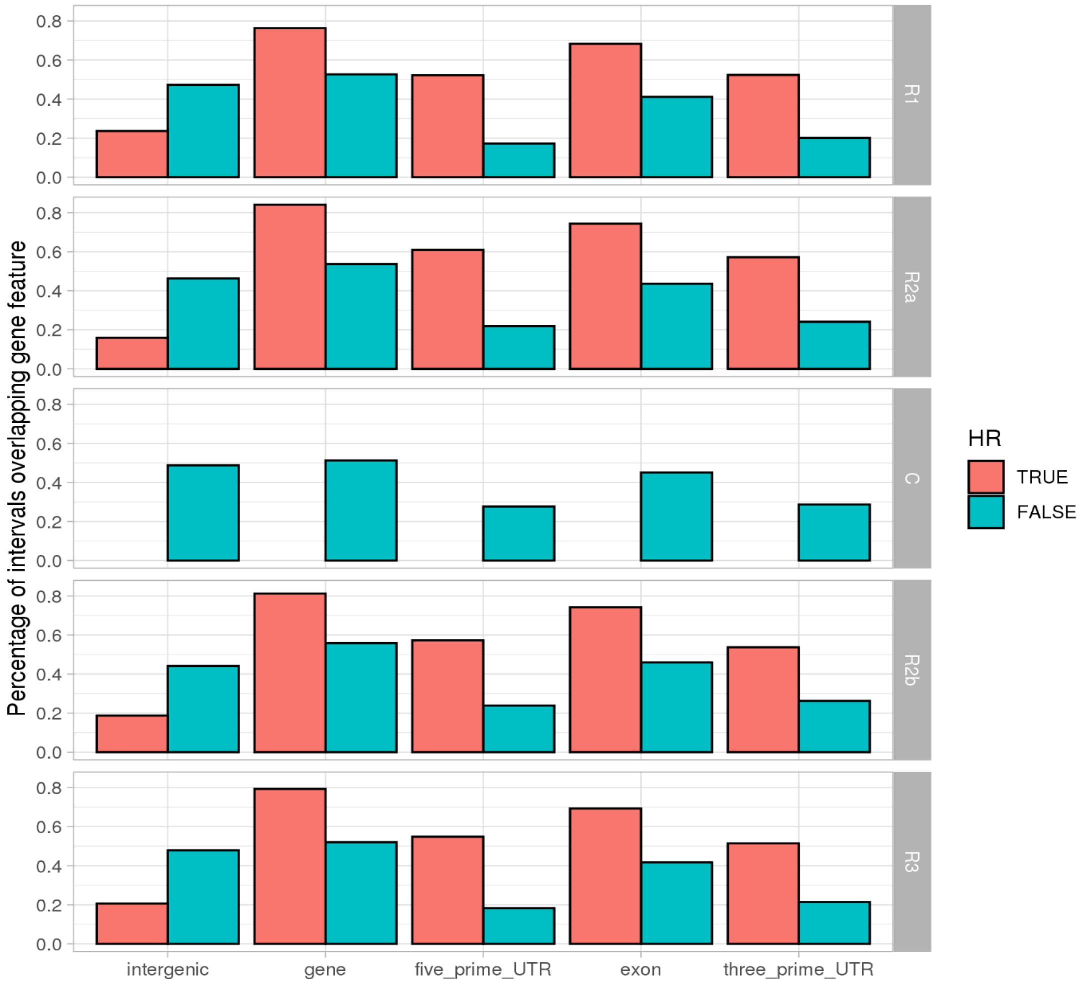

Figure S7: Proportion of Highly Recombining Intervals (HRI, red) overlapping genes features or intergenic segments compared to the proportion of non HRIs intervals (blue) overlapping genes features or intergenic segments. Position of genes features were extracted from the annotation of the RefSeq V1.0 assembly genome (IWGSC, 2018). The comparison has to be made independently within each of the 5 chromosomic regions because of decreasing density of genes and HRIs from telomeres (R1, R3) to pericentromeres (R2a, R2b) and centromeres (C) (Choulet et al. 2014). Note that the overlap was computed within each genomic region (1AR1...7DR3) and then averaged per chromosomic region (R1, R2a, c, R2b, R3). intergenic = genomic segments where no gene were annotated ; gene = genomic segment where genes were annotated, including sometimes 5'UTR, exon and 3'UTR segments within the gene segment. The proportion of HRIs co-localizing with genes and intergenic features is put in regard with the proportion of non HRIs overlapping such features, this second proportion representing here the expected overlap in a model where HRI's were randomly placed along the genome. On average, in all genomic regions, the 8,713 HRIs tend to highly co-localize with genes features compared to non HRIs intervals. For example, the proportion of HRIs overlapping genes was 80%, but dramatically decreased to 53% when considering non-HRIs intervals.

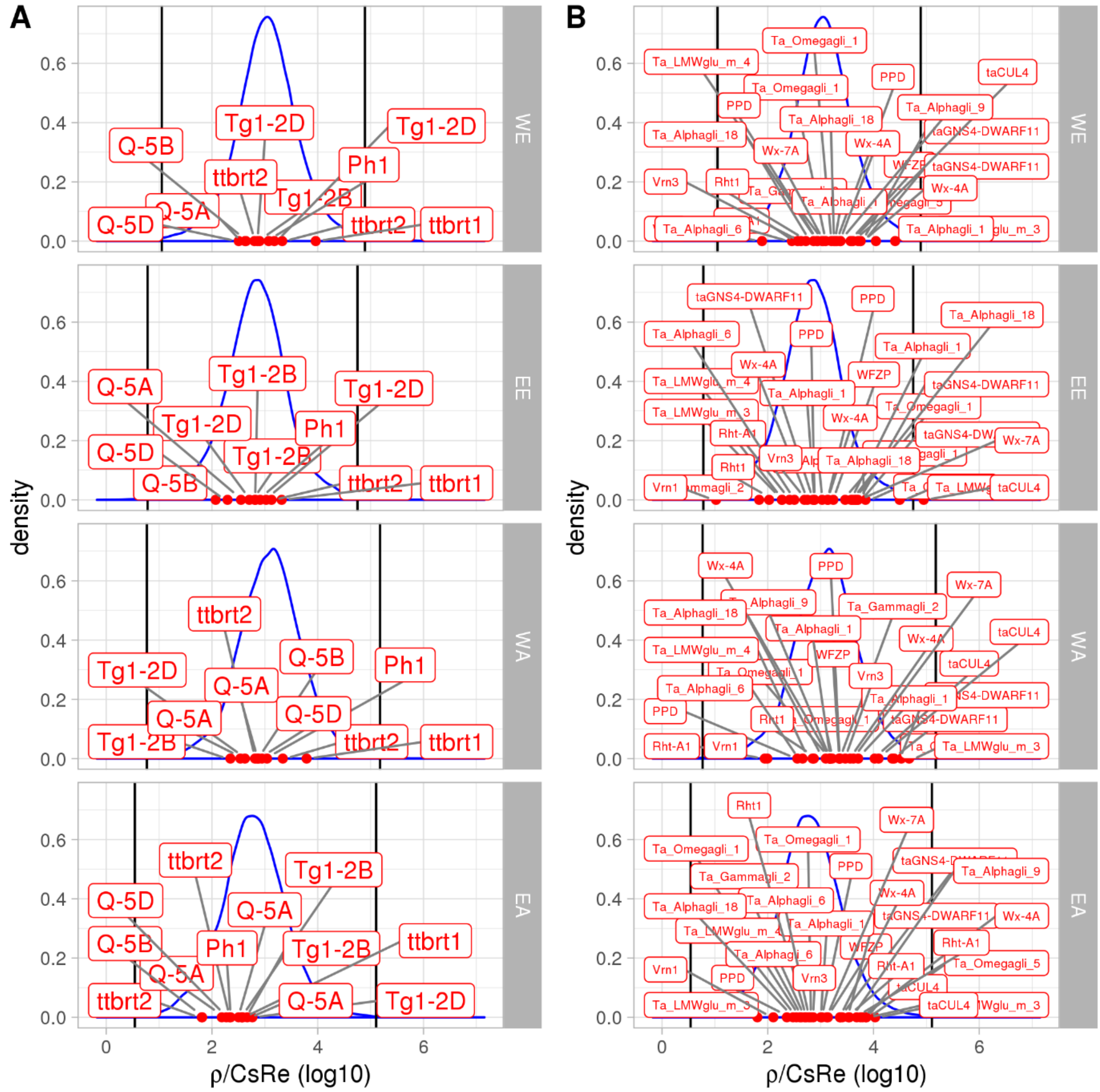

Figure S8: Comparison of LD-based and CsRe meiotic recombination rates nearby genes under selection. Blue curves represent the distribution of the ρ/CsRe ratio of each 4 Mb interval. **A** domestication genes. ttbrt = brittle rachis, Tg = tenacious glume, Ph = homoeologous pairing, Q = non-free-threshing character. **B** Improvement genes. Ppd = photoperiod sensitivity, VRN = vernalization, Rht = reduced height, Glu = Glutenin and Gli = Gliadin genes involved in seed storage protein accumulation, FZP = Frizzy panicle, GNS = grain number, Wx = waxy, as well as uniCULm = the CUL gene driving plant architecture

(names and position according Pont et al. (2019)). Vertical black lines indicate threshold of FDR ≤ 5% or FDR ≥ 5%.

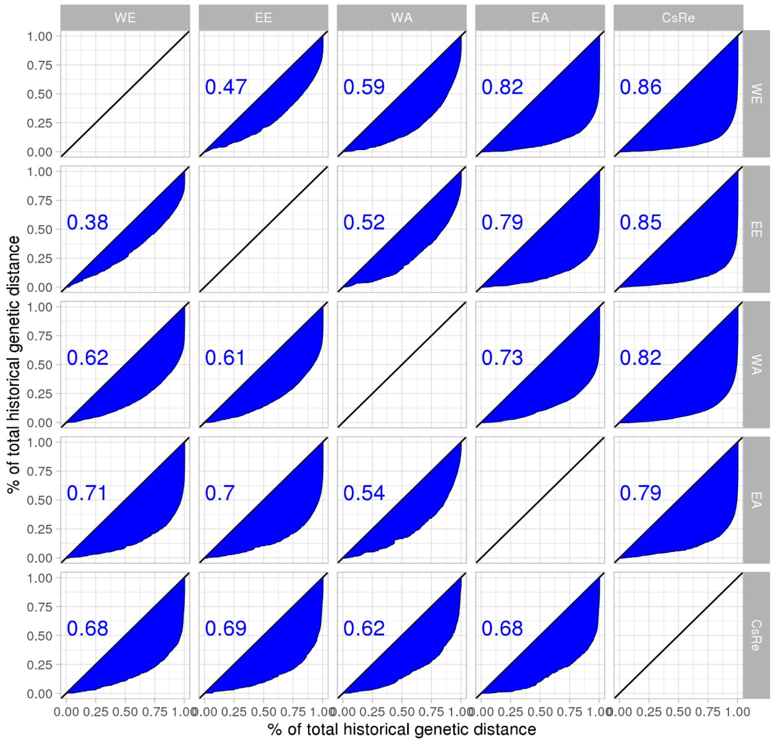

Figure S9: Comparison of distribution of genetic distance along genome in different populations. We computed the genetic distance of each interval i (defined at supplementary Figure S20) of two successive SNPs of each population this way : for WE, EE, WA and EA populations: genetic distance = LD-based recombination rates in interval i (λ_i^ *ρ_(w(i))^) * size of the interval; for CsRe population: genetic distance = c_(i(r))^bay* size of the interval). We also computed the total genetic distance of each population as the sum of genetic distances over all intervals. In each interval and each population, we computed the proportion of genetic distance by the interval as the genetic distance of the interval/total genetic distance. This allows us to compare distribution of genetic distances in intervals across populations. One possible representation of the unveness of the distribution of genetic distances across populations is given by the black curve on the graph. It gives the relationship between the proportion of genetic distance in one reference population (x-axis) and the corresponding proportion of genetic distance in another population (y-axis) considering the same intervals. On the x-axis, intervals are sorted from the interval catching the most genetic distance to the interval catching the lowest genetic distance in the reference population. In consequence, one pair of populations (example: WE-EE) yields different curves depending on which population is considered as the reference one. Another measure of unevenness of a distribution is the Gini coefficient I. Note that Gini coefficients are technically computed as the area of the blue shape, i.e. area between y = x straight line and black curve.

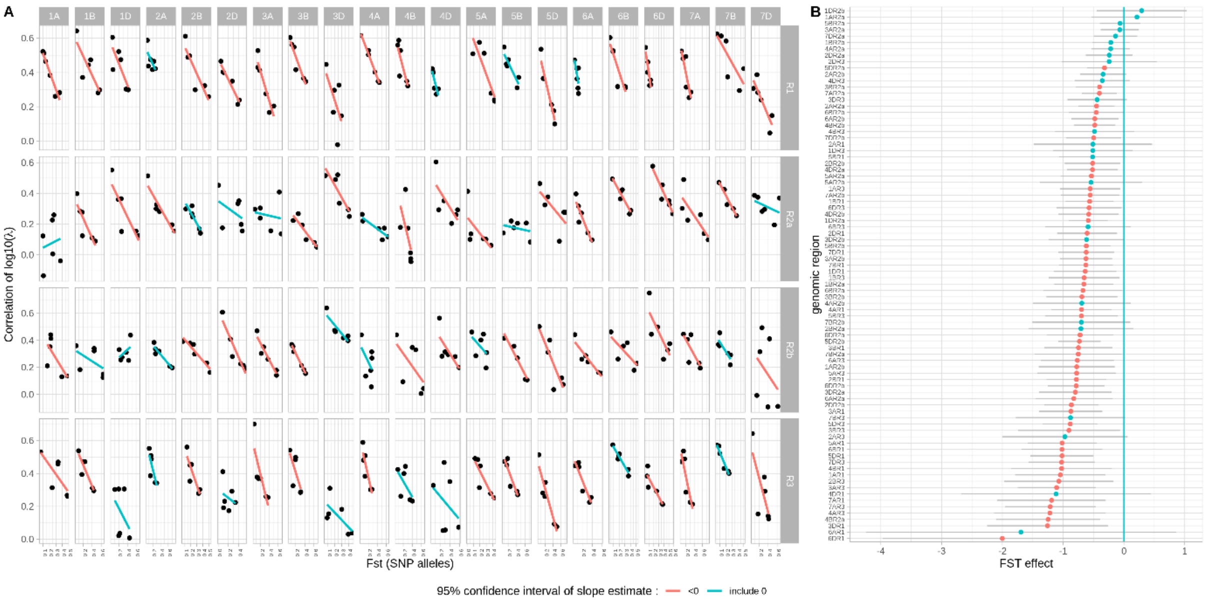

Figure S10: Relationships between correlation of local recombination intensity and F_{ST} per genomic region computed on SNPs alleles. Recombination rates are estimated from a population specific set of SNPs. A Relationship per genomic region. **B** Ranked slope estimates (coloured points) and their 95% confidence interval (grey bar). Blue colour represents slopes with a confidence interval overlapping 0 and red colour confidence interval not overlapping 0.

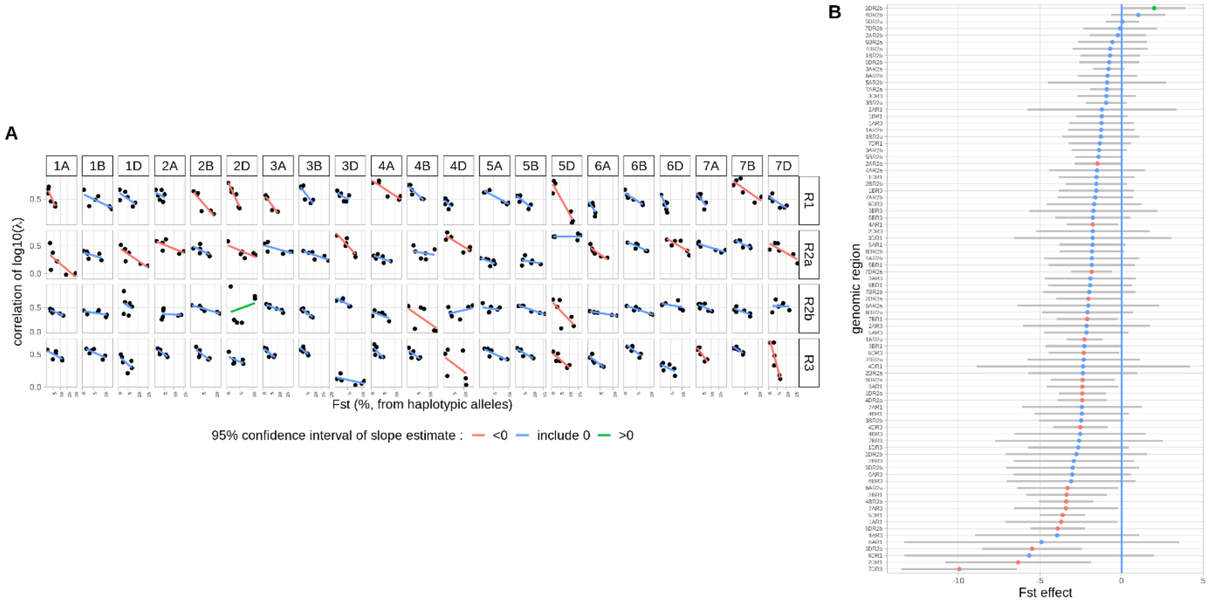

Figure S11: Relationships between correlation of local recombination intensity and F_{ST} per genomic region computed on SNPs alleles. Recombination rates are estimated using a set of SNPs which are polymorphic in all landraces populations (common SNPs dataset). A Relationship per genomic region. **B** Ranked slope estimates (coloured points) and their 95% confidence interval (grey bar). Blue colour represents slopes with a confidence interval overlapping 0 and red colour confidence interval not overlapping 0.

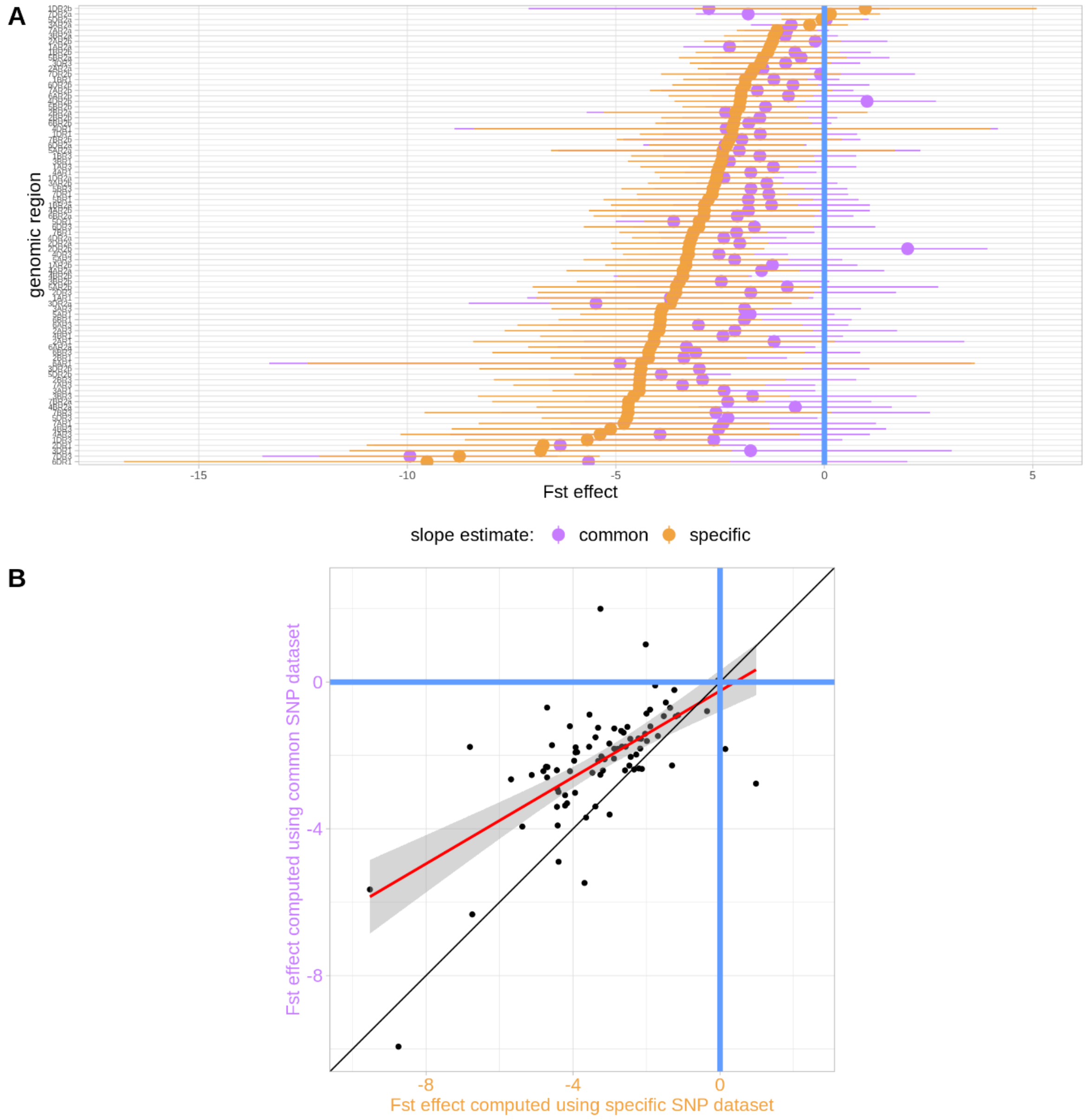

Figure S12: Comparison of slope estimates using specific (gold, Figure S10) and common (purple, Figure S11) SNPs datasets to estimate LD-based recombination rates. A. Ranking and confidence intervals of slopes estimates for each genomic region derived from either specific or common SNPs dataset. B Relationship between slopes estimates using either SNPs dataset Most slopes are negative in both dataset and there is a positive significant relationship between estimates. Slopes from the common SNPs dataset tends to be higher (*i.e.* less negativ) than slopes from specific SNPs dataset.

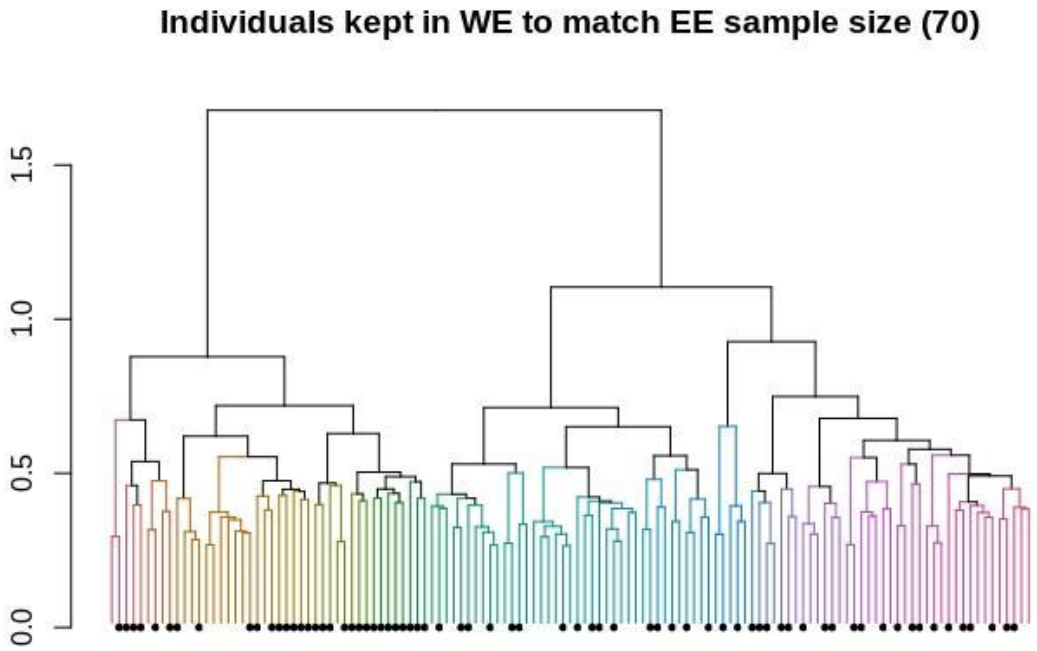

Figure S13: Hierarchical clustering of the 127 landraces of the WE population and subsampling of 70 landraces to mimic WE genetic diversity. Colored branches indicate the 70 clusters of this hierarchical clustering. Black leaves indicate the 70 sampled landraces (one per cluster) to form the WEds population.

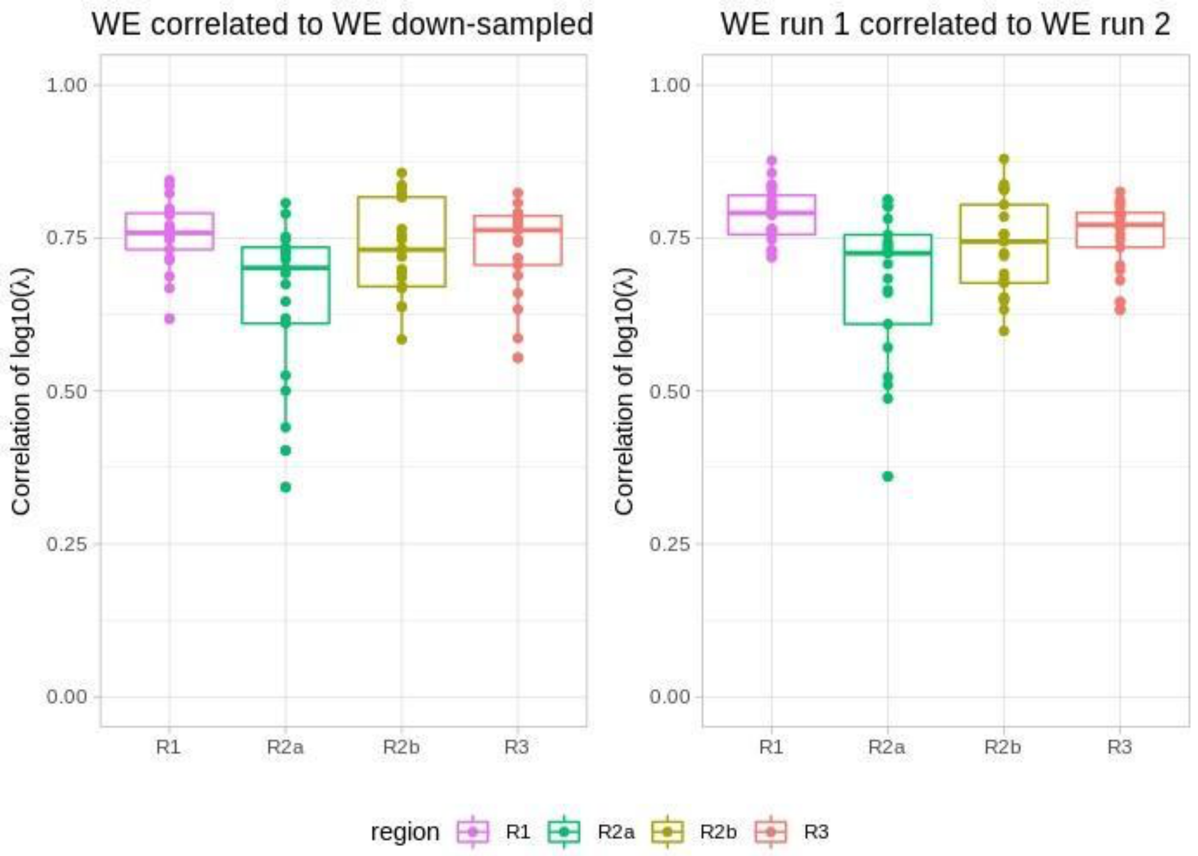

Figure S14: Average correlation of recombination profile intensity (log10(λ)) per genomic region between the WE and WEds population (left) and two independent analyses of the same population WE (right). SNPs dataset and PHASE settings are the same across the analyses presented here. **Left.** The average correlation of recombination intensity profile of the WE and WEds populations across all genomic regions was 0.72 (∓0.1) (centromeric regions not included). **Right.** The average correlation of recombination intensity profile of WE population across two independent runs of PHASE is 0.74 (∓0.1) (centromeric regions not included). As the average correlation between WE and WEds is similar to the correlation obtained between two PHASE runs, we conclude that downsampling has little effect on LD-based recombination profile.

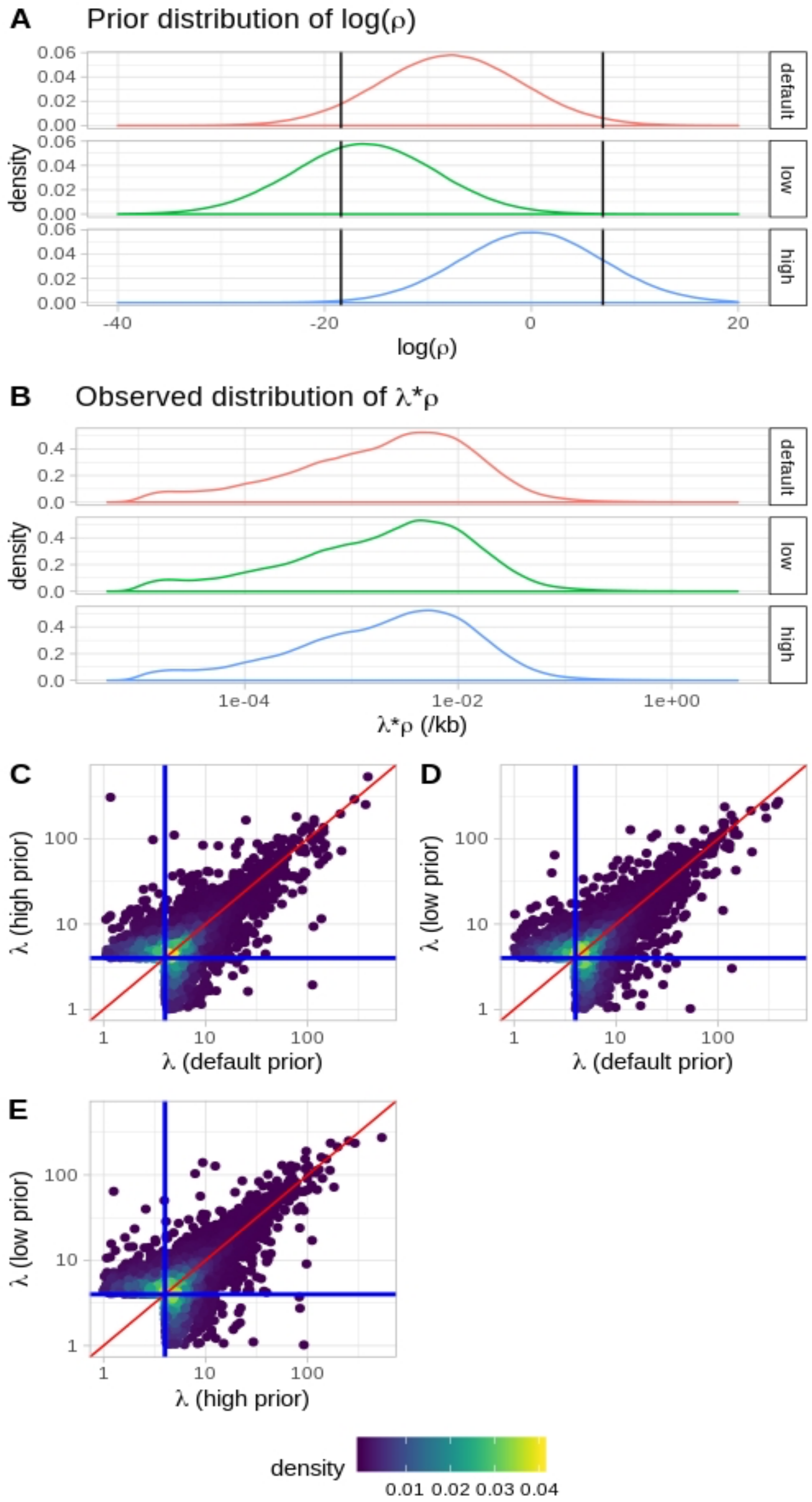

Figure S15: Robustness of PHASE inference to the prior distribution. A. The three tested prior distributions for background LD-based recombination rates: default prior distribution (mean of ρ_w = 4e-4); low prior distribution (1e-7) ; high prior distribution (1). **B**. Distributions of LD-based recombination rates estimates ($\rho_i = \lambda_i * \rho_{w(i)}$) using each prior distribution, and LD patterns of WE population. **C, D and E.** Relationships between local recombination rates intensities (λ_i) using two different prior distributions, in intervals claimed HRIs in one or both inferences in WE population.

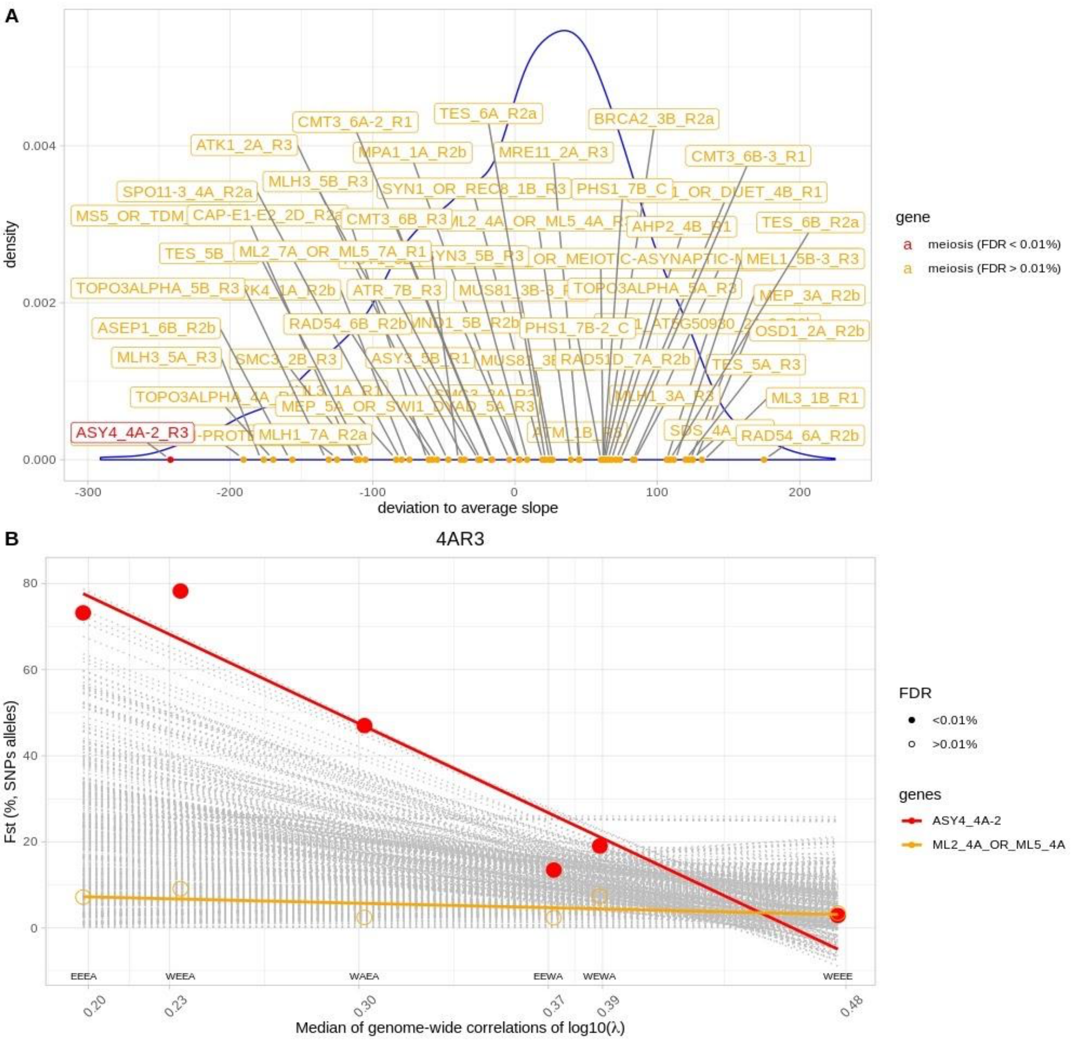

Figure S16: Differentiation of meiotic genes. Top: Distribution of deviations to average slope estimated with linear model (2) of supplementary protocol S3 for all control and meiotic genes. The average slope between F_{ST} and correlation of recombination profile is significantly negative, meaning that on average, the differentiation decreases with similarity in recombination profile. The deviation to average slope is interpreted here as the deviation to the background differentiation level. Very negative values indicate highly differentiated genes while very positive values indicate low differentiated genes. The 54 studied meiotic genes are represented by labels. Orange labels indicate meiotic genes whose deviation to background differentiation show a FDR ≥ 0.01%. Only one gene “ASY4_4A-2_R3” has a red label because its FDR is lower than 0.01%. This gene is located in the 4AR3 genomic region. **Bottom**: Representation of relationship between F_{ST} for each gene of the 4AR3 genomic region with correlation of recombination intensity profile (measured by $\log_{10}(\lambda)$). Grey slopes represent linear relationship for each of the 475 control genes sampled in the genomic region. Coloured slopes represent linear relationship for the two genes of this genomic region possibly involved in meiotic process. Empty dots indicate whether theses meiotic genes show a FDR lower than 0.01%. As we can see, the slope “ASY4_4A-2_R3” gene is very different from all others genes and very differentiated. At the opposite, the “ML2_4A” gene shows a low differentiation.

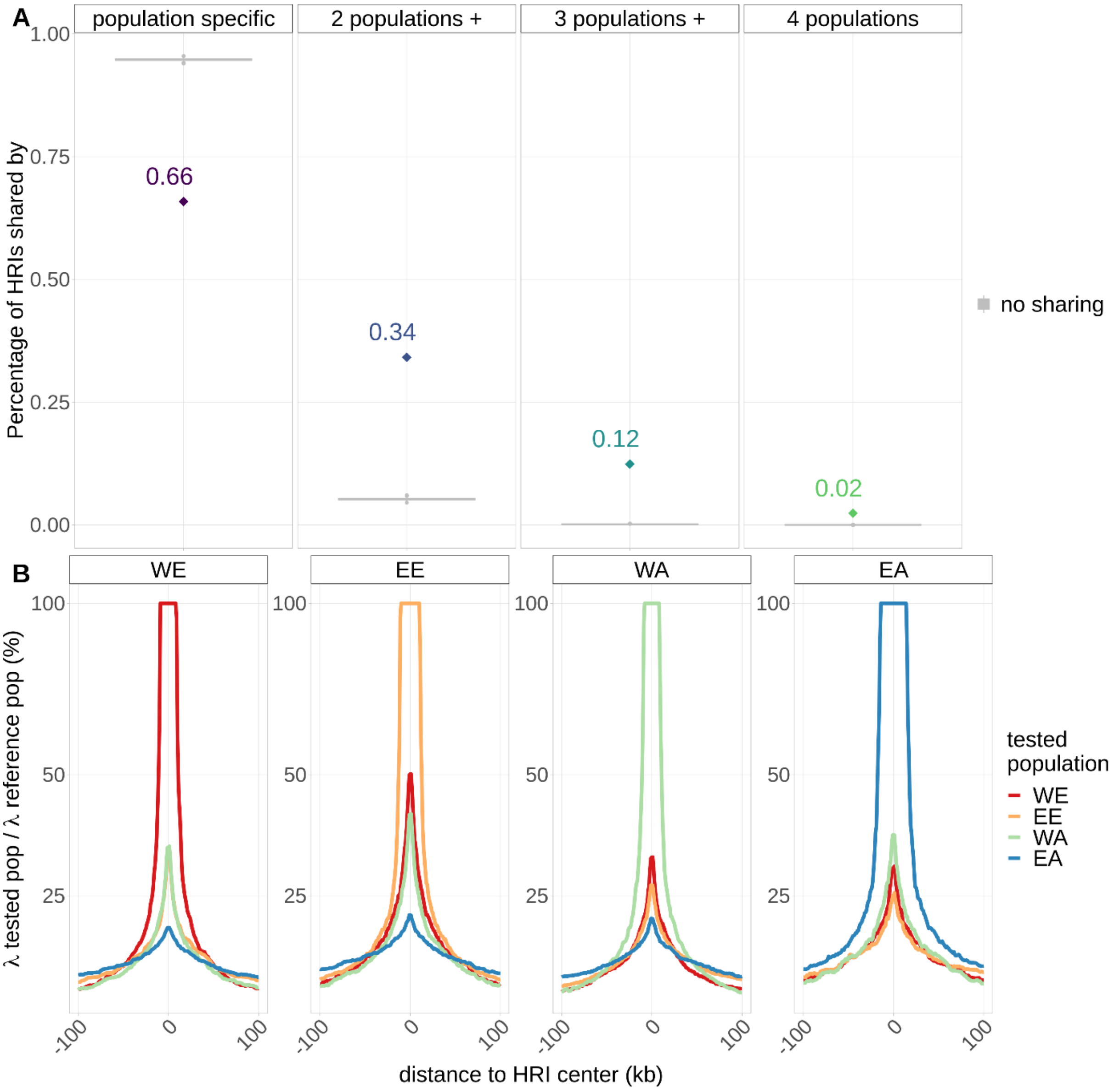

Figure S17: Proportion of shared HRIs when estimating LD-based recombination profiles on a common SNPs dataset for all populations. LD-based recombination rates were estimated using the same SNPs dataset for all populations (*i.e.* SNPs which are polymorphic in all populations). **A.** Proportion of co-localizing HR (coloured points) and simulated co-localizing values under random assignment of HRIs (grey boxplots) **B.** LD-based recombination intensity in each of the four populations WE, EE, WA and EA around HRIs specific to one population.

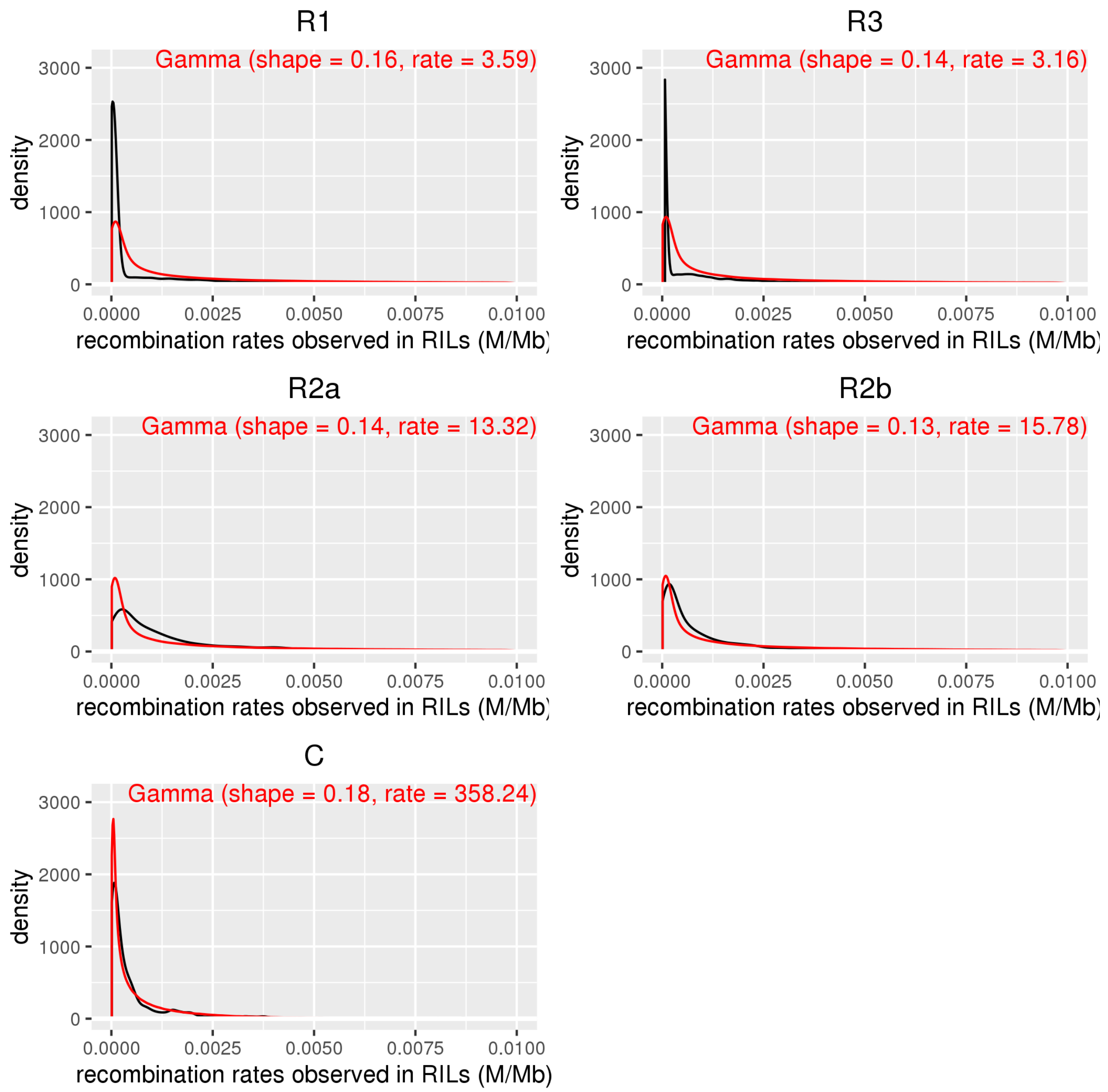

Figure S18: Prior distributions for RILs Bayesian recombination rates. The black curve represents the distribution of frequentist recombination rates in RILs and the red curve the fitted Gamma distribution. The number of data points per black curve ranged from 4,799 in centromeric regions C to 28,756 in R3 regions, so that a single interval had a very low contribution when adjusting the Gamma distribution. The difference between the black and red distributions illustrates how prior inflates or shrinks RILs recombination rates in Bayesian model. While 60% of intervals had null frequentist estimates of recombination rates, they were replaced by the minimum recombination rates observed in the region to allow fitting a Gamma distribution (1.8e-3 M/Mb for R1, 2.5e-4 for R2a, 1e-6 for C, 1.7e-4 for R2b and 7.4e-4 for R3). However, those null intervals created a mass in empirical recombination rate distribution, decreasing the Gamma fitting quality. Note that the ratio between shape and rate for each region gives the average value of recombination rate in the region. Bayesian estimates would converge toward those average values in case of very low informativity of intervals (no recombinant, small size of intervals).

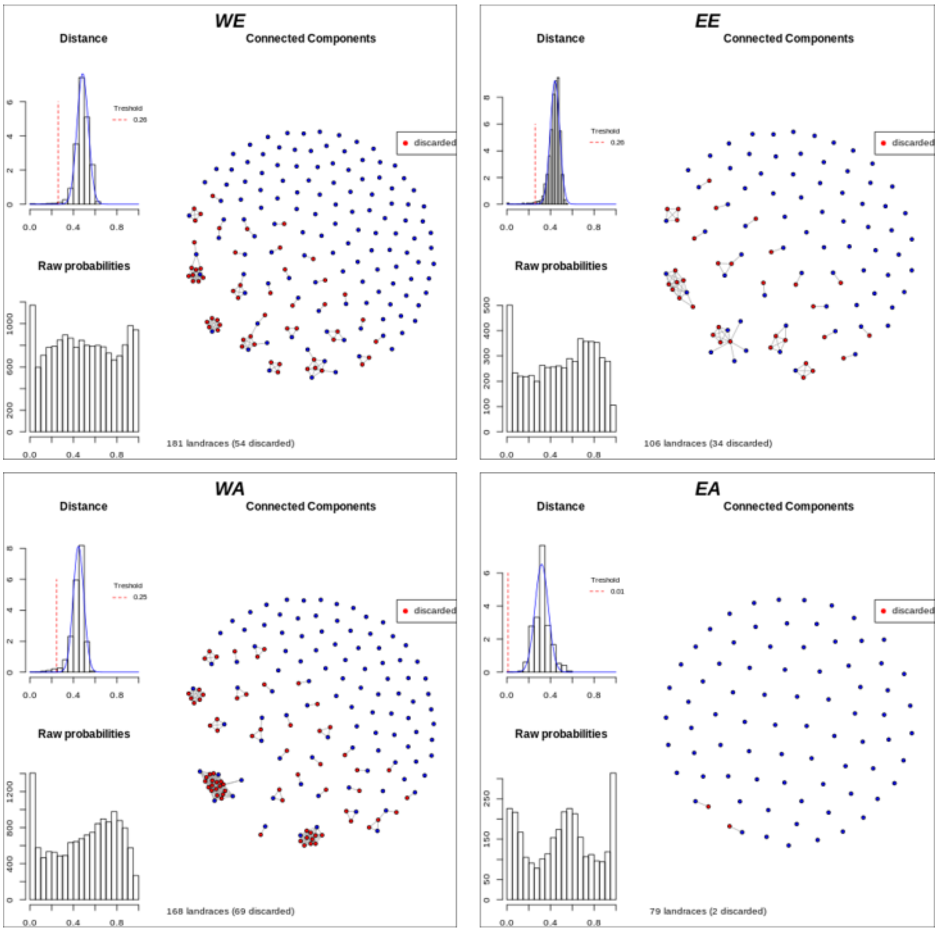

Figure S19: Identification and suppression of closely related landraces. Top left Distribution of simple matching distances, computed between each pair of landraces. Blue curve: Normal distribution to model similarity in the population, whose parameters were estimated using a Robust Fitting of Linear Models function in R, which is robust to outliers. Red dashed line: significant threshold to identify closely related landraces. **Bottom left** Distribution of P-values after, expected to follow a Uniform distribution if the simple matching distances distribution would match exactly the modelled Normal distribution. This P-values distribution was used as input in qvalue function to compute False Discovery Rate (FDR). A FDR of 1e-3 was used to set the significant threshold. **Right** Representation of landraces relationship. Each point represents one landrace. Each strait line represents a close relationship. Red points correspond to landraces that were discarded to eliminate close relationships.

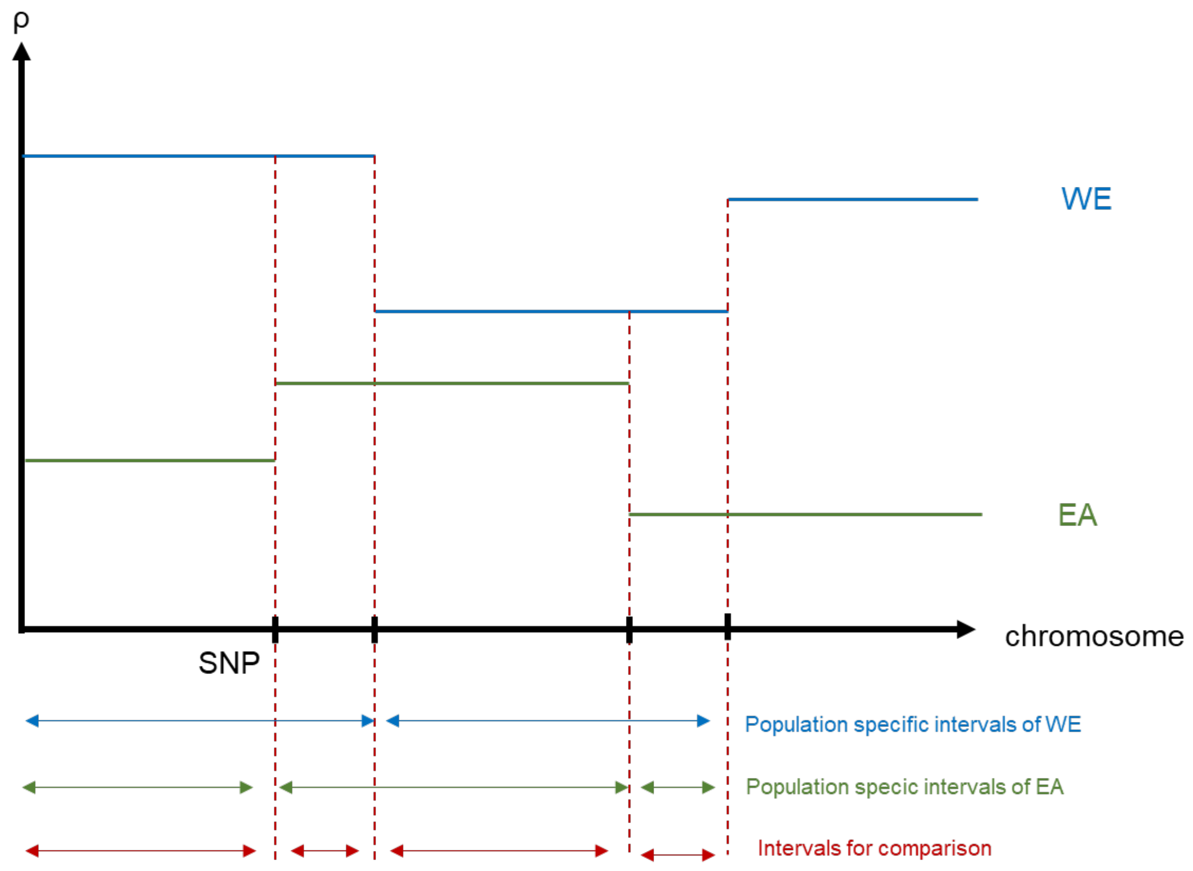

Figure S20: Defining intervals to compare fine-scale LD-based recombination rates using mixed models. Example with two populations WE and EA. LD-based recombination rates are not estimated in the same intervals in WE, EE, WA and EA because of the MAF filtering of SNPs. To compare LD-based recombination rates of the four populations, we defined intervals constituted of polymorphic SNPs in at least one of the four populations.

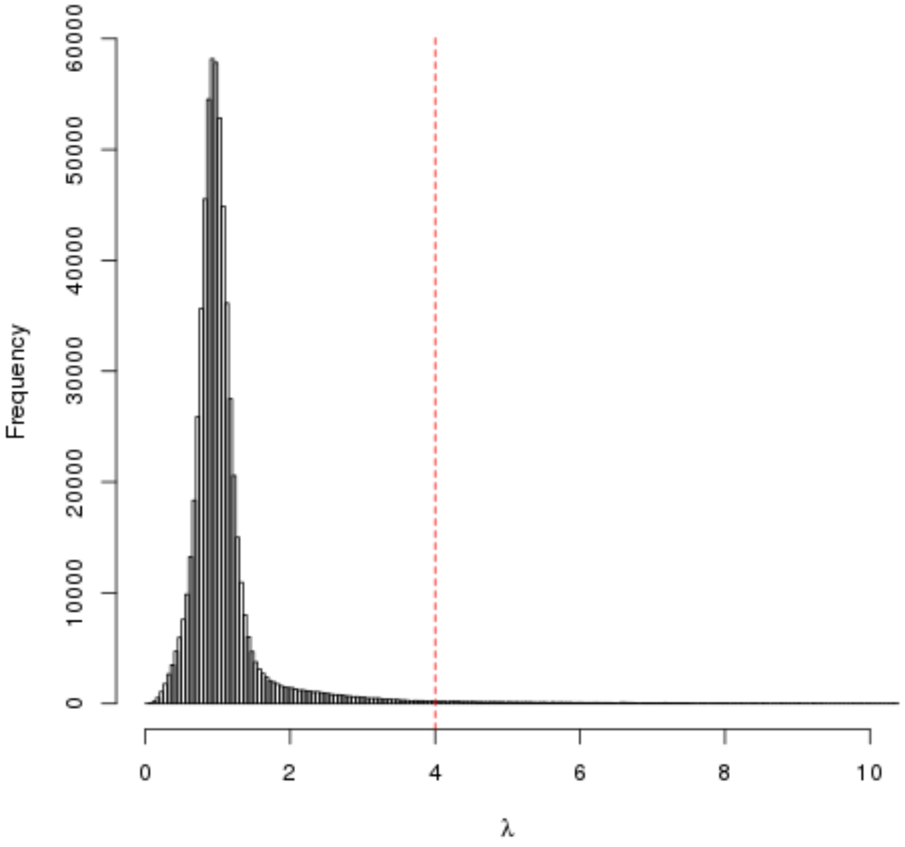

Figure S21: Distribution of LD-based recombination intensities λ for all intervals in all populations. Intervals with $\lambda \geq 4$ are claimed HRIs.

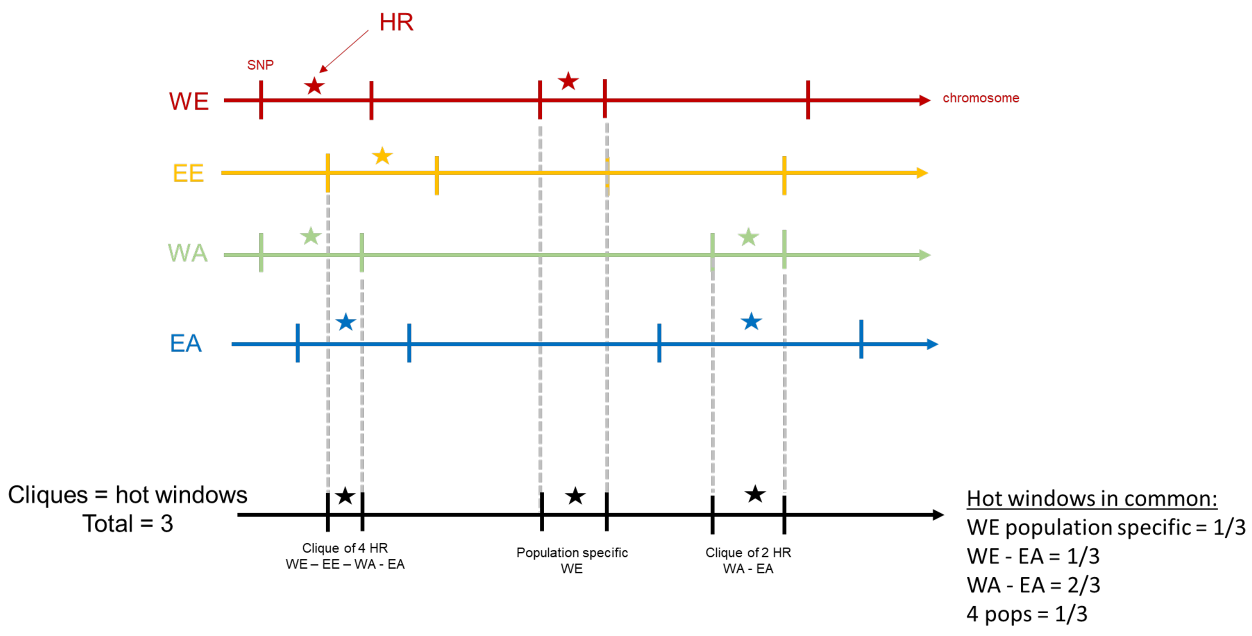

Figure S22: Examples of shared hot windows. A clique is defined as the systematic mutual overlap of HRIs of different populations. When a HRI doesn't overlap any HRI in other populations, it is called population specific. Upper and lower limits of hot windows are the inner join of HRIs borders.

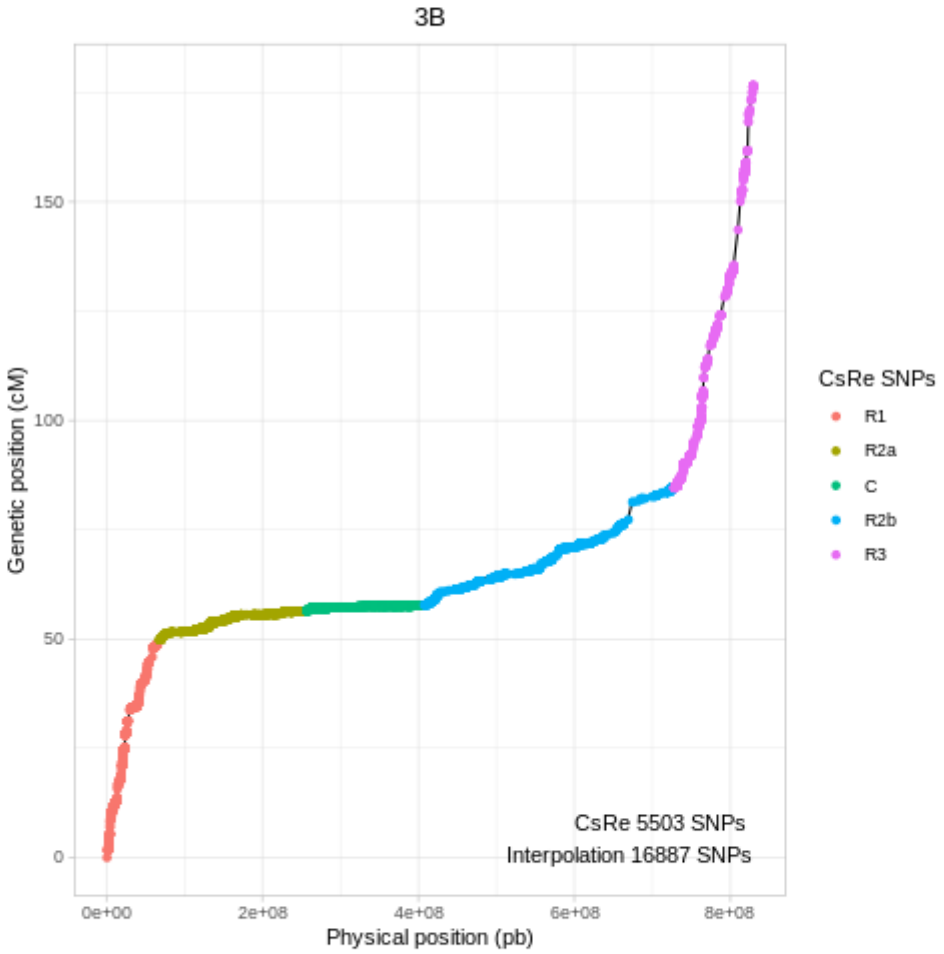

Figure S23: Interpolation of genetic position of SNPs located on 3B chromosome using CsRe Bayesian map. From mapped markers in CsRe population, we estimated the genetic position of umapped markers based on their physical position, by hypothesizing constant recombination rates within intervals of two successive mapped markers (supplementary protocol S5).

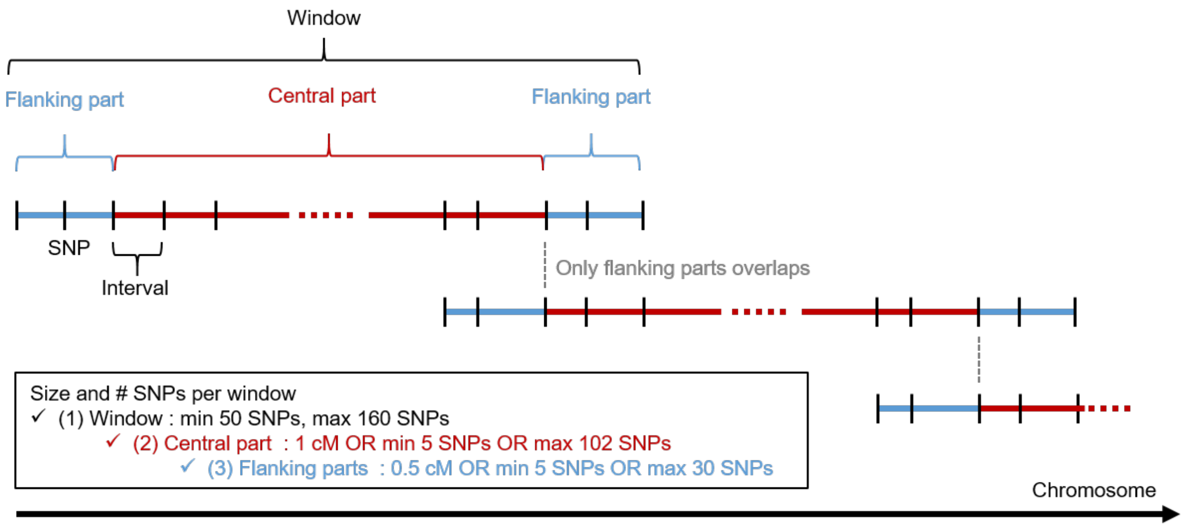

Figure S24: Definition of PHASE windows. To control both the genetic size of the window and the number of SNPs per window, we had to define constraints to form adequate windows, described in the black square (more details in supplementary protocol S5).

.

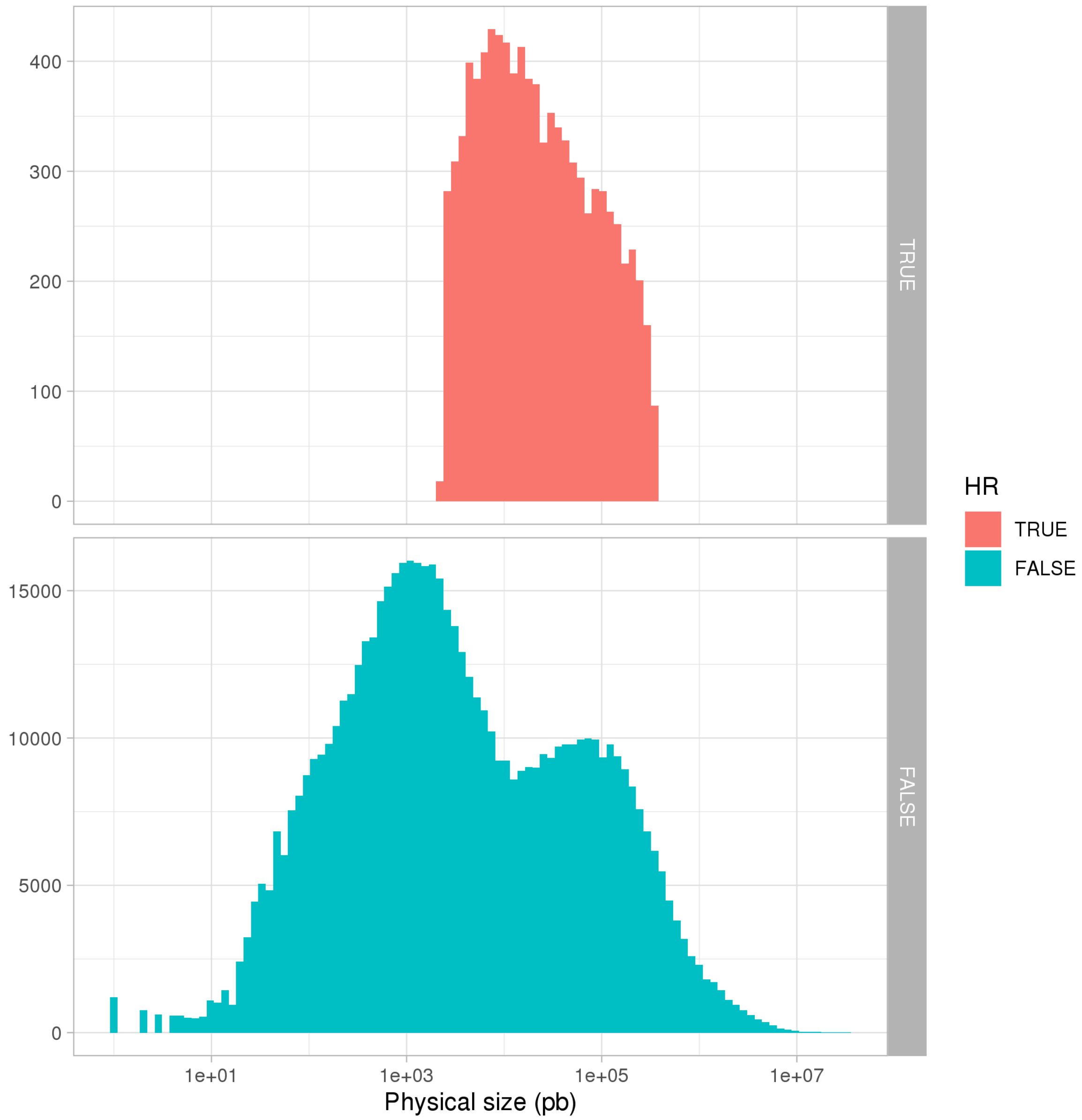

Figure S25: Distribution of physical size of Highly Recombing Intervals (HRIs, red) and other intervals (blue). The filtering procedure yielded 8,713 HRIs, with size ranging from 2,369 to 344,607 pb.

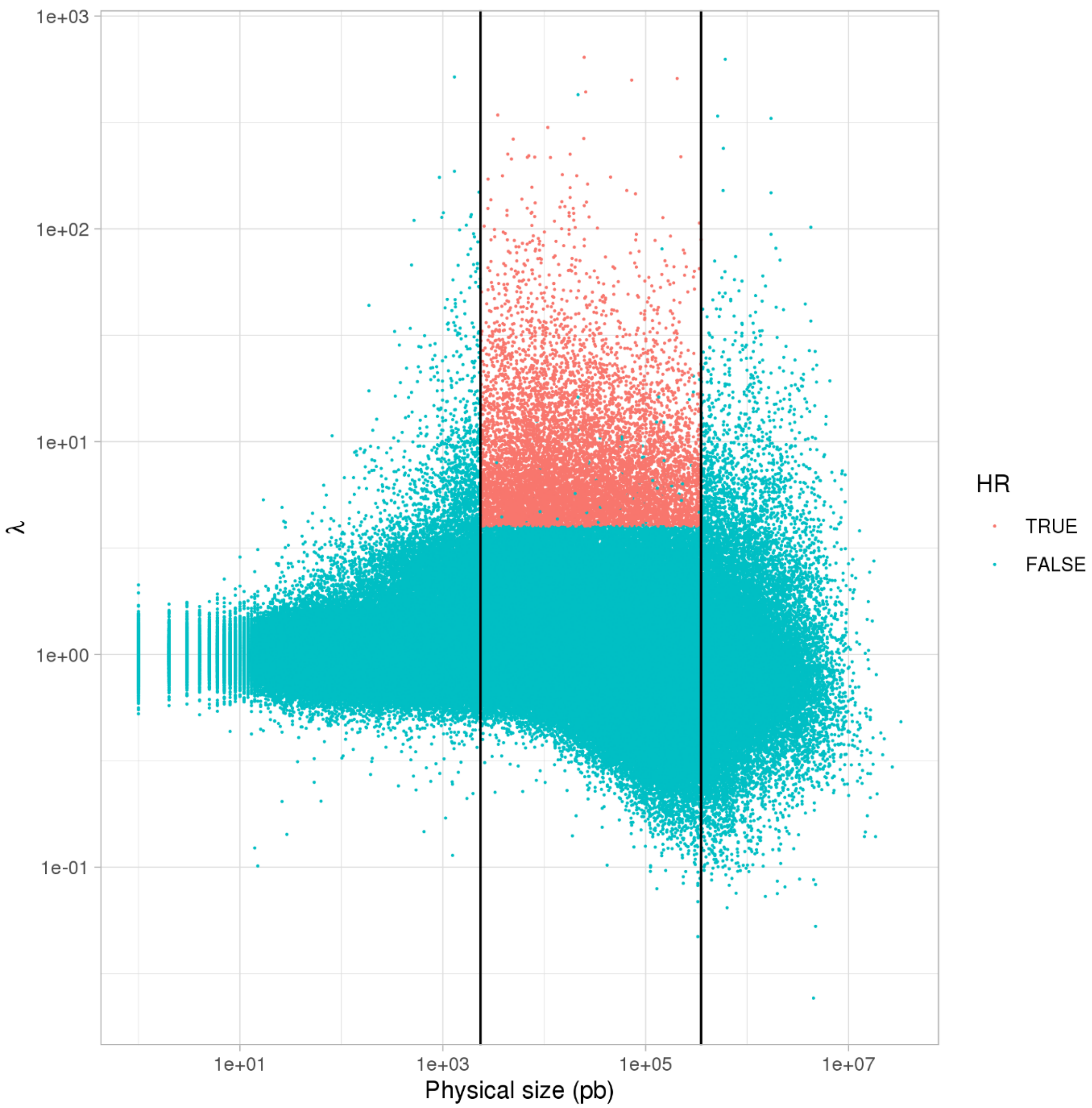

Figure S26: Relationship between historical recombination intensities λ related to the physical size of intervals. Note that intensity of Highly Recombining Intervals (HRIs) was quite independent of their physical size.

References

Balfourier F, Bouchet S, Robert S, De Oliveira R, Rimbert H, Kitt J, Choulet F, Paux E. 2019. Worldwide phylogeography and history of wheat genetic diversity. Sci Adv. 5(5):eaav0536.

Gini C. 1936. On the measure of concentration with special reference to income and statistics. Colo Coll Publ Gen Ser. 208:73–79.

IWGSC. 2018. Shifting the limits in wheat research and breeding using a fully annotated reference genome. Science. 361(6403):eaar7191.

Pont C, Leroy T, Seidel M, Tondelli A, Duchemin W, Armisen D, Lang D, Bustos-Korts D, Goué N, Balfourier F, et al. 2019. Tracing the ancestry of modern bread wheats. Nat Genet. 51(5):905–911.

Stephens M, Smith NJ and Donnelly P. 2004 Documentation for PHASE, version 2.1. Chapter 6.2, p16. url: http://stephenslab.uchicago.edu/assets/software/phase/instruct2.1.pdf.

Stephens M, Carbonetto P, Gerard D, Lu M, Sun L, Willwerscheid J and Xiao N. 2020. ashr: Methods for Adaptive Shrinkage, using Empirical Bayes. R package version 2.2-47.

Storey JD, Andrew JB, Dabney A, David R. 2015. qvalue: Q-value estimation for false discovery rate control. R Package Version 2100.

Venables WN, Ripley BD. 2002. Modern applied statistics (Fourth S., editor) New York. Springer.
