# Supplementary material for: Evolution of Recombination Landscapes in Diverging Populations of Bread Wheat
Source: Genome Biol Evol. 2021 Jun 29;13(8):evab152. doi: 10.1093/gbe/evab152 (PMC8350361; doi:10.1093/gbe/evab152)
Supplement: evab152_Supplementary_Data [file evab152_supplementary_data.zip › supplementary_file_S2_recombination_profiles_all_chr.pdf]

1A

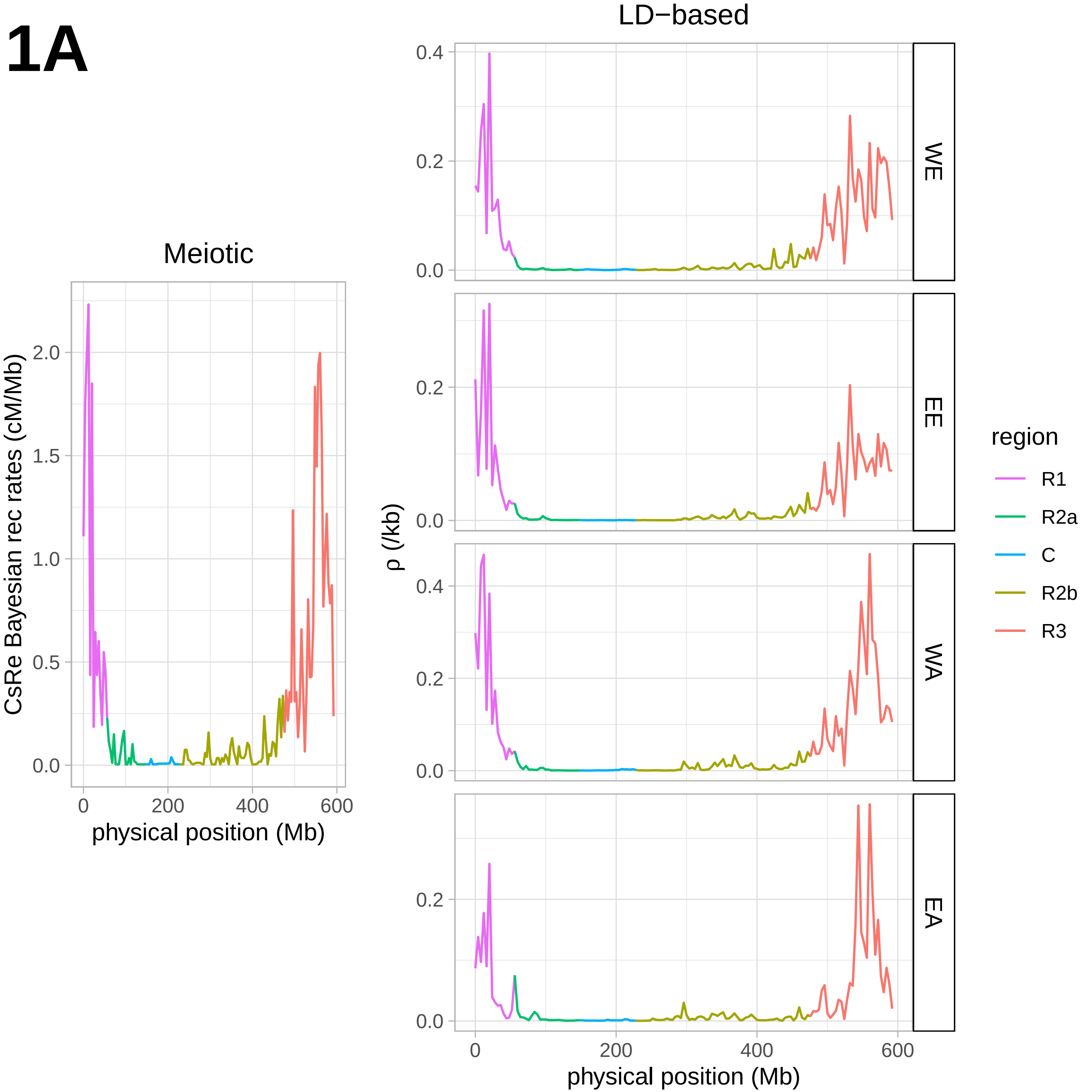
Meiotic
CsRe Bayesian rec rates (cM/Mb)
0.0
0.5
1.0
1.5
2.0
0
200
400
600
physical position (Mb)
LD−based
ρ (/kb)
0.0
0.2
0.4
WE
EE
WA
EA
region
R1
R2a
C
R2b
R3

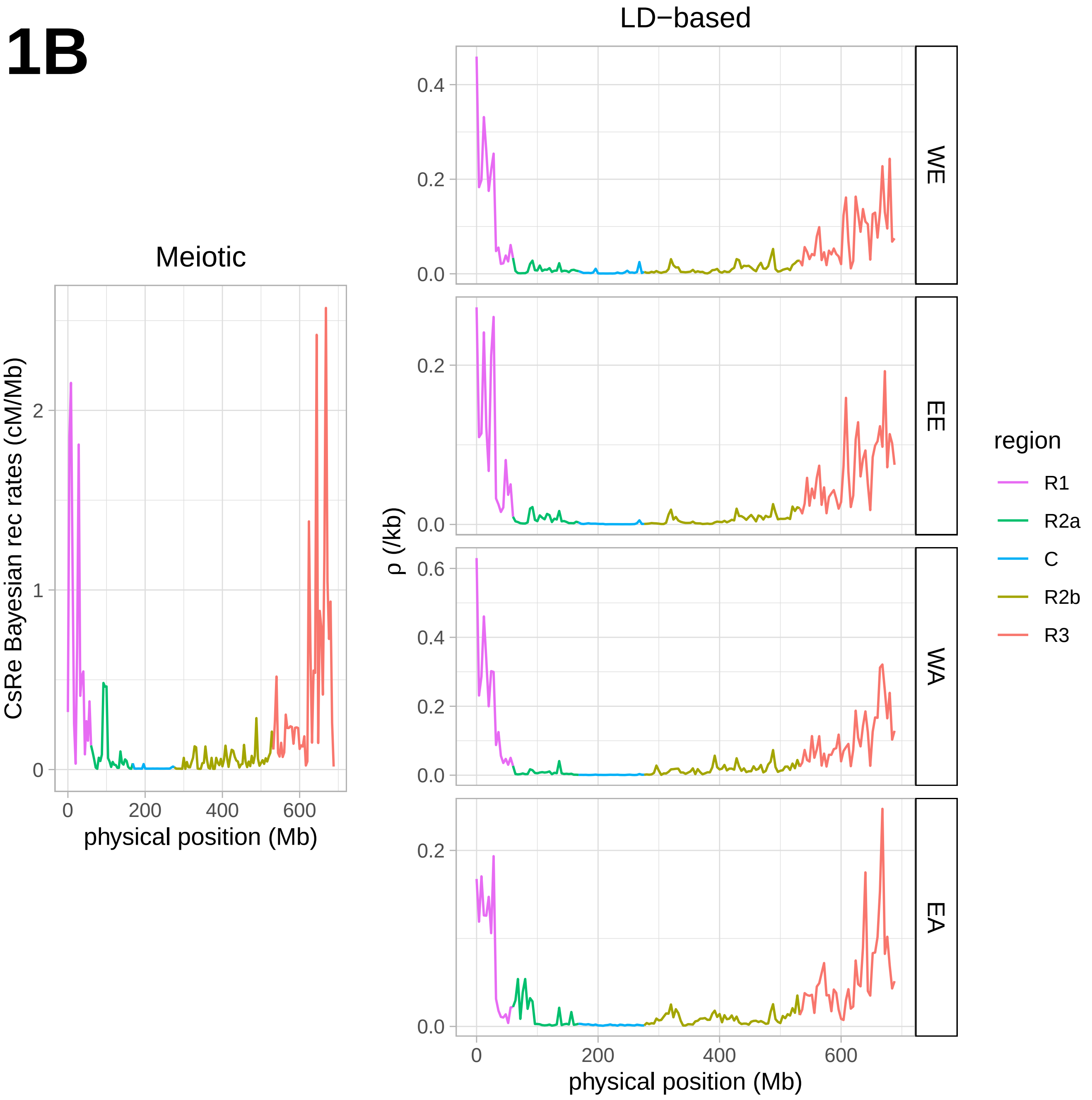
1B
Meiotic
LD−based
CsRe Bayesian rec rates (cM/Mb)
ρ (/kb)
physical position (Mb)
physical position (Mb)
WE
EE
WA
EA
region
R1
R2a
C
R2b
R3
0
1
2
0.0
0.2
0.4
0.6
0
200
400
600

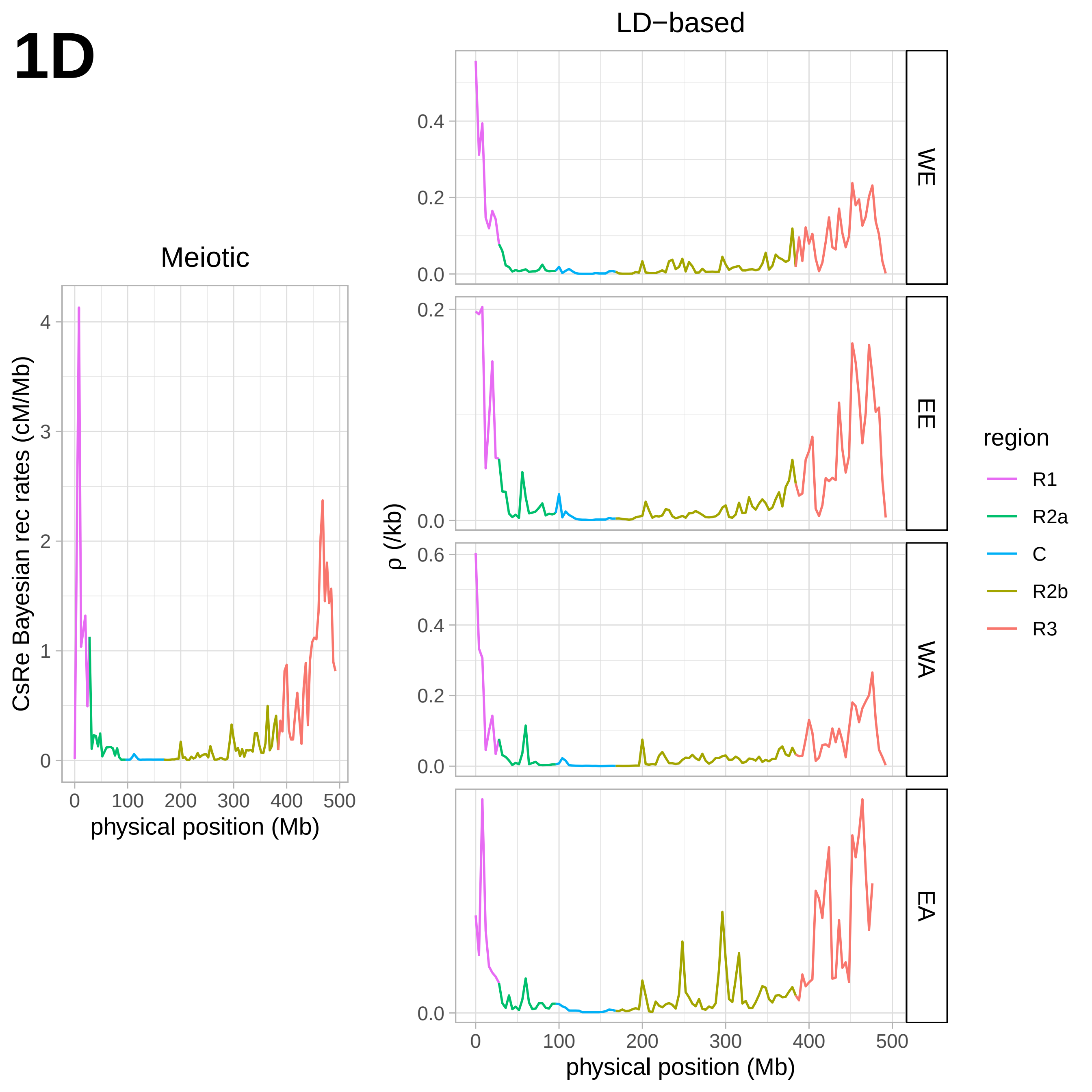

1D
Meiotic
LD−based
CsRe Bayesian rec rates (cM/Mb)
ρ (/kb)
physical position (Mb)
physical position (Mb)
WE
EE
WA
EA
region
R1
R2a
C
R2b
R3
0
1
2
3
4
0.0
0.2
0.4
0.6
0
100
200
300
400
500

2A

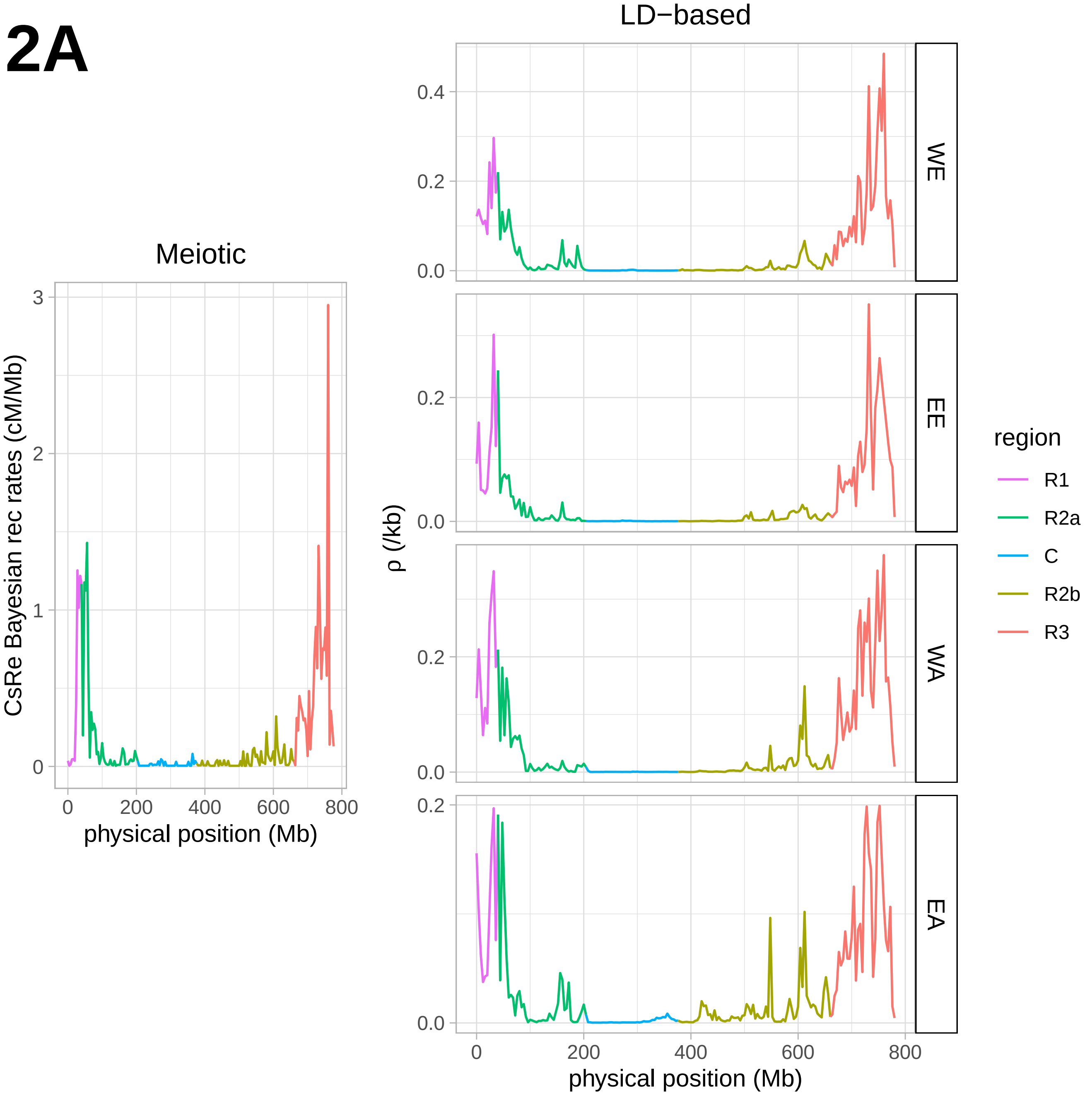

Meiotic
CsRe Bayesian rec rates (cM/Mb)
physical position (Mb)
LD−based
ρ (/kb)
WE
EE
WA
EA
physical position (Mb)
region
R1
R2a
C
R2b
R3

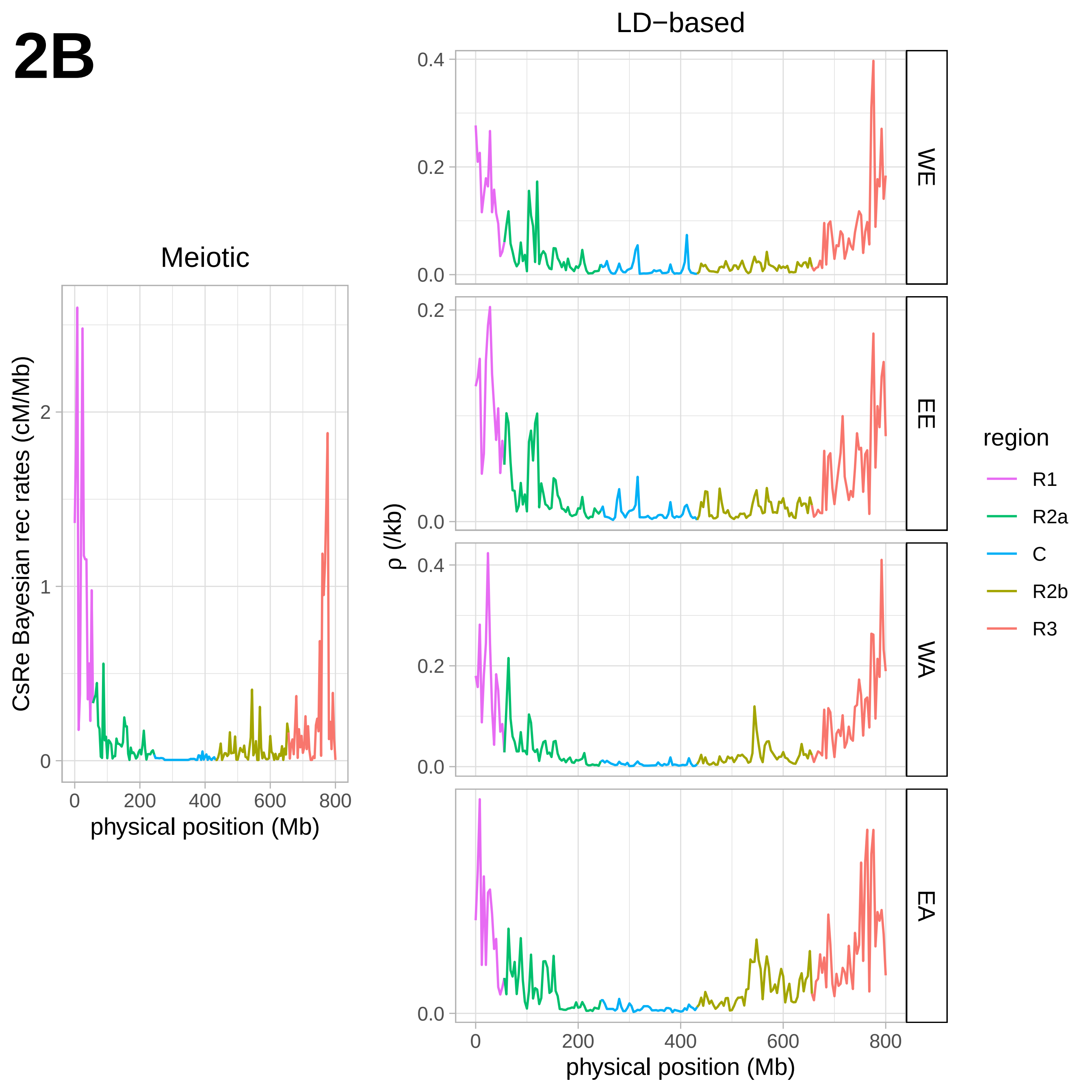
2B
Meiotic
CsRe Bayesian rec rates (cM/Mb)
0
1
2
0
200
400
600
800
physical position (Mb)
LD−based
ρ (/kb)
0.0
0.2
0.4
WE
EE
WA
EA
region
R1
R2a
C
R2b
R3

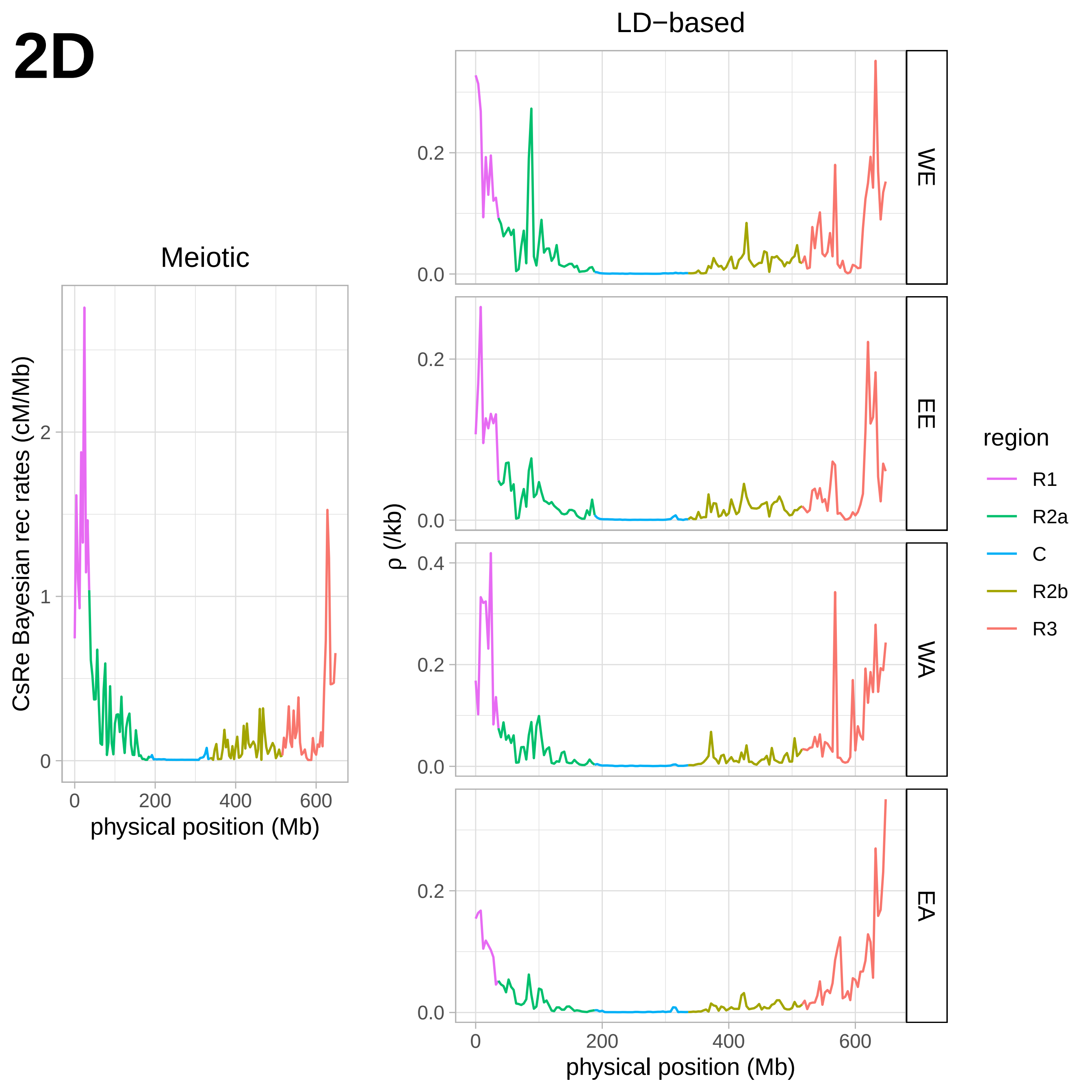
2D
Meiotic
CsRe Bayesian rec rates (cM/Mb)
physical position (Mb)
LD−based
ρ (/kb)
WE
EE
WA
EA
region
R1
R2a
C
R2b
R3
0
1
2
0.0
0.2
0.4
200
400
600

3A

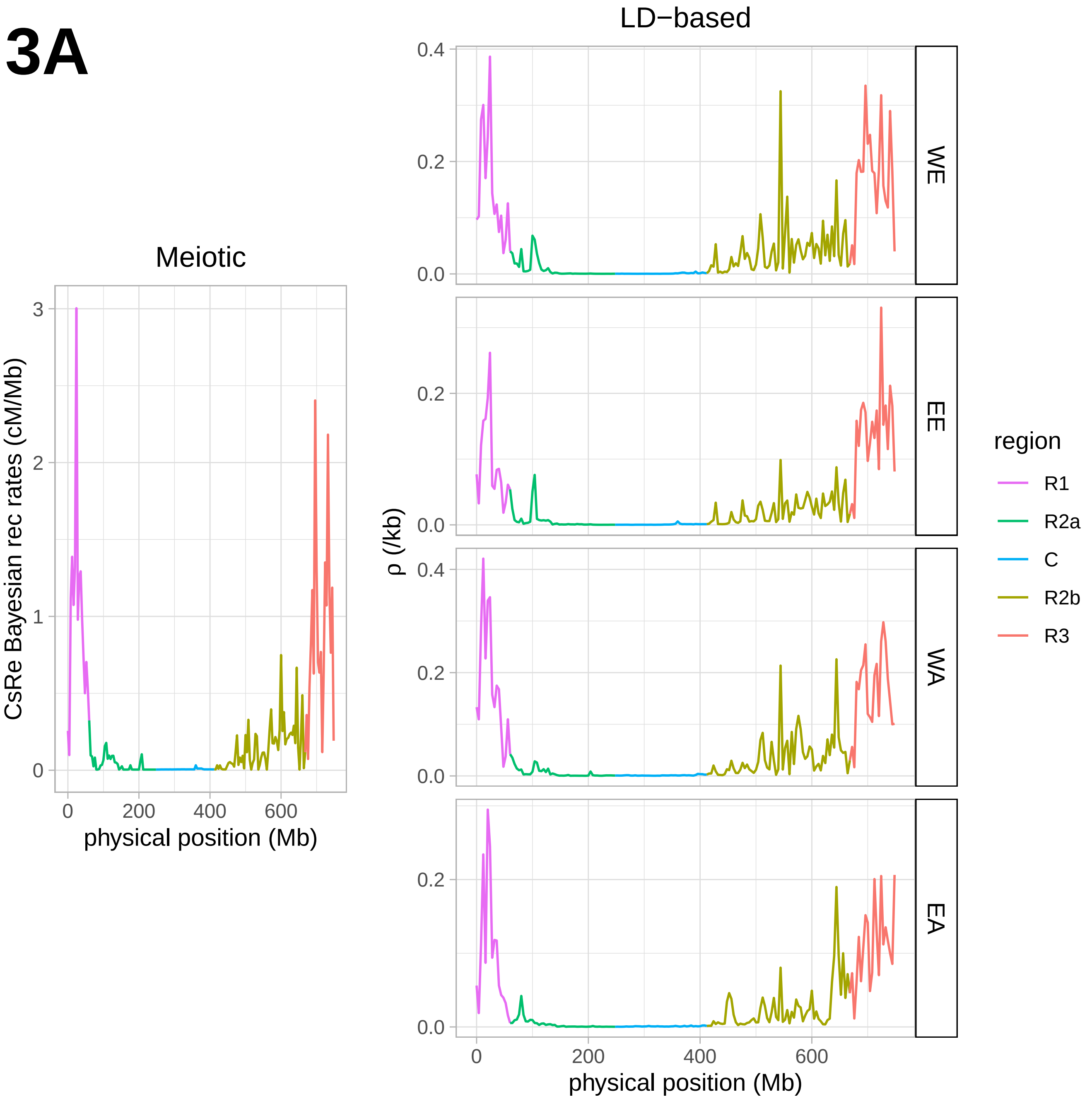

Meiotic
CsRe Bayesian rec rates (cM/Mb)
physical position (Mb)
LD−based
ρ (/kb)
WE
EE
WA
EA
region
R1
R2a
C
R2b
R3

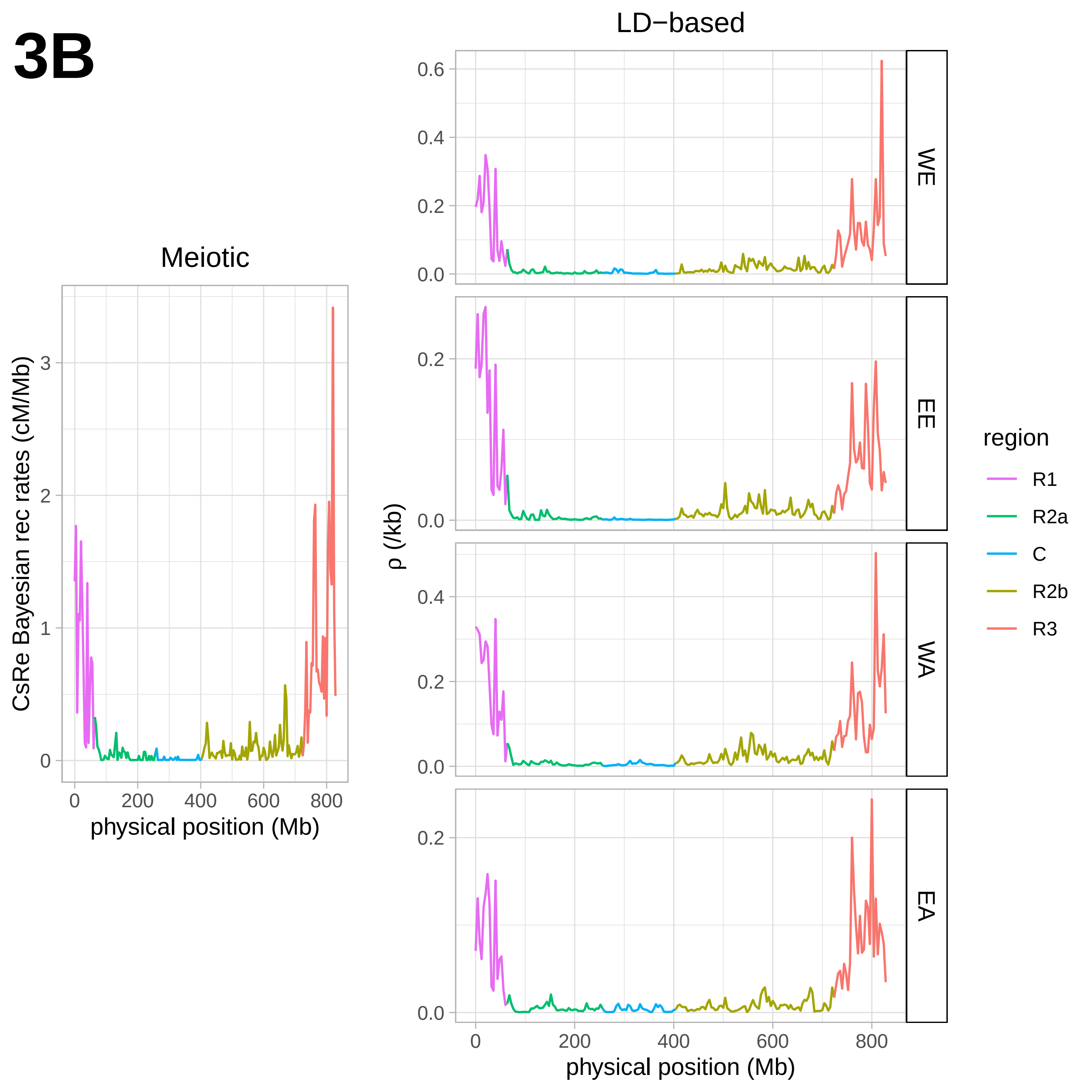
3B
Meiotic
LD−based
CsRe Bayesian rec rates (cM/Mb)
ρ (/kb)
physical position (Mb)
physical position (Mb)
WE
EE
WA
EA
region
R1
R2a
C
R2b
R3

3D

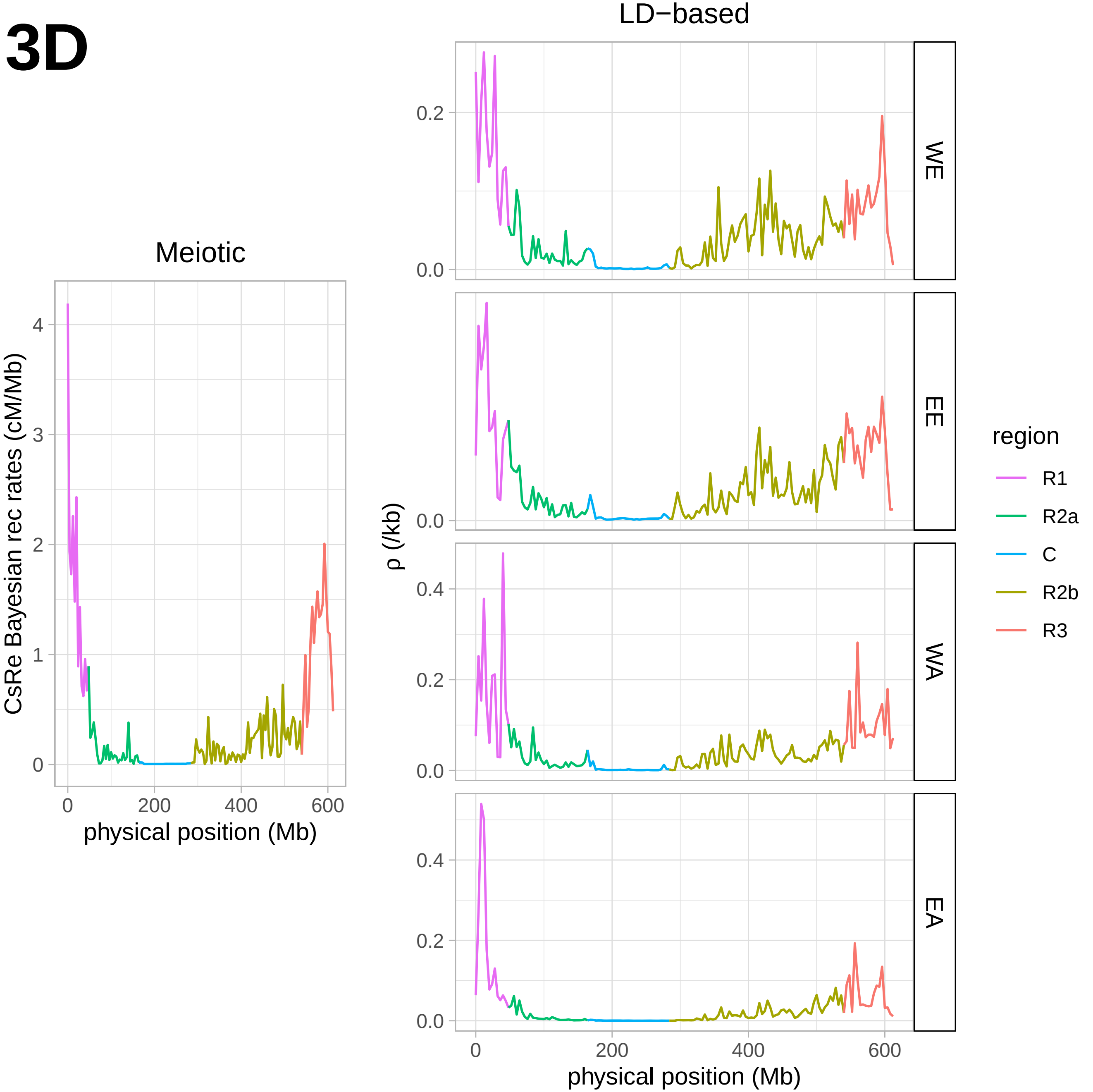

Meiotic
CsRe Bayesian rec rates (cM/Mb)
0
1
2
3
4
0
200
400
600
physical position (Mb)
LD−based
ρ (/kb)
WE
EE
WA
EA
0.0
0.2
0.4
physical position (Mb)
region
R1
R2a
C
R2b
R3

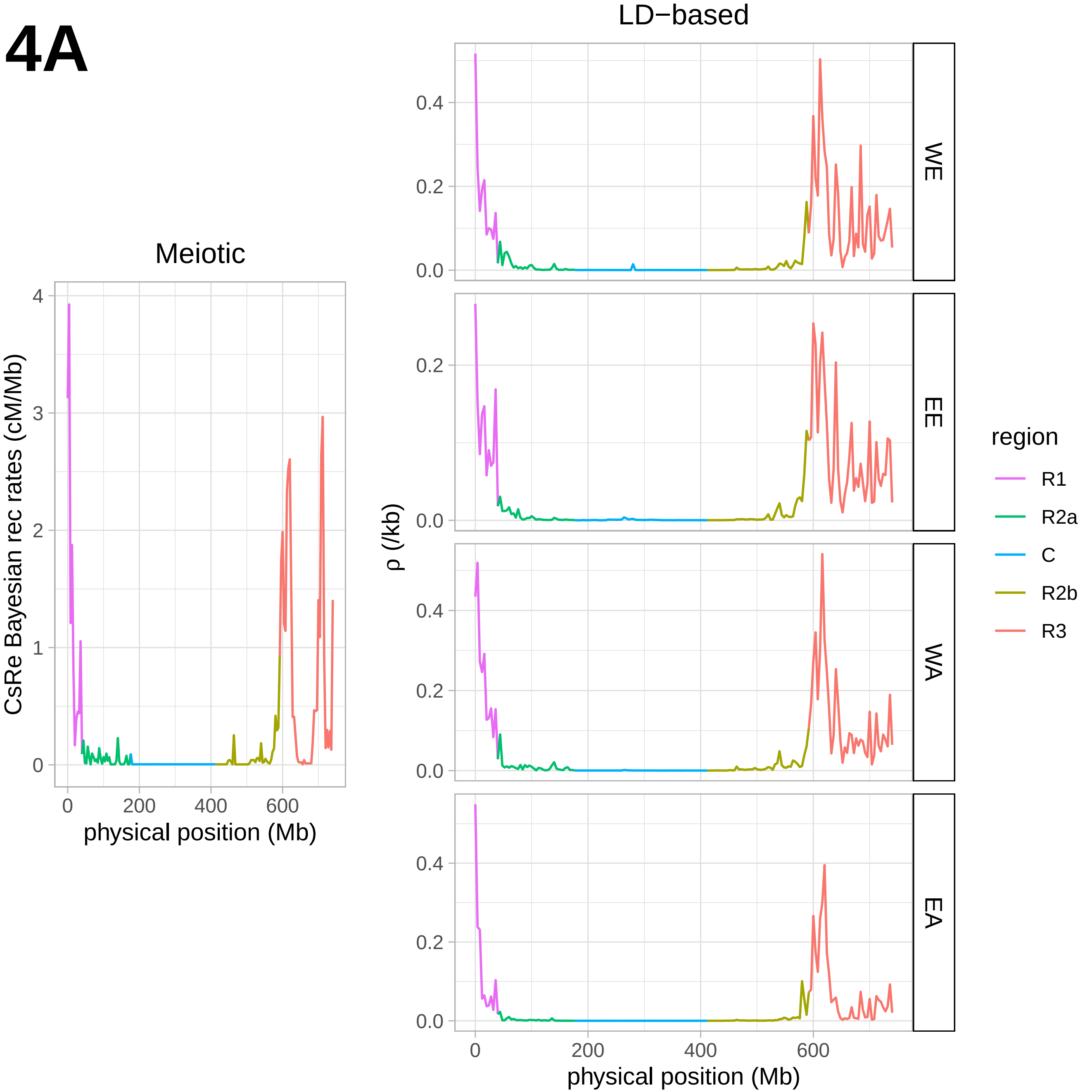

4A
Meiotic
LD−based
CsRe Bayesian rec rates (cM/Mb)
ρ (/kb)
physical position (Mb)
physical position (Mb)
0
1
2
3
4
0.0
0.2
0.4
0
200
400
600
WE
EE
WA
EA
region
R1
R2a
C
R2b
R3

4B

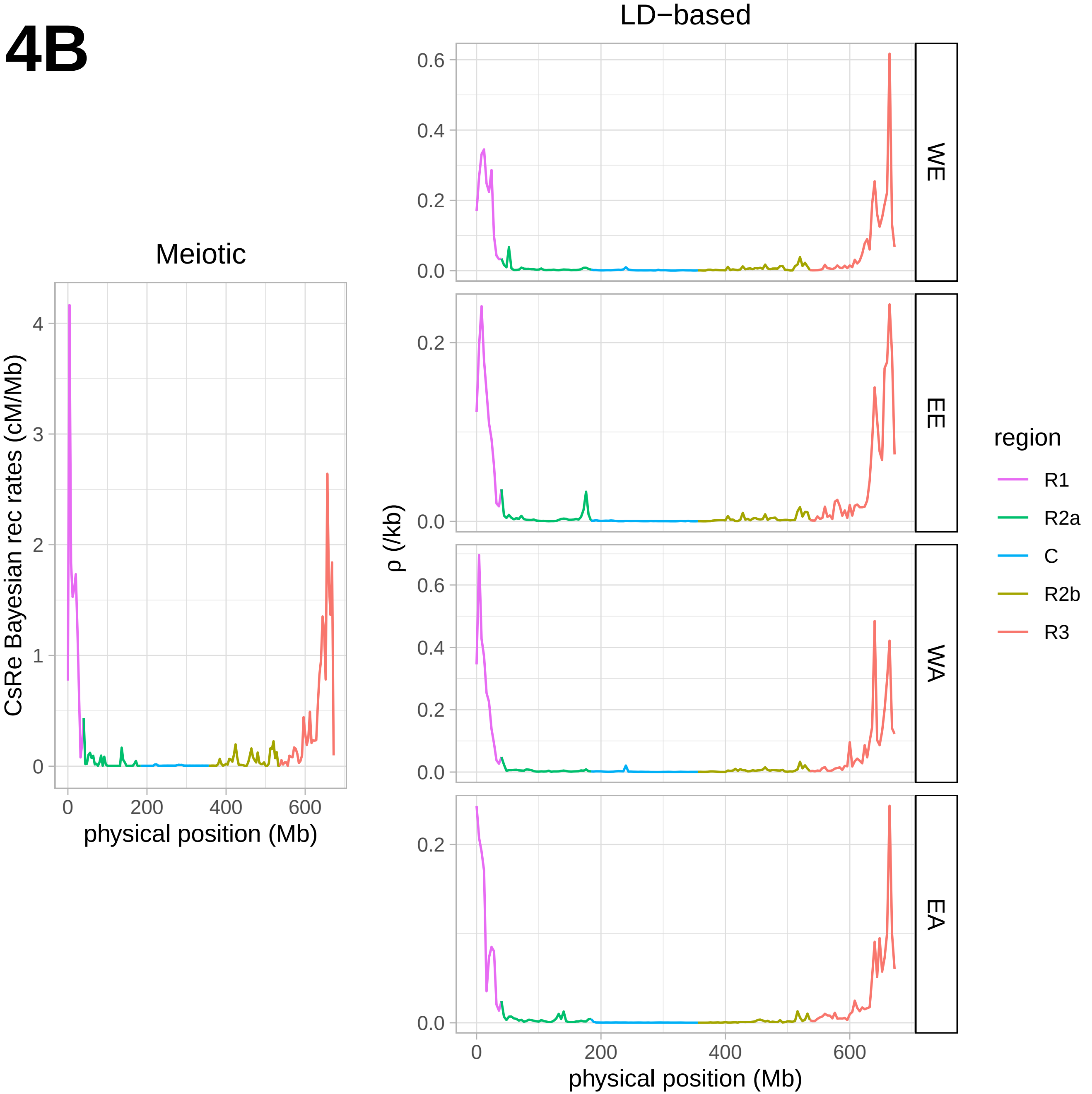

Meiotic
CsRe Bayesian rec rates (cM/Mb)
4
3
2
1
0
0
200
400
600
physical position (Mb)
LD−based
ρ (/kb)
WE
EE
WA
EA
0.6
0.4
0.2
0.0
0
200
400
600
physical position (Mb)
region
R1
R2a
C
R2b
R3

4D

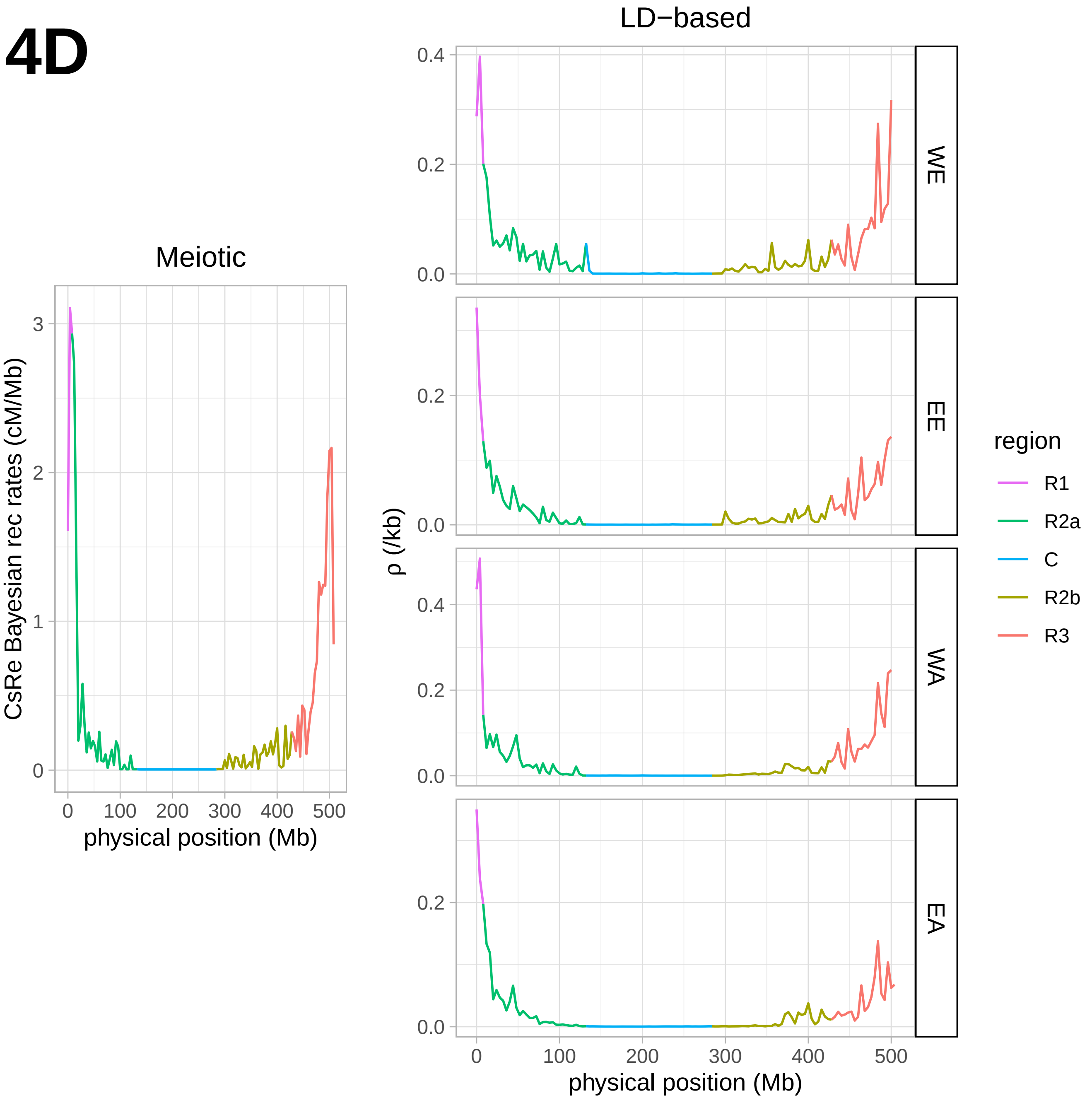

Meiotic
CsRe Bayesian rec rates (cM/Mb)
physical position (Mb)
LD−based
ρ (/kb)
WE
EE
WA
EA
region
R1
R2a
C
R2b
R3

5A

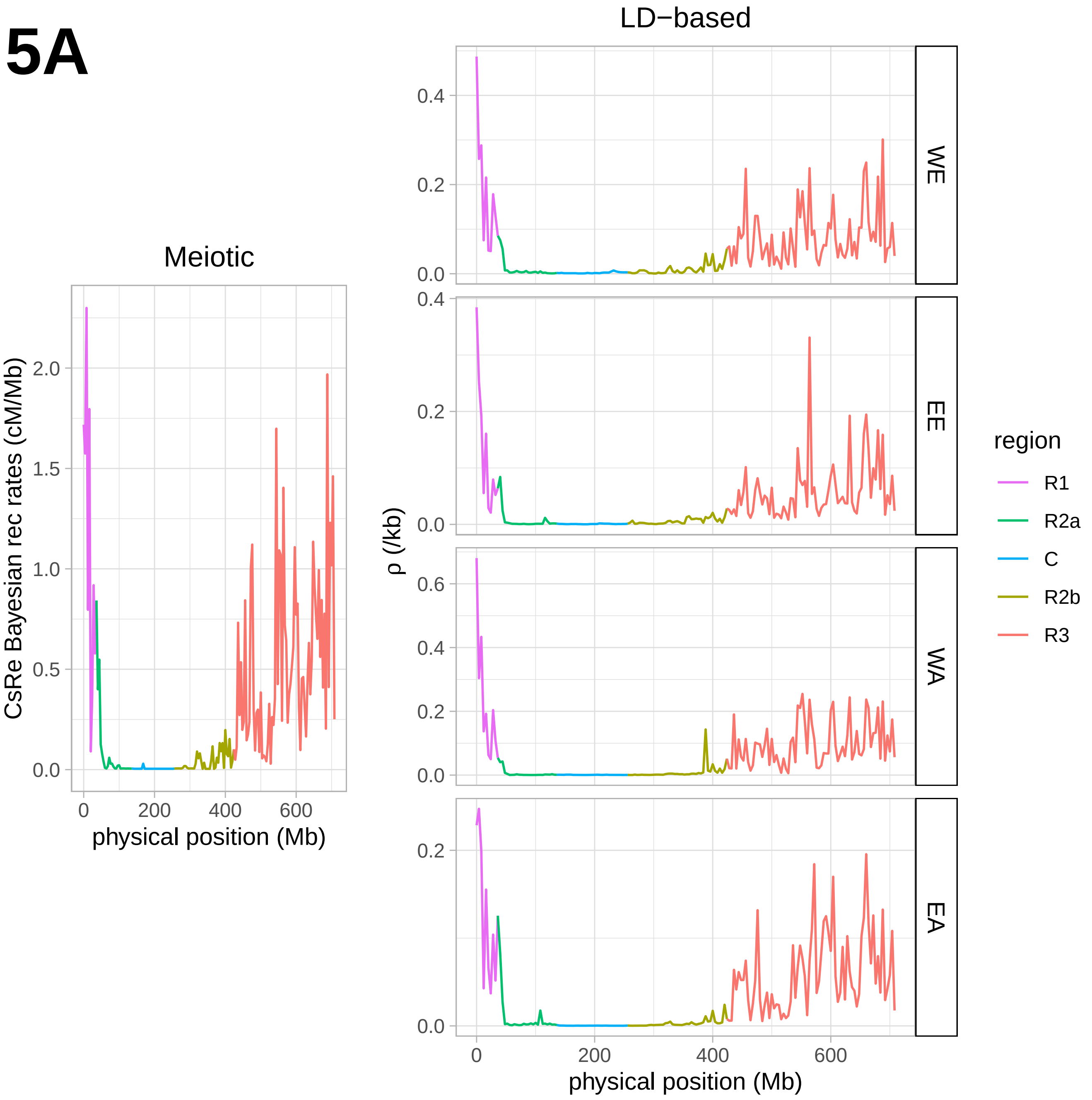

Meiotic
LD−based
CsRe Bayesian rec rates (cM/Mb)
2.0
1.5
1.0
0.5
0.0
0
200
400
600
physical position (Mb)
ρ (/kb)
WE
EE
WA
EA
0.4
0.2
0.0
0.6
region
R1
R2a
C
R2b
R3

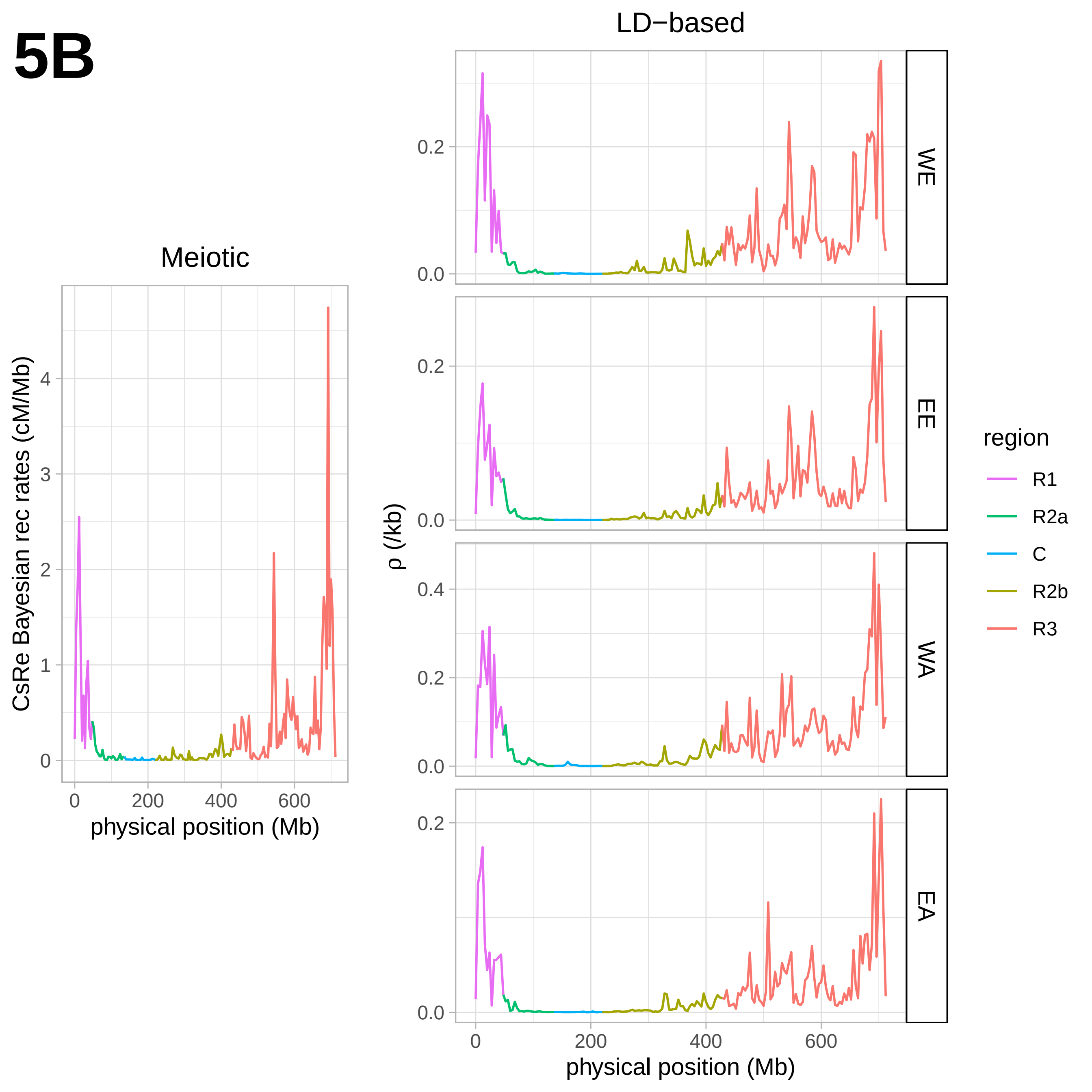
5B
Meiotic
LD−based
CsRe Bayesian rec rates (cM/Mb)
ρ (/kb)
physical position (Mb)
physical position (Mb)
WE
EE
WA
EA
region
R1
R2a
C
R2b
R3

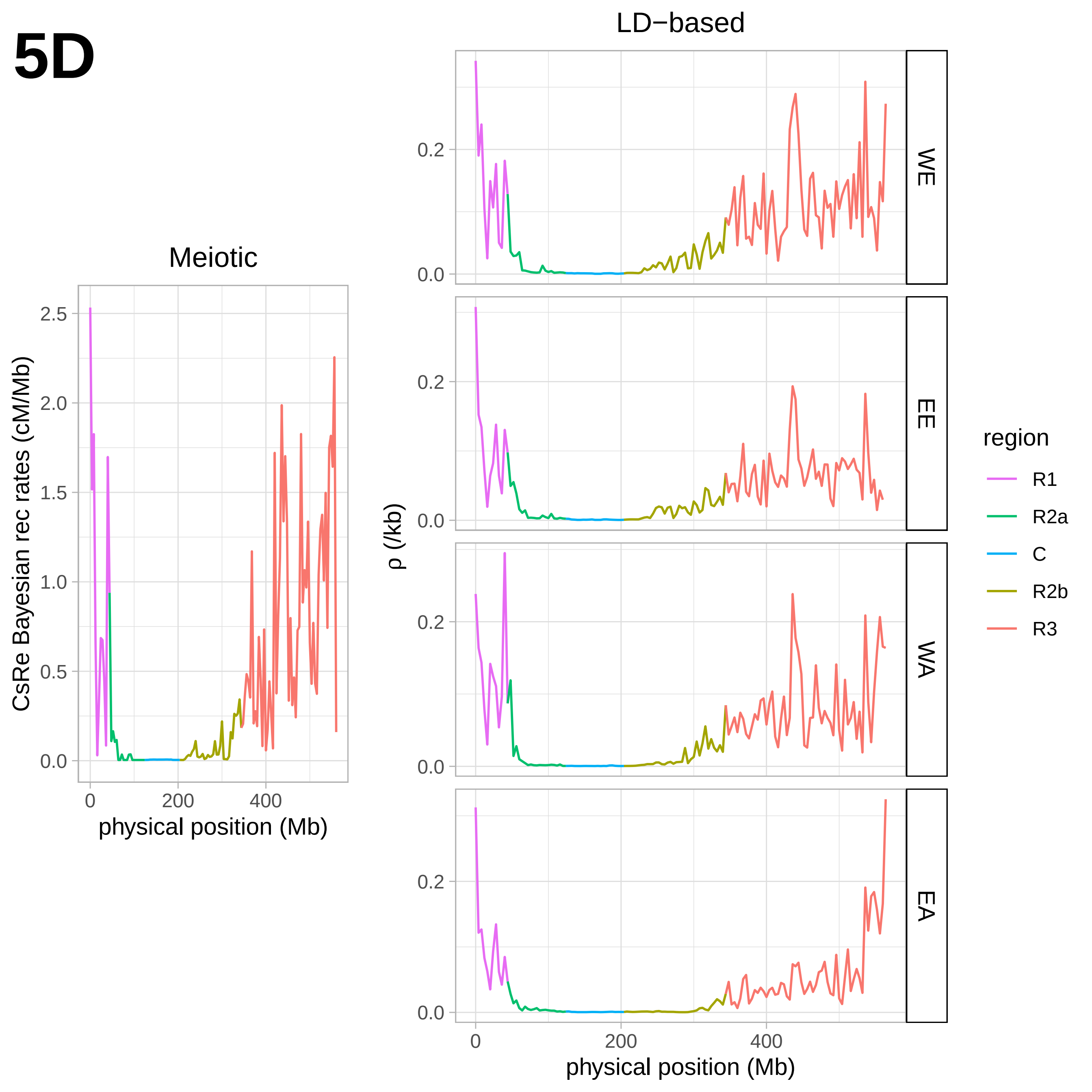

5D
Meiotic
LD−based
CsRe Bayesian rec rates (cM/Mb)
2.5
2.0
1.5
1.0
0.5
0.0
0
200
400
physical position (Mb)
ρ (/kb)
0.2
0.0
WE
EE
WA
EA
region
R1
R2a
C
R2b
R3

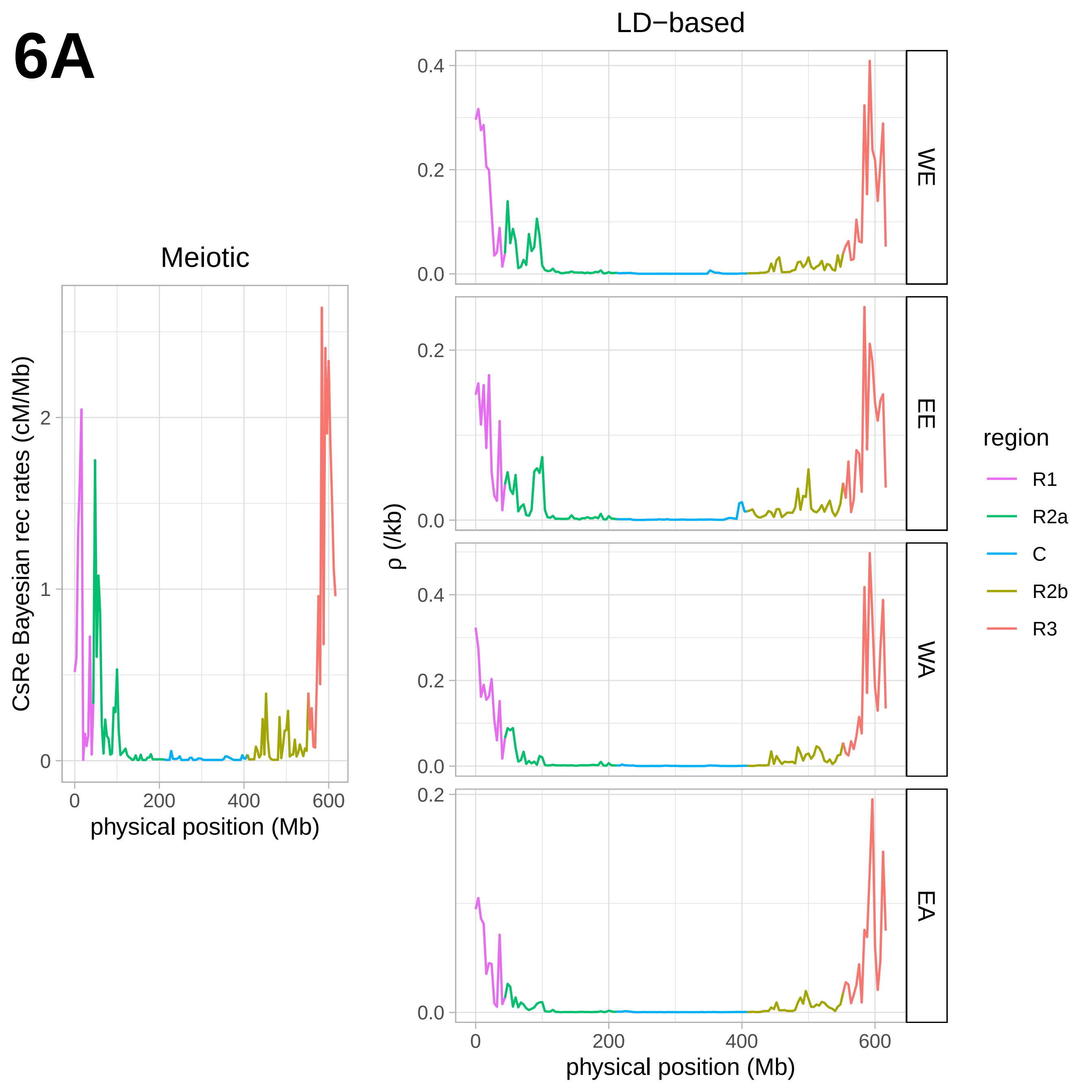

6A
Meiotic
LD−based
CsRe Bayesian rec rates (cM/Mb)
ρ (/kb)
physical position (Mb)
physical position (Mb)
WE
EE
WA
EA
region
R1
R2a
C
R2b
R3

6B

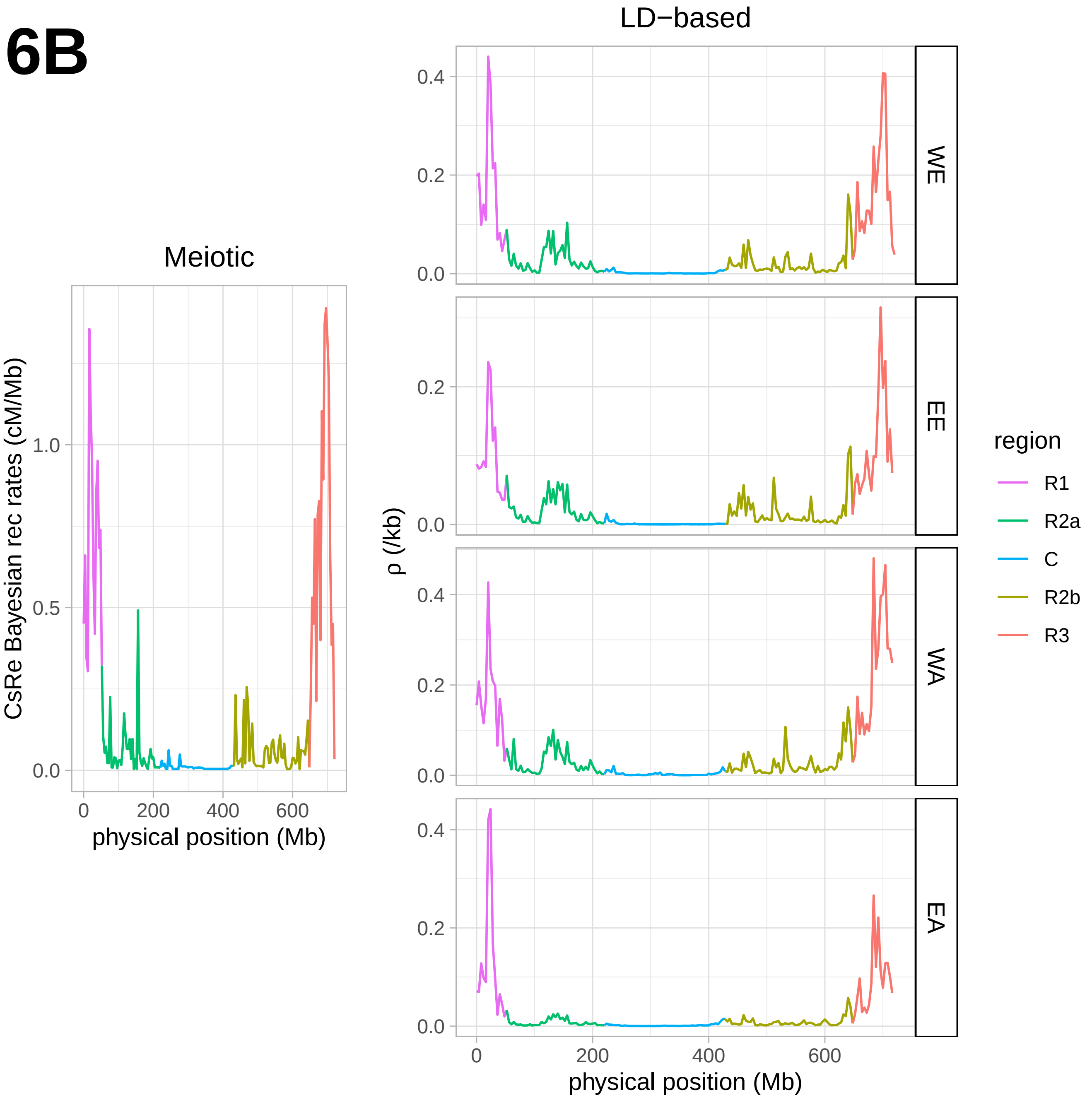

Meiotic
LD−based
CsRe Bayesian rec rates (cM/Mb)
physical position (Mb)
ρ (/kb)
WE
EE
WA
EA
region
R1
R2a
C
R2b
R3

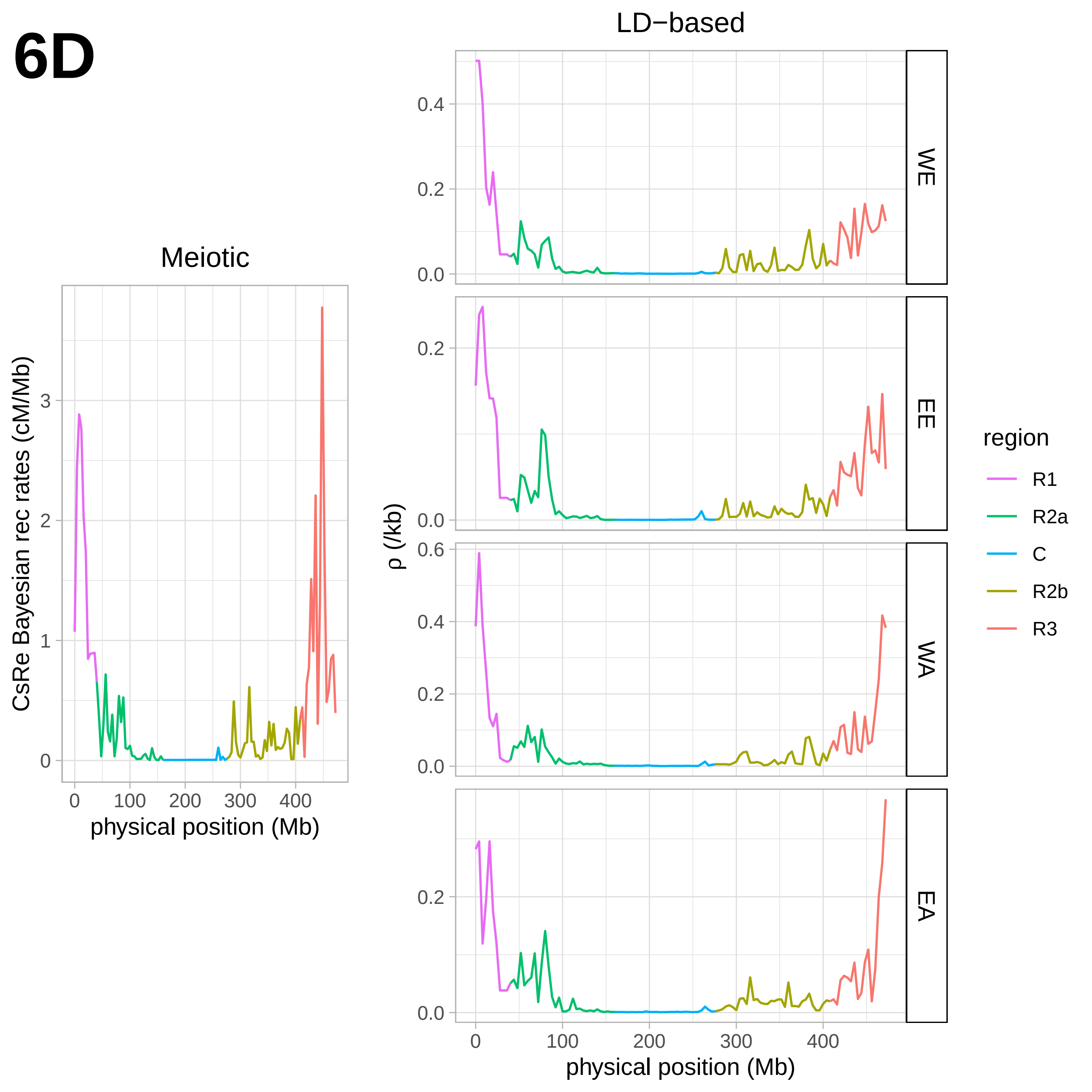

6D
Meiotic
LD−based
CsRe Bayesian rec rates (cM/Mb)
0
1
2
3
0
100
200
300
400
physical position (Mb)
ρ (/kb)
0.0
0.2
0.4
0.6
WE
EE
WA
EA
region
R1
R2a
C
R2b
R3

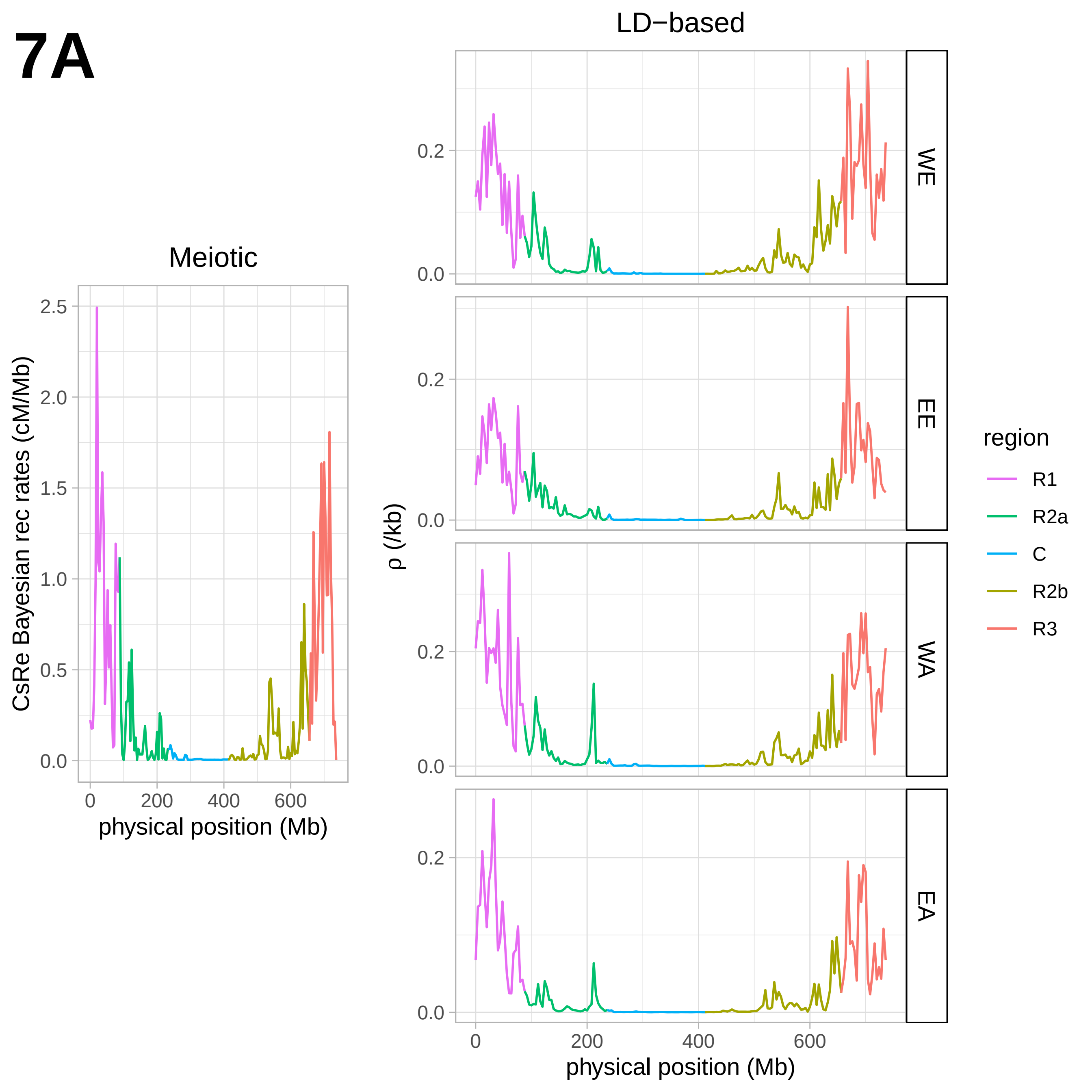

7A
Meiotic
LD−based
CsRe Bayesian rec rates (cM/Mb)
2.5
2.0
1.5
1.0
0.5
0.0
0
200
400
600
physical position (Mb)
ρ (/kb)
0.2
0.0
WE
EE
WA
EA
region
R1
R2a
C
R2b
R3

7B

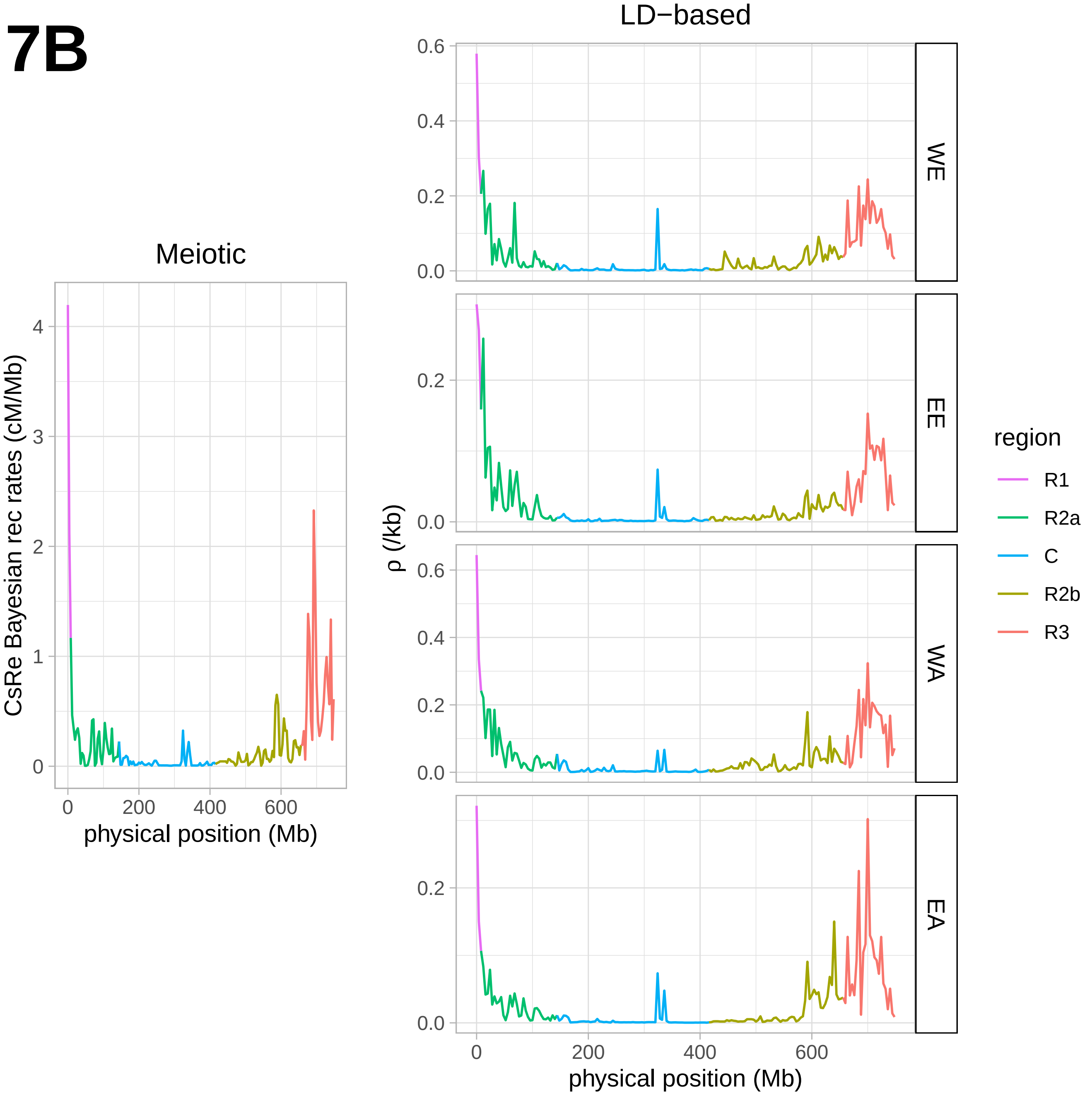

Meiotic
CsRe Bayesian rec rates (cM/Mb)
0
1
2
3
4
0
200
400
600
physical position (Mb)
LD−based
ρ (/kb)
0.0
0.2
0.4
0.6
WE
EE
WA
EA
0
200
400
600
physical position (Mb)
region
R1
R2a
C
R2b
R3

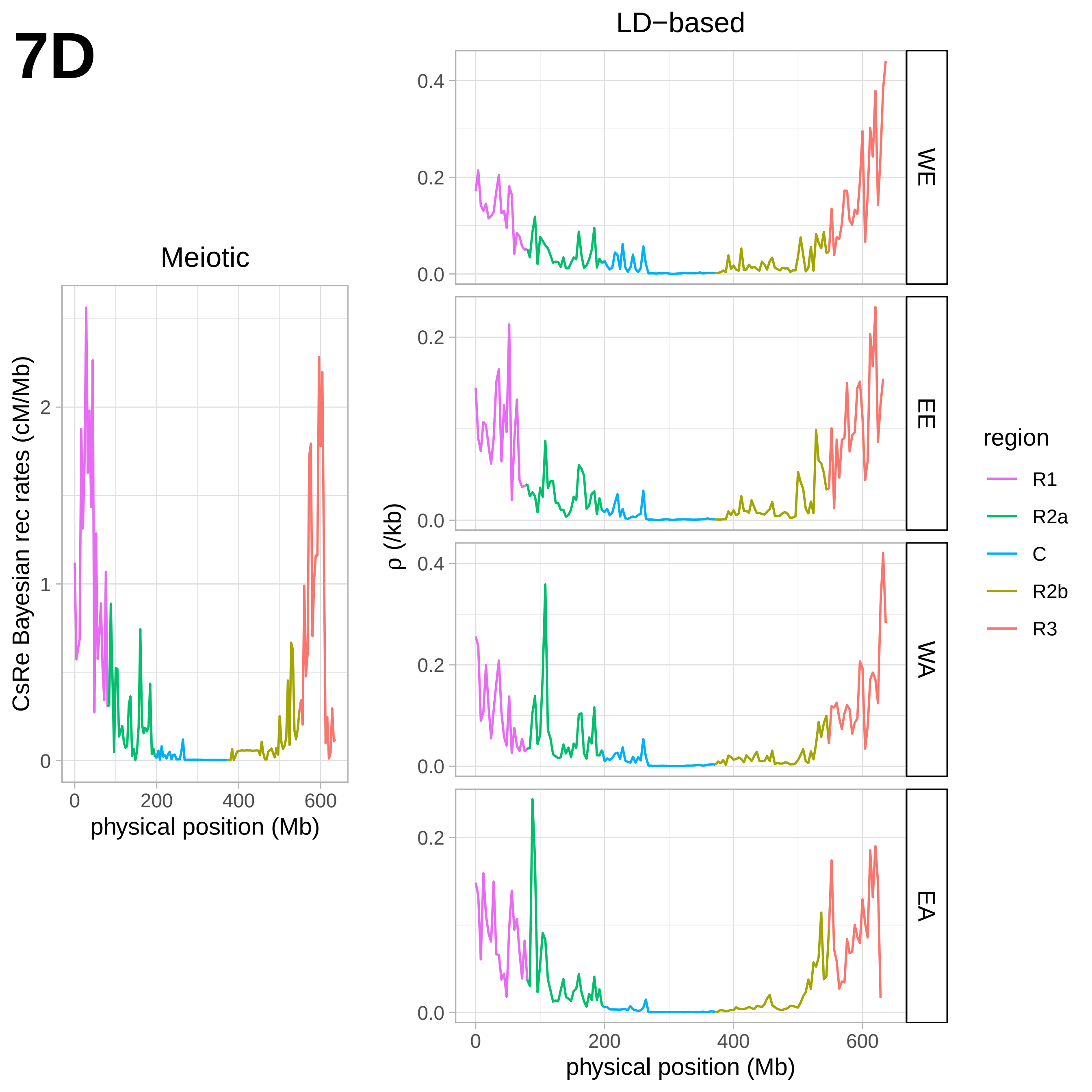

7D
Meiotic
CsRe Bayesian rec rates (cM/Mb)
physical position (Mb)
LD−based
ρ (/kb)
WE
EE
WA
EA
region
R1
R2a
C
R2b
R3
